# Non-Markovian dynamics and effective reproduction number in COVID-19: Evidence from Cyprus contact tracing data

**DOI:** 10.1371/journal.pcbi.1014578

**Published:** 2026-08-21

**Authors:** Pavlos Alexandros Dimitriou, Matteo D’Alessandro, Brian L. Chang, Valentinos Silvestros, Elisavet Constantinou, Costas Pitris, Panayiotis Kolios, Piet Van Mieghem

**Affiliations:** 1 University of Cyprus, Department of Electrical and Computer Engineering, Nicosia, Cyprus; 2 KIOS Research and Innovation Center of Excellence, Nicosia, Cyprus; 3 Delft University of Technology, Faculty of Electrical Engineering, Mathematics and Computer Science, Delft, The Netherlands; 4 Ministry of Health, Nicosia, Cyprus; 5 University of Cyprus, Department of Computer Science, Nicosia, Cyprus; The Second Xiangya Hospital of Central South University, CHINA

## Abstract

Using contact tracing data provided by the Cyprus Ministry of Health, infection trees for the first four waves of the COVID-19 epidemic are constructed. In these trees, nodes represent infected individuals, while links indicate the direction of transmission between them. For each infection tree of *N* nodes, the hopcount distribution from the root node to all other nodes is calculated. The empirical distribution is then compared to the hopcount distribution of infection trees generated by a non-Markovian SI process on a complete graph, with Weibull infection times characterized by a shape parameter α. We compute the values of the shape parameter α that best fit the empirical distribution and find that only values of α>1 are obtained, while the Markovian case is characterized by α=1. A Weibull distributed infection time with shape parameter α>1 is characterized by a unimodal density function with a peak at finite time, consistent with previous findings in the literature. Our analysis therefore suggests that the spreading process is most likely governed by non-Markovian dynamics, and that non-Markovianity can be detected solely from the topology of the infection trees. Finally, we analyze the evolution of the empirical distribution of the number of secondary infections caused by each node in the infection trees across different time windows to estimate the effective reproduction number. In practice, the average number of secondary infections seems to often provide a lower bound of the reproduction number computed by the Cyprus Ministry of Health. When the last level of the trees, composed predominantly of terminal nodes that do not generate further infections, is excluded, the estimate reflects more accurately the dynamics of the epidemic.

## Author summary

We explore how diseases spread through populations by examining infection trees. Each “node” in the infection tree is a person and each “link” is a transmission that specifies who infected whom during an outbreak. Based on contact tracing data from the COVID-19 epidemic in Cyprus, we develop a method to detect non-Markovianity in disease transmission by analyzing only the structure of these trees. A Markovian process assumes that the chance of spreading a disease depends only on an individual’s current state, whereas a non-Markovian process occurs if the timing of infections is influenced by the time passed since the host was first infected. Our results demonstrate that real disease spread exhibits non-Markovian behavior, which can be measured from the structure of infection trees without employing precise chronological information. We also estimate the effective reproduction number, which indicates how many new infections are generated on average by each infected individual. The agreement between our estimates and those reported by the Cyprus Ministry of Health confirms that working with detailed infection tree data is a valid and reliable way to study disease spread. Overall, our work provides a practical framework for understanding disease transmission in real populations.

## Introduction

The COVID-19 devastatingly impacted humanity, causing millions of deaths and placing unprecedented strain on healthcare systems worldwide [1]. However, despite these challenges, measurements during the pandemic have provided a unique opportunity to validate epidemiological models and gain deeper insights into disease dynamics. In this work, COVID-19 infection trees, which represent the chains of transmission reconstructed from contact tracing data in Cyprus, are investigated. By analyzing contact tracing information, researchers can uncover transmission patterns of outbreaks, reveal the impact of public health interventions and improve predictive models to better prepare for future outbreaks [2–8]. Cyprus’ status as an island, along with the implementation of strict border controls during the epidemic, created a uniquely contained setting that supported comprehensive contact tracing. In a previous work [9], an extensive literature review was conducted comparing studies about COVID-19 infection trees. The study shows that our dataset, spanning multiple epidemic waves, includes more reconstructed transmission trees than any other study to date, highlighting its uniqueness and completeness [9]. This unique scope enables an unprecedented level of analysis, offering valuable insights into the fundamental dynamics of spreading processes in human populations. We emphasize that assembling such a comprehensive dataset requires substantial resources, which explains why similar large-scale collections are rarely encountered in the literature [6,10,11].

Our main motivation is to measure from the infection trees how much the underlying spreading process departs from the assumptions employed in Markov theory. In Markovian epidemics, all the events in the spreading process happen independently at exponentially distributed random times, and therefore the epidemic itself is memoryless implying that the future states only depend on the actual viral state of the population [12]. Markovian epidemics produce nearly all currently used mean-field epidemic models. Hence, testing whether a Markovian description of reality is valid, is an important achievement. Real-world spreading events are very likely characterized by non-exponential infection and curing times [13–16] and evidence from COVID-19 measures [17,18] suggests that non-Markovian processes [19–22] with unimodal distributed infection times (e.g., Weibull or Gamma distribution) may give a better description of real processes [23].

Numerous studies have calculated different key statistics such as tree size, centrality metrics, the average number of secondary infections and superspreaders from contact tracing data [5,10,24], but little focus on the impact of the non-Markovianity of the spreading process on the tree structure [25]. We employ the equivalence between the non-Markovian susceptible-infected (SI) process in a contact graph and the shortest paths in a graph. In the SI process, susceptible nodes transition to an infected state upon transmission from a neighbor and remain infectious permanently, allowing the pathogen to traverse the full graph’s topology. A shortest path tree is equivalent to an infection tree (more details in Sec “Infection trees as shortest path trees”) if link weights correspond to infection times, where the infection time is the time needed for a just infected node to infect one of its direct, susceptible neighbors [26]. In our SI process, the time to infect a contact is assumed to be governed by a distribution with shape parameter α, which controls the memory effects in the spreading process. The parameter α is our “non-Markovianity” measure and here we confine to the shape parameter α of the Weibull distribution. When α=1, the infection time follows an exponential distribution, recovering the standard Markovian SI process (S2 Appendix). The Markovian infection trees are uniform recursive trees (URT): at each stage of the process a new node is infected uniformly by one of the existing infected nodes. URTs are characterized by recursive self-similarity [27] because each infection tree can be seen as a union of independent uniform recursive subtrees [28, Sec 16.2.2]. The recursive self-similarity is however “broken” whenever α≠1, and the tree structure becomes more complicated.

In this work, we extract the parameter α from the empirical hopcount distribution of a set of infection trees of the same number of nodes *N*. The hopcount is the number of links in the shortest path and the hopcount distribution indicates the probability that a path between two randomly chosen nodes in a graph *G* contains a specific number of links, called hops. The hopcount distribution was originally introduced in the early 2000s to study the hopcount of the shortest path between two arbitrary routers in the Internet [29,30]. Indeed, studying the hopcount between arbitrary nodes helps uncovering the topology, optimizing the network infrastructure, and proposing more efficient designs [31]. In network epidemiology [12], where nodes represent infected individuals and links indicate the direction of infection, the hopcount refers to the number of subsequent infections starting from patient zero (root node) to all other nodes in an infection tree.

## Methods

### Ethics statement

This study was approved by the Cyprus National Bioethics Committee (CNBC 2023.01.146). All data were anonymized, and the requirement for informed consent was waived by the ethics committee. We confirm that all methods were carried out in accordance with relevant guidelines and regulations.

### Contact tracing in Cyprus

In Cyprus, the first Health Information System for COVID-19 to support disease surveillance and control, named COVID-19 Emergency Response Platform (COVERP), was developed by the KIOS Center of Excellence at the University of Cyprus, and launched in March 2020 [32]. The timely implementation of this system, resulted in a complete and detailed record of the spread of the disease for the entire country, with 649000 infections in a population of 918100 individuals. Such data, for an entire closed population of almost one million individuals, with well controlled points of access, is not available elsewhere [9]. This situation is unique in Europe and probably internationally as well.

During the first two waves under analysis, contact tracing investigations were performed through telephone interviews from a team of 80 contact tracers. Contact tracers called each confirmed case and conducted investigations to identify the source of the infection and close contacts. For the third and fourth waves, investigations were performed via self-reports, in which confirmed cases declared their own contacts. Close contacts were then informed via SMS and were required to get tested in the following days.

Manual verification and validation were necessary to properly match cases and ensure that transmission trees were correctly reconstructed. The large number of daily tests, as shown in S1 Fig, with more than 80000 tests on some days, demonstrates the extensive testing capacity strengthening confidence that a large fraction of the infected individuals were identified. S2 Fig shows the changes in interventions during these waves providing contextual information on the level of public‑health restrictions during the analyzed periods. Obtaining a dataset of this scale and detail is challenging and requires significant financial and human resources. The process of identifying, testing and tracing each case demands extensive coordination between healthcare authorities, laboratories and contact tracing teams. Hence, most countries have not been able to reconstruct enough transmission trees, highlighting the uniqueness of our ensemble.

### Dataset

The dataset contains 33358 confirmed infections reported in Cyprus during the first four waves of the epidemic. Each infected individual was assigned a unique case ID, along with information on age, gender, occupation, residence location, date of positive test, and an infector’s ID indicating the direction of transmission. However, not all the infected individuals were able to identify their source of infection, resulting in 11577 isolated cases being excluded from the study. Table 1 shows the number of infections with available information about their source of infection, as well as the periods of each wave. The dataset comprises only the 31 days around the peak (maximum number of infected people) of each epidemic wave, and the intervals between waves are not part of the dataset. The time intervals analyzed in this study correspond to periods for which high‑quality, epidemiological validated contact‑tracing data were available. For the present study, the Ministry of Health provided only the case ID, date of positive test and infector’s ID from the dataset. Using this information, infection trees from the first four waves of the epidemic in Cyprus have been constructed.

**Table 1:** Information about the contact tracing dataset employed in our study for the COVID-19 epidemic in Cyprus. The second column displays the corresponding period. The third column indicates the total number of confirmed cases reported. The fourth column displays the number of cases and the relative percentage of the total cases which belong to an infection tree with at least two nodes.

Wave	Period	Total num. of infected	Num. of Infected in trees of size N≥2 (%)
Wave 1	Mar 18 – Apr 17, 2020	710	462 (65.1%)
Wave 2	Jul 16 – Aug 15, 2020	314	197 (62.7%)
Wave 3	Dec 13, 2020 – Jan 12, 2021	12345	7527 (61.0%)
Wave 4	Apr 4 – May 4, 2021	19989	13595 (68.0%)

### Infection trees

In the infection trees, the nodes represent infected individuals, while the links indicate the transmission direction between them. Each tree is constructed using data from the 31-day period around the peak (maximum number of daily infected individuals) of each epidemic wave, ensuring that infection chains are not pruned even if some infections occurred outside the studied time frame. Periods of strong epidemic growth are indeed characterized by denser contact-tracing data, and focusing on these intervals facilitates the reconstruction of infection trees. Isolated cases are instances where infected individuals could not provide information on whom they infected or by whom they were infected, and are excluded from the study. The constructed sets of infection trees are sparse and consist of numerous disconnected short transmission chains. Real‑world contact‑tracing data may contain missing links, due to unreported or untraced contacts. Previous analyses of SARS‑CoV‑2 transmission networks [7,9,33] show that incomplete information is common and naturally results in multiple disconnected transmission chains rather than a single tree. For the first wave, the whole set of infection trees includes 112 trees, ranging in size from 2 to 31 nodes. The second wave includes 33 trees, with the largest trees consisting of 31 nodes. In the third wave, 2402 trees range in size from 2 to 33 nodes, while the fourth wave involves 4164 trees, with maximum sizes reaching up to 27 nodes [9].

The number and size of reconstructed infection trees vary across epidemic waves. The variation can be attributed to several factors. Waves 1 and 2 were relatively small in overall case numbers, and testing during these waves relied primarily on polymerase chain reaction (PCR) tests, whereas waves 3 and 4 incorporated widespread rapid antigen testing. From Wave 3, the surveillance system also transitioned from telephone‑based interviews to electronic documentation, improving the completeness and structure of recorded transmission trees. In addition, transmission patterns may have changed across waves due to the implementation of different control measures, seasonal effects, and the circulation of distinct SARS‑CoV‑2 variants.

**Fig 1:**
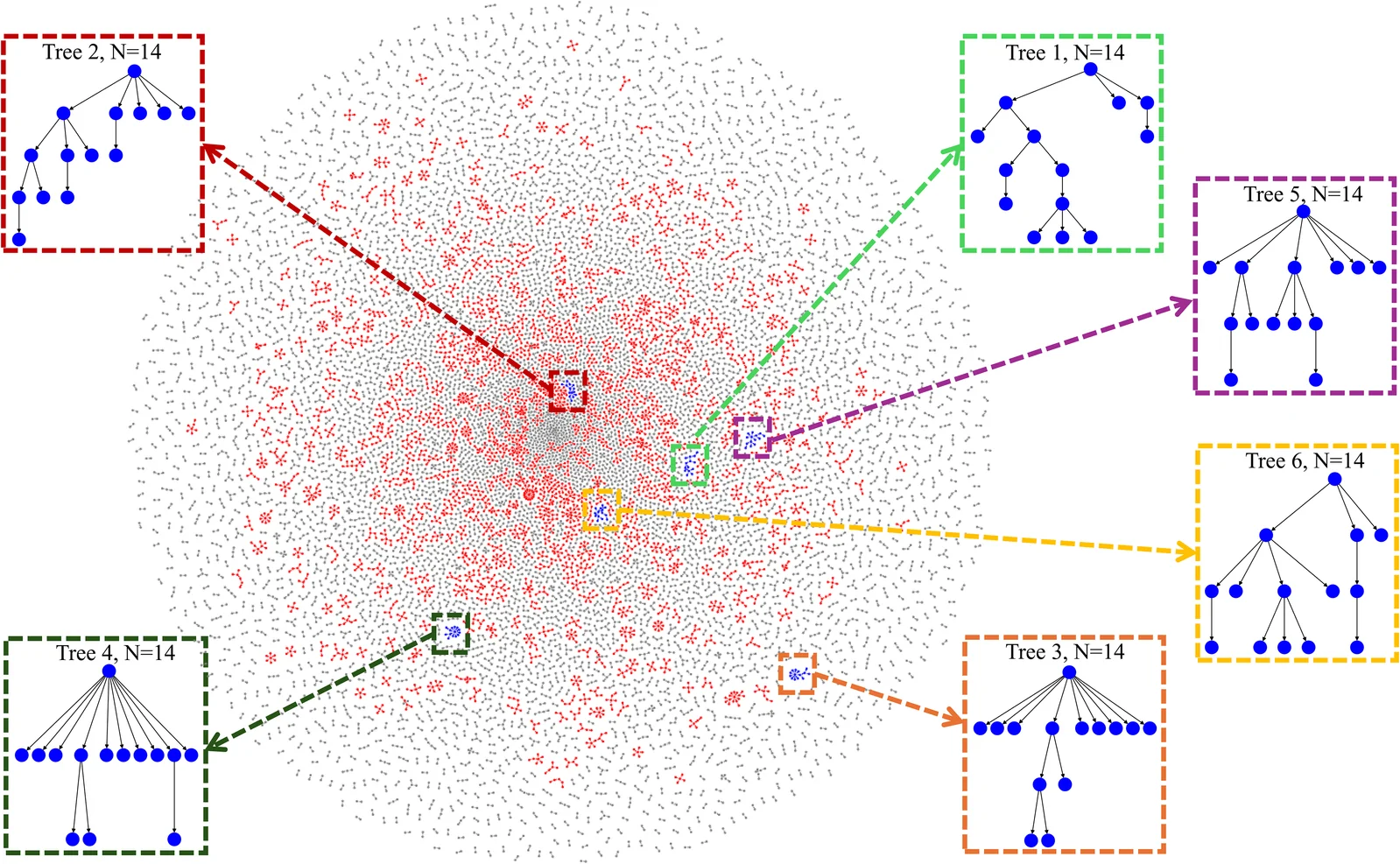
Infection trees of the fourth wave (April 2021 - May 2021) of the COVID-19 epidemic in Cyprus. The zoomed-in view displays a subset of the infection trees of size *N* = 14. Trees with less than 5 nodes are shown in gray, while larger trees (5 or more nodes) are shown in red.

Fig 1 shows the infection network during the fourth wave of the epidemic, in red the infection trees involving more than *N* = 5 infected individuals and in gray trees representing those with *N* < 5. The zoomed-in views highlight an example of the transmission chains with *N* = 14, where the root node (or patient zero) is at the top of the tree, and subsequent nodes are organized into levels representing the depth of the infection tree.

**Fig 2:**
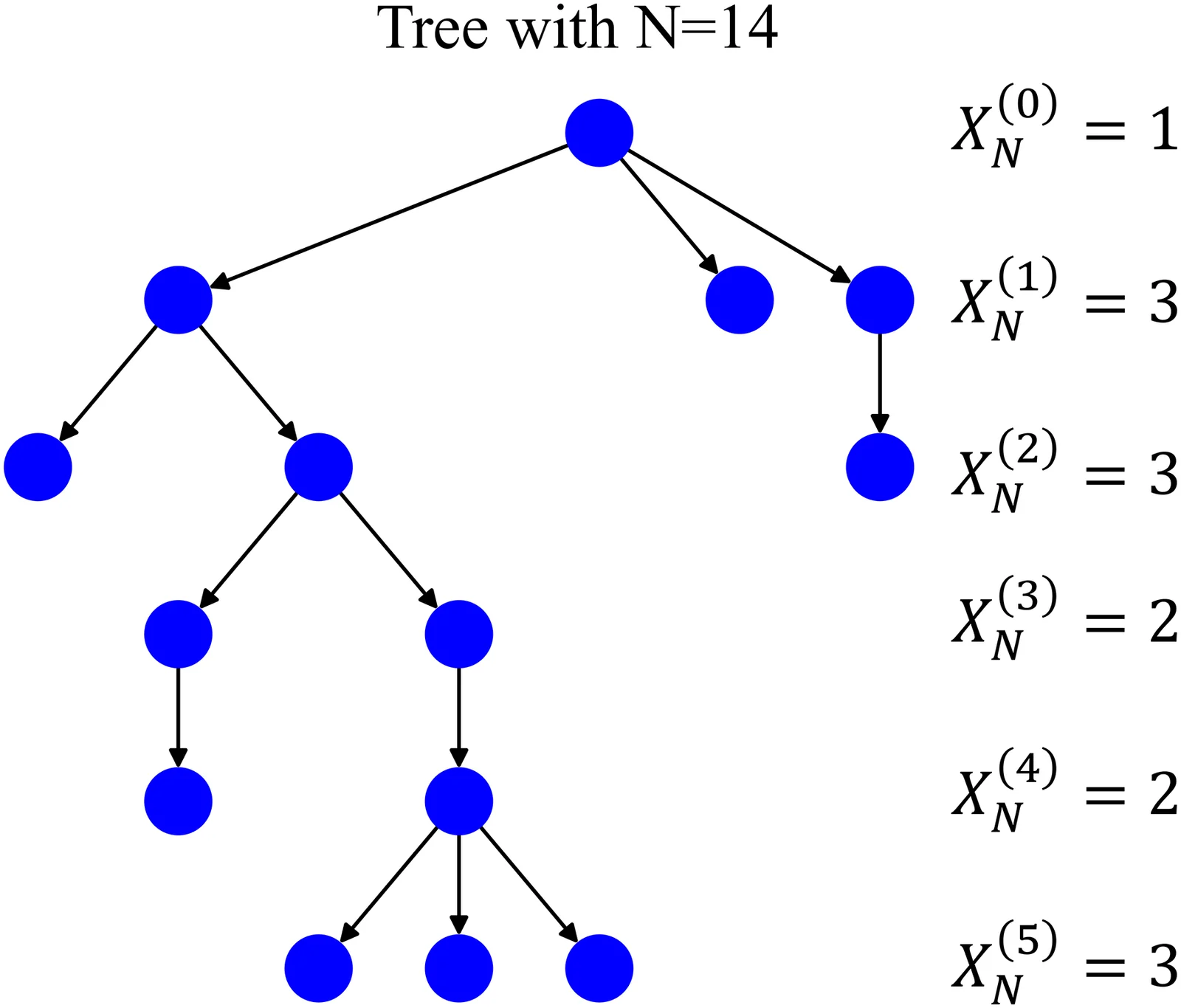
A real infection tree of the COVID-19 epidemic in Cyprus with *N* = 14 nodes. The variables $X_{N}^{\left(h\right)}$ represent the number of nodes located exactly at *h* hops from the root. For instance, at hopcount *h* = 1, there are $X_{14}^{\left(1\right)}=3$ nodes.

The hopcount defines the number of hops (links) along the unique path from the root to a specific node in the infection tree. By calculating the hopcount for every node, we can determine the level set, which represents the group of nodes across discrete distances from the root. Fig 2 displays an example of the hopcount of all the nodes belonging to a tree of size *N* = 14 in the dataset.

### Data limitations

The infection trees in the dataset are affected by intrinsic limitations:

1. The available contact tracing data only provide partial subgraphs of the global infection tree, offering an incomplete view of the overall transmission dynamics.
2. Contacts of infected individuals who tested negative are not reported, and only contacts who subsequently tested positive are included.
3. Given the limited time window selected, each infection tree only includes individuals which have been infected once.
4. The dataset does not distinguish between imported cases and local cases with unidentified sources; consequently, any individual without a traceable parent is treated as a new root node.

### Theory of infection trees

#### Infection trees as shortest path trees.

When analyzing an epidemic spreading on a graph *G* without re-infections, an infection tree (also known as transmission tree) is a directed acyclic subgraph of *G* whose nodes are the infected individuals and the links show “who infected whom,” starting from an initial case, the root or “patient zero” [34]. Two main properties of infection trees are investigated: (I) the distribution of the hopcount (i.e., the number of links along the shortest path) between the root and any other node in the infection tree; (II) the distribution of the outdegree of the nodes in the infection tree.

We employ the analogy between the shortest path problem on a graph *G* with independent and identically distributed (i.i.d) link weights and a susceptible-infected (SI) spreading process on *G* (see S2 Appendix for a rigorous definition of the Markovian process), where the infection times possess the same distribution as the link weights in the graph. The equivalence follows by viewing the transmission from the root to any node as finding the shortest path between them: assigning an independent infection time to each contact, the time at which an individual becomes infected is the minimum total infection time over all paths from the root, i.e. a shortest-path distance. Under the SI assumptions (no recovery/reinfection), the resulting parent-child relations form the corresponding shortest-path tree. The shortest path tree (SPT) [28, Chapter 16] is the union of the shortest paths from a source node to a set of other nodes in the graph *G*.

#### Markovian SI infection tree and shortest path tree with exponential link weights.

The shortest path tree in the complete graph $K_{N}$ with i.i.d exponentially distributed link weights is exactly equivalent to the infection tree of a homogeneous Markovian SI spreading process on $K_{N}$, where the link weights correspond to the exponentially distributed infection times (see S2 Appendix) with mean infection time 1/β. In our definition, an infection time is the random duration drawn for a transmission attempt along a specific link. Because the epidemic spreads through competing processes, a susceptible individual is ultimately infected by the neighbor whose drawn infection time is the shortest. Indeed, let us suppose that the root node *A* is infected at time *t* = 0 and that the infection rate of the process is β=1 for all the links between infected and susceptible nodes. The next node in *t*he infection tree, which we call *B*, is the one infected first by *A* whose infection time is the smallest. The infection times for all the other nodes, which are infected later in the process either by the root or by another infected node, are still exponentially distributed with rate β due to the memoryless property of the exponential distribution [28, p. 400–401]. Indeed, if node *A* infects node *B* at time *u*, the probability that node *A* infects another node *C* after time *t* + *u* is in general [28, p. 112]


$$\begin{array}{cc} Pr[T_{A\to C}>t+u|T_{A\to C}>u] & =\frac{Pr[\{T_{A\to C}>t+u\}\cap \{T_{A\to C}>u\}]}{Pr[T_{A\to C}>u]}\\ & =\frac{Pr[T_{A\to C}>t+u]}{Pr[T_{A\to C}>u]} \end{array}$$ (1)

where $T_{A\to C}$ is the random time at which *A* tries to infect *C*. If the infection times are exponentially distributed then (1) becomes


$$\begin{array}{c} Pr[T_{A\to C}>t+u|T_{A\to C}>u]=\frac{e^{-\beta (t+u)}}{e^{-\beta u}}=Pr[T_{A\to C}>t] \end{array}$$

and the probability that node *A* infects another node *C* after time *t* + *u* is equal to the probability that node *A* infects another node *C* after time *t*. Additionally, the minimum between independent exponential random variables is still an exponential random variable with rate equal to the sum of the single variable rates [28, p. 54]. Thus, the next infection in the Markov process will always happen at an exponentially distributed random time. Given that in a Markovian SI process all the infection link processes are independent, the sum of the infection times in the path of the SI infection tree between the root *A* and a given node *D*, is exactly the weight of the shortest path between *A* and *D* where the link weights are i.i.d exponentially distributed random variables with rate β. We can thus employ the well-known theory of the shortest path trees in the complete graph [28, Sec. 16.2], to devise the properties of the infection trees of a Markovian SI process on $K_{N}$. In particular, we exploit the fact that the shortest path tree in the complete graph with exponential link weights is a uniform recursive tree [28, Sec. 16.2]. A uniform recursive tree (URT) of size *N* is a random tree rooted at a given node *A*. At each stage a new node is attached uniformly to one of the existing nodes until the total number of nodes is equal to *N*. Due to the memoryless property of the exponential distribution and with all the infection link rates equal to β, the Markovian SI infection tree on the complete graph is a uniform recursive tree.

The hopcount $h_{N}$ in the URT of *N* nodes is defined as the smallest number of links between the randomly selected root *A* and a destination chosen uniformly from all the other nodes {1,...,N−1}. The exact probability density function of the hopcount in the URT with *N* nodes is [28, Corollary 16.3.2]


$$\begin{array}{c} Pr[h_{N}=k]=\frac{(-1{)}^{N-(k+1)}S_{N}^{\left(k+1\right)}}{N!}, \end{array}$$ (2)

where $S_{N}^{\left(k\right)}$ indicates the Stirling numbers of the first kind [35, 24.1.3]. The distribution (2) describes the hopcount distribution in the infection trees of a Markovian SI process on a complete graph with *N* nodes. For large *N*, to the first order, (2) is well approximated by a Poisson distribution [28, Eq. 16.14]


$$\begin{array}{c} Pr[h_{N}=k]\sim \frac{(\log N{)}^{k}}{Nk!}. \end{array}$$ (3)

#### Non-Markovian SI infection tree and shortest path tree with non-exponential link weights.

Let us consider the shortest path tree in a weighted complete graph $K_{N}$ with *N* nodes. If each link has an assigned random weight described by the random variable $W\overset{d}{=}X^{1/\alpha }$, where *X* is an exponential random variable $\text{Pr}[X\leq x]=1-e^{-x}$ with mean 1 and where α>0 is a positive shape parameter, then the distribution of the link weights corresponds to


$$\begin{array}{c} \text{Pr}[X^{1/\alpha }\leq x]=\text{Pr}[W\leq x]=1-e^{-x^{\alpha }}. \end{array}$$ (4)

The Weibull distribution is [28, Sec. 3.5.3]


$$\begin{array}{c} F_{W}(x)=Pr[W\leq x]=1-e^{-(\beta x{)}^{\alpha }}. \end{array}$$ (5)

and the related probability density function reads


$$\begin{array}{c} f_{W}(x)=\alpha \beta (\beta x{)}^{\alpha -1}e^{-(\beta x{)}^{\alpha }}. \end{array}$$ (6)

**Fig 3:**
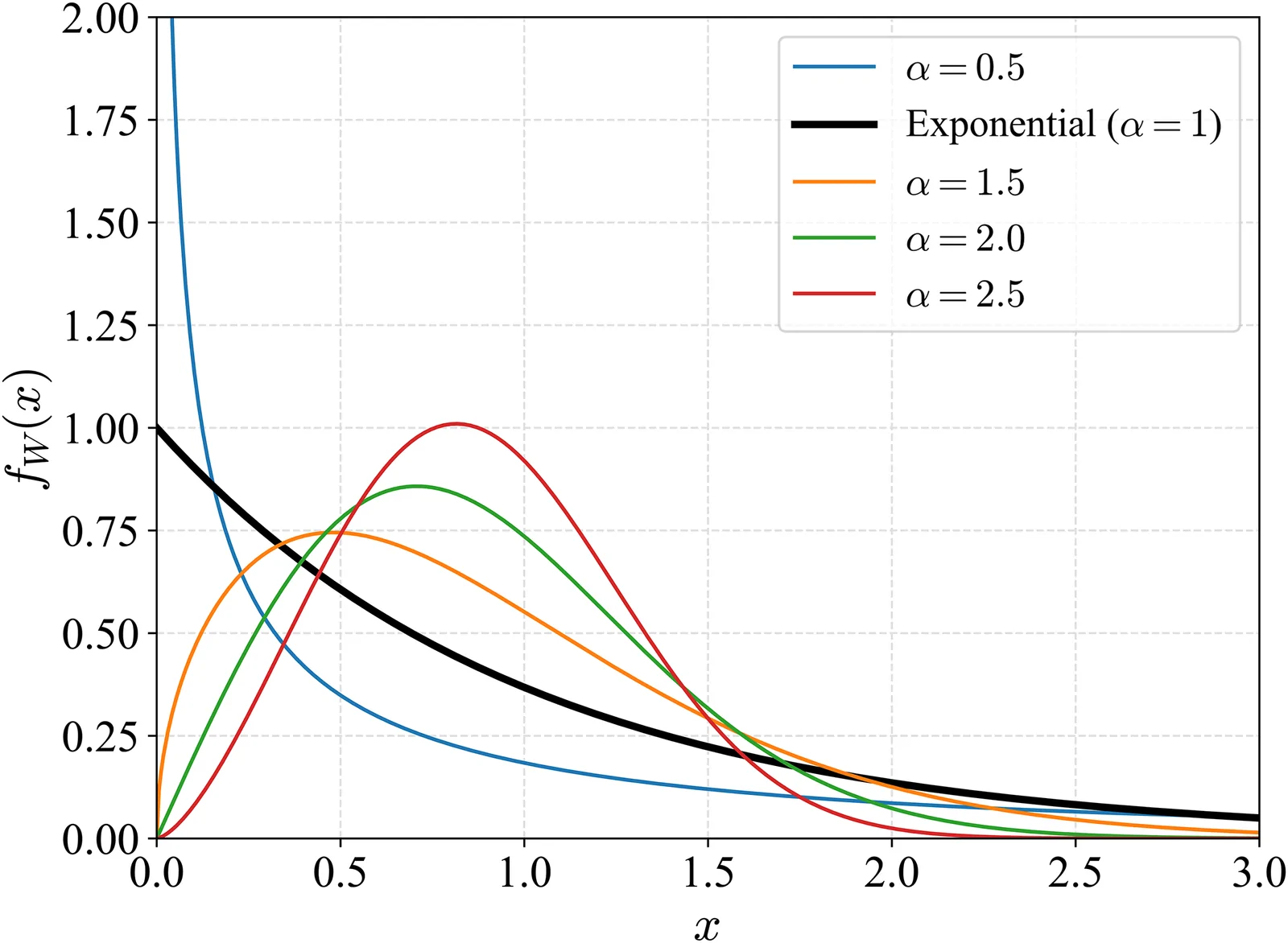
The Weibull probability density function (6) with rate parameter β equal to 1 and different shape parameters 0.5≤α≤2.5.

Equation (4) implies that the single link weights are distributed with a Weibull distribution with shape parameter α>0 and rate parameter β=1. Fig 3 shows the different properties of the Weibull density function between α smaller and bigger than 1. For α<1, the density function diverges at zero and becomes more heavy-tailed. For α>1, we observe a function with a single peak at *x* > 0 which has a root in the origin.

The hopcount distribution $\text{Pr}[h_{N}=k]$ (for integers k=0,...,N−1) of the shortest path tree with i.i.d. Weibull link weights is not known explicitly for any finite *N*, but has been computed [29, Sec. 6.1] asymptotically [29,36] for N→∞ as:


$$\begin{array}{c} \mathrm{Pr}[h_{N}=k;\alpha ]\approx \frac{1}{N}\frac{(\log\alpha N{)}^{\alpha k}}{\Gamma (\alpha k+1)}, \end{array}$$ (7)

which depends on the tree size *N* and on the shape parameter α. For α=1 equation (7) reduces to the Poisson distribution in (3). While in the Markovian case the relation between the shortest path trees and the infection trees is a natural consequence of the memoryless property of the exponential distribution and of the minimum between independent exponential random variables (see Sec “Markovian SI infection tree and shortest path tree with exponential link weights”), in a non-Markovian SI process on a graph the exact computation of the statistical properties of the infection trees is complex.

We replace the exponential distribution in the Markovian SI process presented in Sec “Markovian SI infection tree and shortest path tree with exponential link weights” with a Weibull distribution which is usually employed in non-Markovian models for spreading on networks [19–22]. The shape parameter α gives an indication of the non-Markovianity of the process [37] as for α=1 the pdf (6) is just an exponential with rate β. Contrary to the Markovian case α=1, the SI infection tree on the complete graph with Weibull infection times is not a uniform recursive tree. Suppose node *A* is infected at time 0 and it tries to infect its neighbors *B* and *C*. If the infection attempt towards *B* is successful the random infection time $T_{A\to B}^{\left(\alpha \right)}=u$ and $T_{A\to C}^{\left(\alpha \right)}>u$ because the infection of node *C* from *A* will certainly happen after a time *u*. We thus define a new random infection time from *A* to *C*, conditioned on the event $T_{A\to C}^{\left(\alpha \right)}>u$, as the residual random variable $R_{u;A\to C}^{\left(\alpha \right)}=T_{A\to C}^{\left(\alpha \right)}-u$, whose distribution function is


$$\begin{array}{c} \text{Pr}[R_{u;A\to C}^{\left(\alpha \right)}\geq t]=Pr[T_{A\to C}^{\left(\alpha \right)}>t+u|T_{A\to C}^{\left(\alpha \right)}>u]=\frac{e^{-(\beta (t+u){)}^{\alpha }}}{e^{-(\beta u{)}^{\alpha }}}, \end{array}$$ (8)

having employed (1) with the Weibull distribution (4). For α≠1, the infection probability depends on the history of the infections and the process is clearly non-Markovian.

The process of building the shortest path tree in a complete graph $K_{N}$ with Weibull distributed i.i.d weights, produces a shortest path tree without the symmetry of the uniform recursive tree in the exponential weights setting. Indeed, if the link weight $w_{A,B}$ from the root *A* to a given node *B* is the smallest among all the links from *A*, we are sure that the link weight from *A* to another node *C* is such that $w_{A,B}<w_{A,C}$. Choosing $w_{A,B}=u>0$ and knowing that $w_{A,C}$ is Weibull distributed, implies that


$$\begin{array}{c} Pr[w_{AC}>t+u|w_{AC}>u]=\frac{e^{-(\beta (t+u){)}^{\alpha }}}{e^{-(\beta u{)}^{\alpha }}}\neq Pr[w_{AC}>t]=e^{-(\beta t{)}^{\alpha }}, \end{array}$$

equivalent to the expression (8) obtained for the non-Markovian SI process. With non-exponential link weights, the process of building the shortest path tree on a graph is equivalent to the non-Markovian SI process on the same topology.

### Reproduction number and infection trees

The basic reproduction number *R*_0_ is defined [38] as the expected number of secondary cases produced, in a completely susceptible population, by a *typical* infected individual during its entire period of infectiousness. After sufficiently long time, a non-zero fraction remains infected if *R*_0_ > 1, else the epidemic will die out if *R*_0_ < 1 [12,39]. A rigorous computation of the *R*_0_ requires the definition of a *next generation operator*, which describes how many secondary cases arise from an individual which generally belongs to a heterogeneous population characterized by a given distribution. The basic reproduction number *R*_0_ is defined as the dominant eigenvalue of the next generation operator [38]. Alternatively, *R*_0_ can be defined from compartmental disease transmission model based on a system of differential equations [40].

The classic definition of the basic reproduction number usually ignores the underlying contact network between individuals in a population [28, 40, Sec 17.1] and can often be replaced by the epidemic threshold. The epidemic threshold $\tau_{c}$ represents the strength of the epidemic process and depends on the underlying contact network. In the mean-field approximation $R_{0}=\tau \lambda_{1}$, where $\lambda_{1}$ is the spectral radius of the adjacency matrix of the underlying contact network [28, Sec. 17.3].

Estimating the basic reproduction number *R*_0_ or the epidemic threshold $\tau_{c}$ from real-world data is complex, because it requires a detailed understanding of various population characteristics, such as age, sex, and other demographic factors, as well as their influence on disease transmission. Additionally, it necessitates the knowledge of the full contact network between individuals, the temporal evolution of the number of susceptible individuals, and potential external influences like vaccination rates or behavioral changes in response to the epidemic [41]. Indeed, when the basic reproduction number *R*_0_ is computed from compartmental models, many assumptions [38,40,42] are needed.

If infection trees are available, then the reproduction numbers can be estimated from data itself [4–6,24] with minimal assumptions. In an active epidemic, it is often more practical to employ the effective reproduction number $\hat{R}$, defined as the actual average number of secondary cases generated per primary case [5]. For any individual *i* within an observed infection tree, the number of secondary infections is directly represented by their outdegree. By considering a specific temporal window $W_{\Delta t}=(t-\Delta t,t]$, we can compute the outdegree distribution for all cases tested positive within $W_{\Delta t}$. The mean of this distribution provides the empirical effective reproduction number $\hat{R}(t;\Delta t)$ for that period. The knowledge of the full outdegree distribution also allows to extract information about the variance of the individuals’ infectivity of the spreading process, often overlooked when considering average quantities [24].

### Modeling assumptions

In view of the limitations (Sec “Data limitations”) that characterize the infection trees in the dataset, we propose the following assumptions to model the data using our theory (Sec “Theory of infection trees”):

1. Each infection tree of size *N* nodes is an independent realization of the non-Markovian SI process on a complete graph $K_{N}$ with *N* nodes (Sec “Non-Markovian SI infection tree and shortest path tree with non-exponential link weights”). The infection time from an infected to a susceptible node is assumed to be Weibull distributed with probability density function (6), rate parameter β=1 for each link and shape parameter α. In our definition of the non-Markovian SI epidemic, each infection process is assumed to be independent from the other infection processes in the network.
2. Each infection tree is assumed to represent a complete spreading process. If the tree has size *N*, we assume that the infection tree is the resulting transmission chain within a fully connected community of *N* individuals, where one individual is initially infected and the spreading process continues until all *N* individuals have become infected.

Overall, our framework provides a minimal model to detect and characterize non-Markovian dynamics in the empirical spreading process.

### Estimate of non-Markovian dynamics from infection trees

In this section we summarize the two methods employed to measure the non-Markovianity of the infection trees through the estimation of the parameter α from the infection tree structure. A more comprehensive description is given in S4 Appendix. All the simulated infection trees are generated using the Dijkstra’s algorithm [43], as explained in S3 Appendix.

#### Method 1.

**Fig 4:**
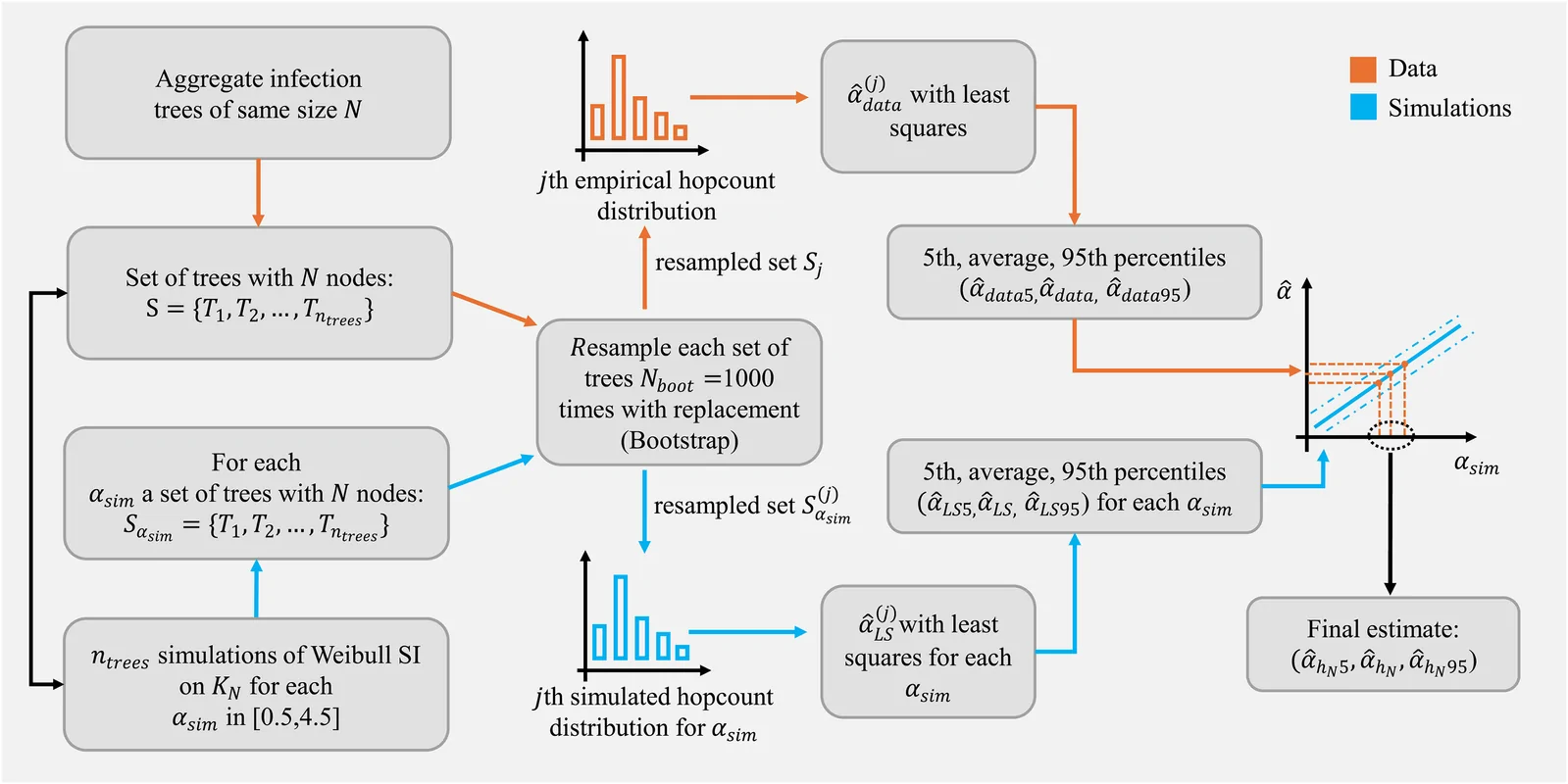
Flow chart of Method 1 based on hopcount distribution, details in S4 Appendix.

In each wave we create a set *S* aggregating trees of the same size *N* and for each set *S* we compute the hopcount distribution


$$\begin{array}{c} \hat{Pr}[h_{N}=k|data]=\frac{1}{N}\frac{1}{n_{trees}}\sum_{i=1}^{n_{trees}}(X_{N}^{\left(k\right)}{)}_{i}, \end{array}$$ (9)

where $(X_{N}^{\left(k\right)}{)}_{i}$ is the level set at hopcount *k* of the *i*-th tree of size *N* in the set *S*. We then fit the empirical hopcount distribution with (7) extracting the non-Markovianity parameter α with a least square method. Each set of trees is resampled with replacement $N_{boot}=1000$ times obtaining $N_{boot}$ new resampled set of infection trees of the same size *N*. For each resampled set $S_{j}$, with $j=1,...,N_{boot}$, a least squares estimate ${\hat{\alpha }}_{data}^{\left(j\right)}$ of the shape parameter is obtained. From the distribution of the $N_{boot}$ estimates of the non-Markovianity parameter α, we extract the average, the 5th and the 95th percentiles. The three values are then adjusted comparing the least square estimates computed with (7) from the data, with the least square estimates of α computed on simulated set of infection trees. The simulated trees are obtained from the simulation of the SI process on a complete graph with fixed *N* and selected shape parameter $\alpha_{sim}\in [0.5,4.5]$ for the infection time. Fig 4 presents an overview of the method.

#### Method 2.

**Fig 5:**
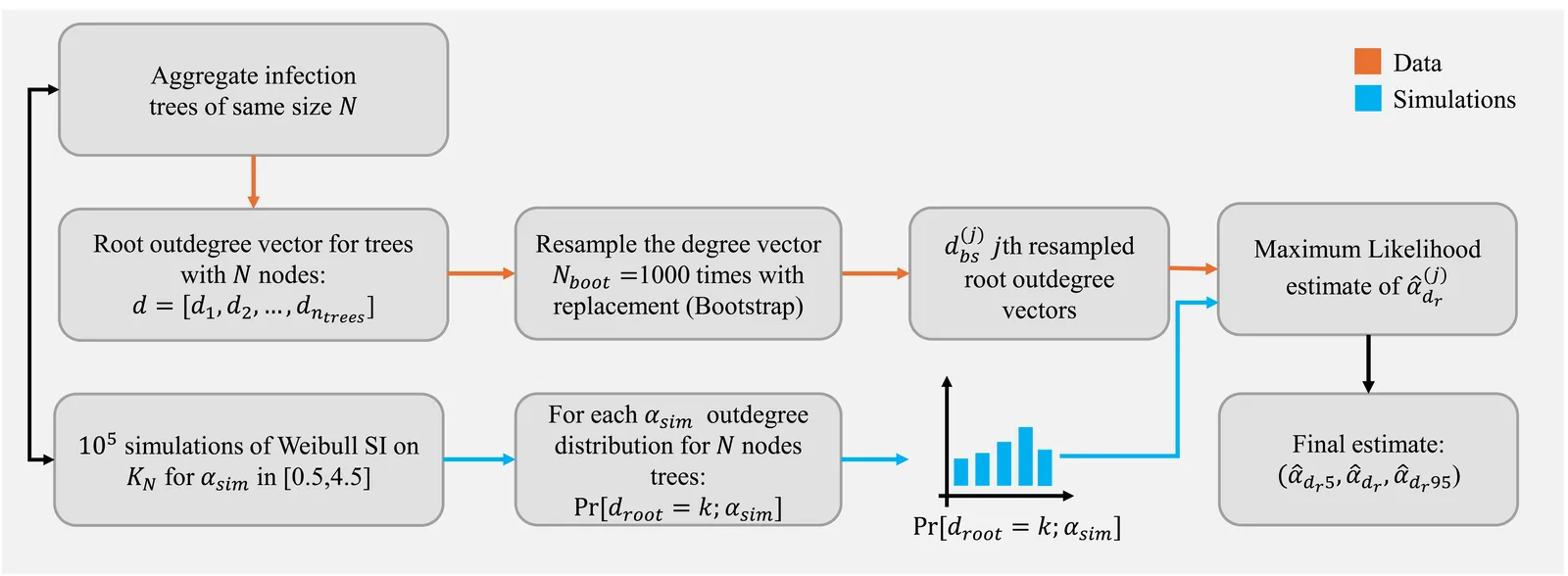
Flow chart of Method 2 based on root outdegree distribution, details in S4 Appendix.

In each wave we aggregate trees of the same size *N* and we compute the empirical outdegree distribution of the roots of the trees. We resample the trees of same size *N* with replacement and we derive $N_{boot}=1000$ bootstrapped root outdegree distributions. For each resampled distribution ($j=1,...,N_{boot}$), we make a maximum likelihood (see Eq. (S9) in S4 Appendix) estimate ${\hat{\alpha }}_{d_{r}}^{\left(j\right)}$ of the shape parameter α, maximizing the log-likelihood of observing the resampled empirical root outdegree distribution given the simulated distribution $\hat{\text{Pr}}[d_{root}=k|\alpha_{sim}]$ (with $\alpha_{sim}$ values in the range [0.5,4.5]). The average and the percentiles of the $N_{boot}$ estimates give the final value ${\hat{\alpha }}_{d_{r}}$ of the shape parameter α with confidence intervals. Fig 5 presents an overview of the method.

To ensure the reliability of our methods, we performed a comparative study of their statistical power and robustness (see S4 Appendix and S5 Fig). The analysis confirms that both methods accurately track the underlying shape parameter α and exhibit comparable statistical power (probability of rejecting the Markovian hypothesis given a true non-Markovian set of data).

### Effective reproduction number from the evolution in time of the outdegree distribution

We examine the distribution of outdegrees in infection trees, that is the number of secondary cases attributed to each infected individual. Analyzing the distribution within a given time window captures how the infection trees reflect the evolution of the epidemic spreading. We define the (dynamic) effective reproduction number $\hat{R}(t;\Delta t)$ as the average number of secondary infections caused by individuals who were tested positive within the time window $W_{\Delta t}=(t-\Delta t,t]$. Such quantity enables direct comparison with reproduction number estimates obtained from alternative modeling approaches applied to the same epidemic in Cyprus [42,44]. We complement the effective reproduction number $\hat{R}(t;\Delta t)$ with a 95% confidence interval obtained resampling $N_{boot}=1000$ times the outdegree distributions of the confirmed cases in the time window $W_{\Delta t}$.

Our definition of the dynamic effective reproduction number $\hat{R}(t;\Delta t)$ is not suitable for real-time monitoring purposes. This is because its computation relies on complete information about the secondary cases generated by individuals who tested positive within the selected time window, including infections that may have been recorded after the window has closed. A real instantaneous reproduction number would require taking into account the period of infectivity of a single individual and its contact network (or at least its average number of contacts), which would necessitate additional assumptions and information that are not available for our dataset.

Because our dataset contains no periods of unmitigated transmission without external intervention (S2 Fig), we cannot isolate the basic reproduction number *R*_0_ under natural conditions. Consequently, this study focuses exclusively on the effective reproduction number $\hat{R}$, which reflects actual transmission dynamics.

## Results

### Infection trees show non-Markovian properties

**Fig 6:**
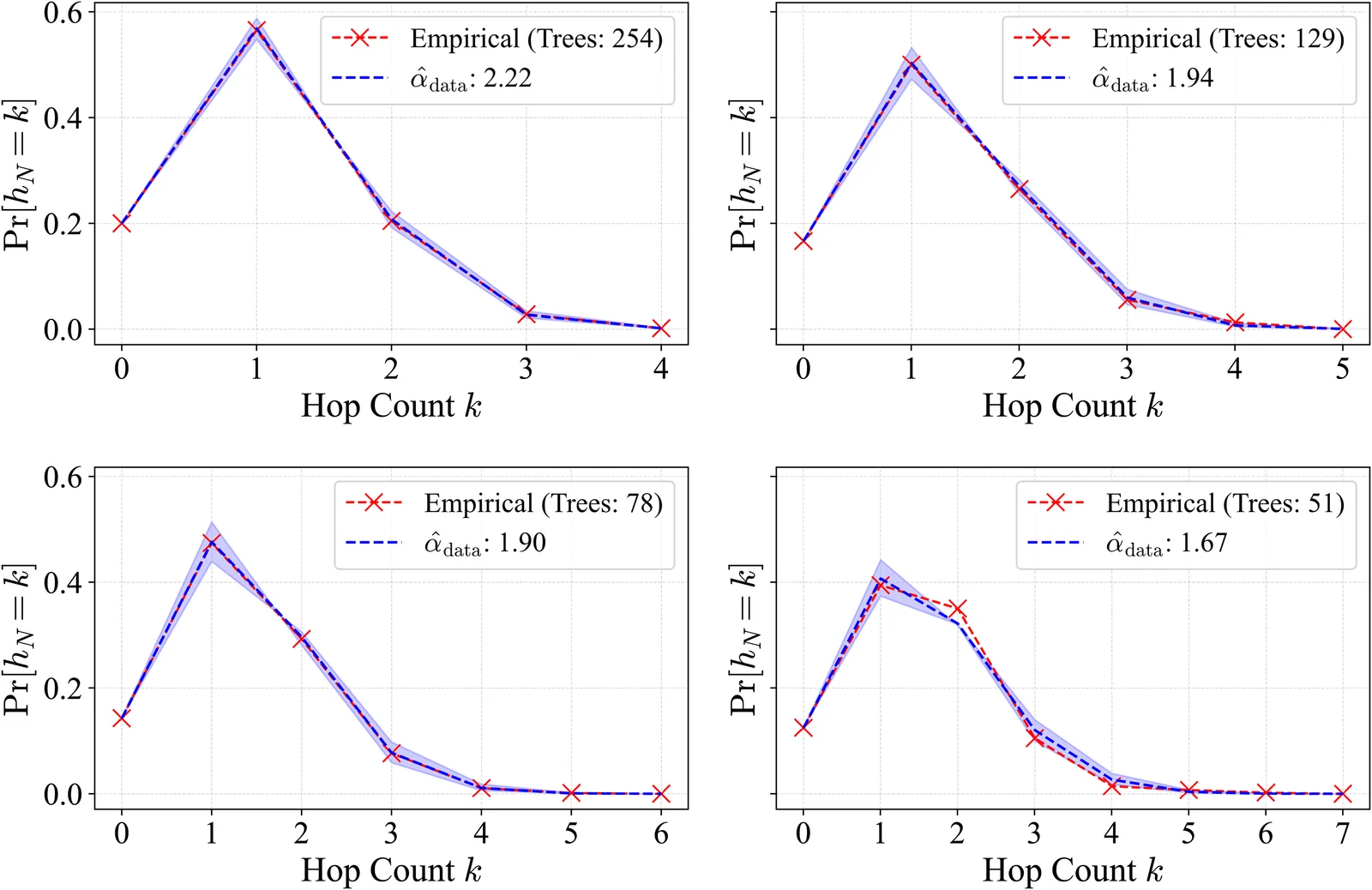
Hopcount distribution for infection trees belonging to wave 4 of size *N* = 5,6,7,8. Red line: empirical hopcount distribution obtained aggregating trees of the same size *N*. Dashed blue line: distribution (7) with parameter ${\hat{\alpha }}_{data}$, which corresponds to the average of the least squares estimates ${\hat{\alpha }}_{data}^{\left(j\right)}$, *j* = 1,...,1000. The blue area spans the distributions (7) with shape parameter α in the 90% confidence intervals of the least square estimate ${\hat{\alpha }}_{data}$.

Fig 6 shows the hopcount distribution of the infection trees in wave 4 with size *N* = 5,6,7,8. These tree sizes represent the most frequently observed sizes in the empirical data. In red the empirical hopcount distribution is displayed. In dashed blue the distribution with parameter ${\hat{\alpha }}_{data}$, that is the average of the estimates of ${\hat{\alpha }}_{data}^{\left(j\right)}$ with *j* = 1,...,1000 obtained on the resampled empirical distributions. The blue area fills the space between the two distributions with parameters equivalent to the 5th and the 95th percentiles of the distribution of the bootstrapped estimates.

The least squares estimates ${\hat{\alpha }}_{data}$ extracted from the empirical distribution are then corrected employing the hopcount distribution resulting from the simulation of the SI process. The detailed correction procedure is displayed in S3 Fig.

**Fig 7:**
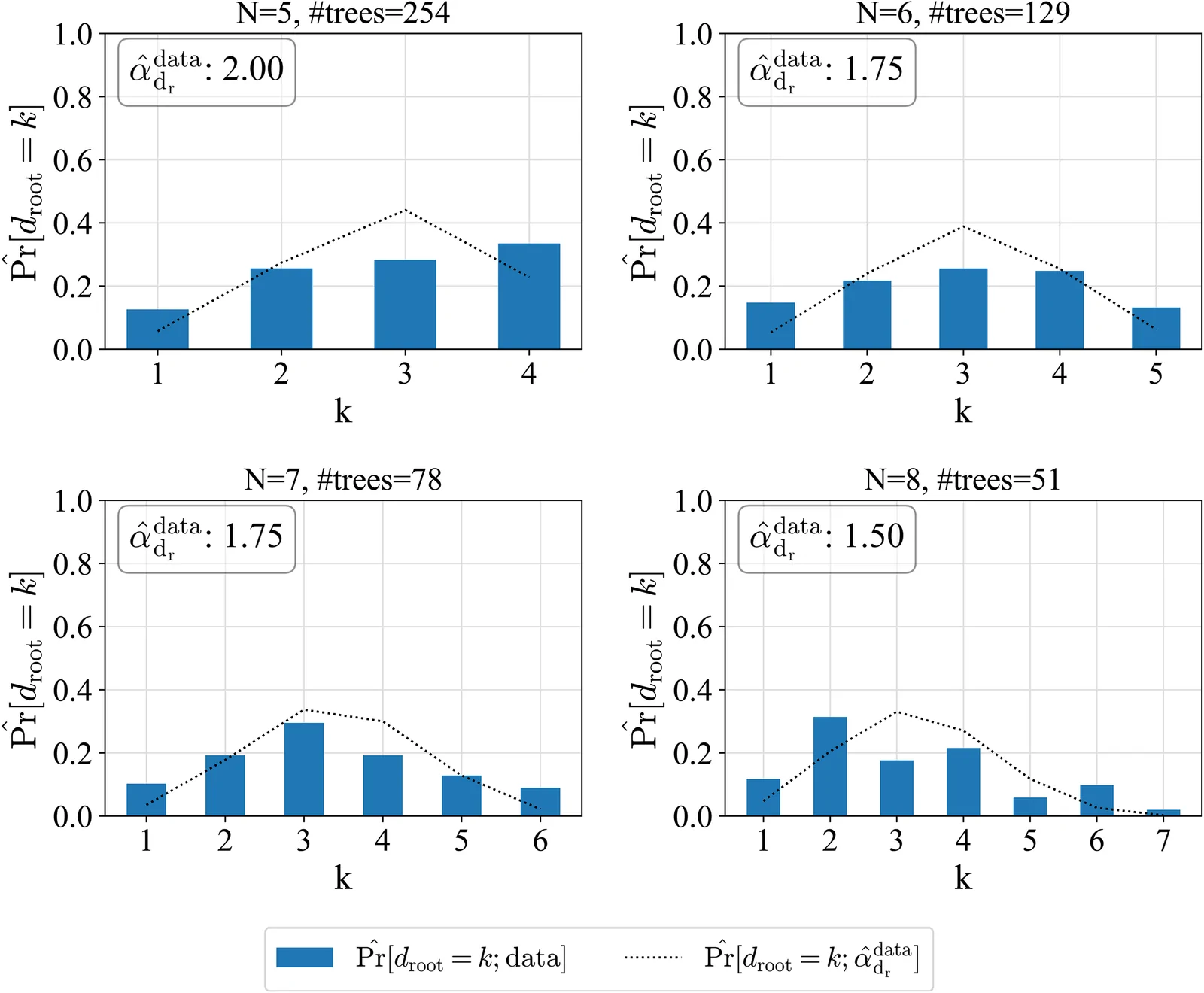
Empirical root outdegree distribution obtained from the set of infection trees of size *N* = 5,6,7,8 belonging to wave 4. In black the root outdegree distribution obtained from the non-Markovian SI simulation on the complete graph with size *N* and $\alpha_{sim}$ equal to the maximum likelihood estimate ${\hat{\alpha }}_{d_{r}}^{data}$ in the range [0.5,4.5] (with steps of 0.25) given the empirical distribution. In blue the empirical root outdegree distribution.

The second method to measure the non-Markovian parameter α (Fig 5) does not use the approximation in (7), but only relies on the root outdegree distribution from the SI process simulations (S4 Fig). Fig 7 shows the empirical distribution for wave 4 and tree sizes *N* = 5,6,7,8. The black dotted line is the root outdegree distribution obtained from simulations with $\alpha_{sim}$ equal to the maximum likelihood estimate ${\hat{\alpha }}_{d_{r}}^{data}$ of the shape parameter, given the root outdegree distribution of data (in blue).

**Fig 8:**
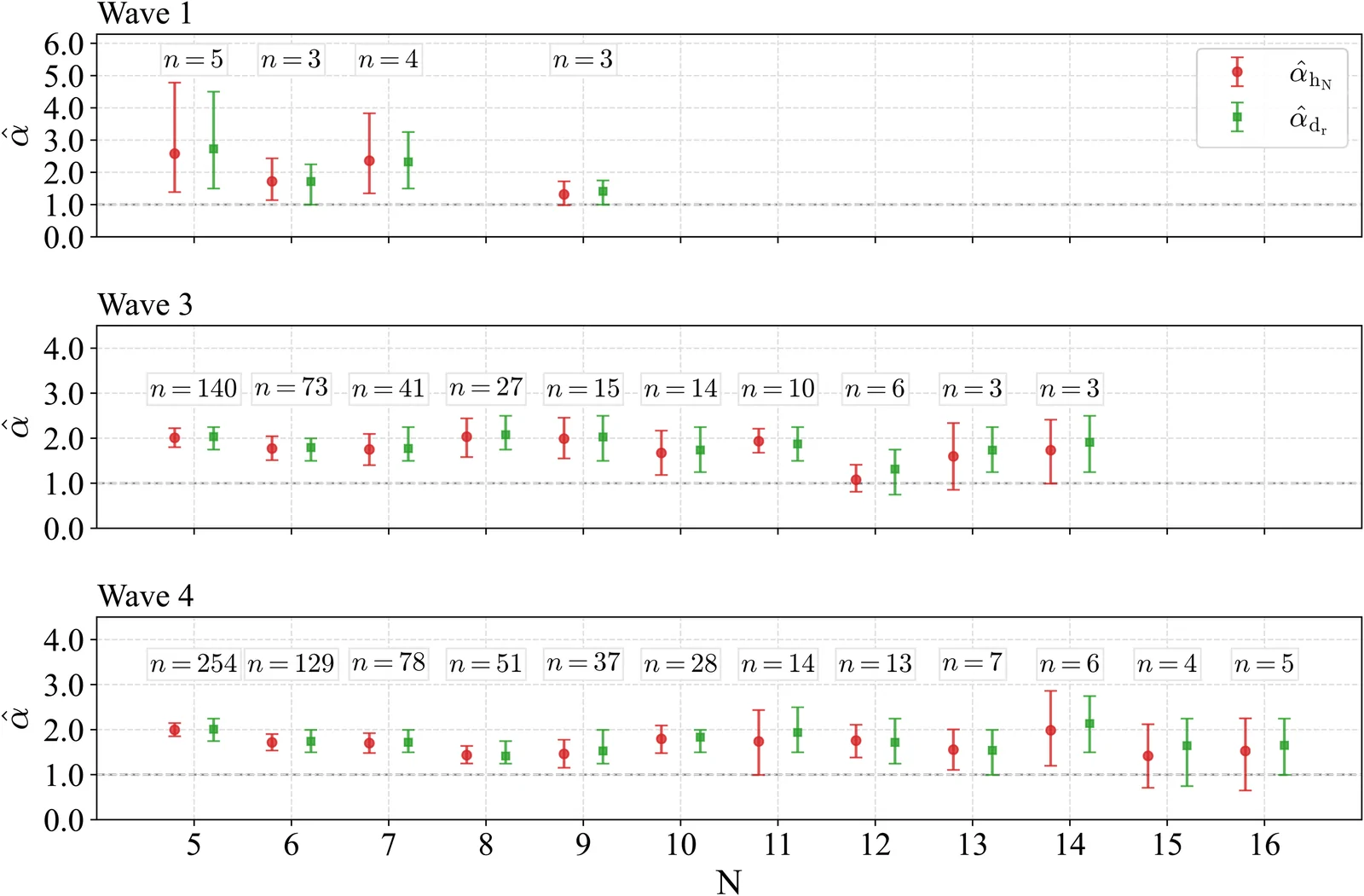
Summary of all the parameters α obtained on the sets of infection trees of wave 1, 3 and 4. In red the estimates of the shape parameter α employing the method based on the hopcount distribution of the infection trees (${\hat{\alpha }}_{h_{N}}$). In green, the estimates of the shape parameter α employing the method based on the root outdegree distribution of the infection trees (${\hat{\alpha }}_{d_{r}}$). The error bars represent the 90% confidence intervals given by the bootstrap procedure performed resampling 1000 times with replacement each set of trees of the same size. The number *n* on top of each estimate indicates how many infection trees are employed for a given size *N*.

In Fig 8, we show the estimate of the non-Markovianity parameter α for all the available trees with *N* > 4, $n_{trees}>3$ and for all the waves. The threshold of at least three independent chains per tree size was imposed to ensure meaningful statistical comparison (see also S5 Fig). Wave 2 is excluded from Fig 8 because it represented a relatively small epidemic wave and did not contain a sufficient number of infection trees of each size to meet this minimum sample requirement.

The error bars show the limits of the 90% confidence intervals devised with the bootstrap resampling. Fig 8 demonstrates that the average values of the estimate of α are always greater than 1 regardless of the tree size and of the number of trees in the dataset. The confidence intervals are clearly dependent on the size of the single tree set and increase when we have a small number of trees in a set. In the cases in which we have a larger number of trees, both the methods give a value of α greater than 1 with 95% statistical significance. Due to the limited size of the specific tree sets, the points for which the Markovian hypothesis cannot be rejected (for both methods) with 95% statistical significance are: wave 1 tree size *N* = 9; wave 3 tree sizes *N* = 12; wave 4 tree sizes *N* = 15, 16. However, the average value of α is bigger than one also in these cases. Finally, the values of our non-Markovianity parameter α seem to always be around 1.5-2, regardless of the tree size or wave, meaning that the non-Markovian model we have employed uncovers some *universal* properties of the measured spreading process. The values of α bigger than 1 are also in line with previous evidence from COVID-19 literature [17,18], which suggest that infection times can be fitted (see also S6 Fig) with a Weibull distribution with shape parameter bigger than one [37].

To verify whether the variability in the confidence intervals width stems from the methods itself or from the data, we performed a comparative analysis of both methods on synthetic data (S5 Fig). Our synthetic analysis demonstrates that both methods are remarkably robust and with comparable statistical power. The confidence intervals observed in Fig 8 are wider than the one obtained on synthetic data (S5 Fig), because they reflect the natural noise and variance present in real-world contact tracing data. Hence, the “empirical” confidence intervals provide an upper bound which allows us to safely reject the Markovian hypothesis whenever the 5th percentiles of the estimates in Fig 8 are above α=1.

### Effective reproduction number

From the outdegree distribution of the infection trees in different time windows, we can make an estimate of the expected number of secondary cases produced by an individual tested positive in that specific time period. We employ the definition of the effective reproduction number $\hat{R}(t;\Delta t)$ within the time window (t−Δt,t] presented in Sec “Effective reproduction number from the evolution in time of the outdegree distribution” to examine the evolution of the COVID-19 epidemic in Cyprus. We then compare our effective reproduction number $\hat{R}(t;\Delta t)$ with previous measures of the reproduction number for the same epidemic in Cyprus [42,44].

**Fig 9:**
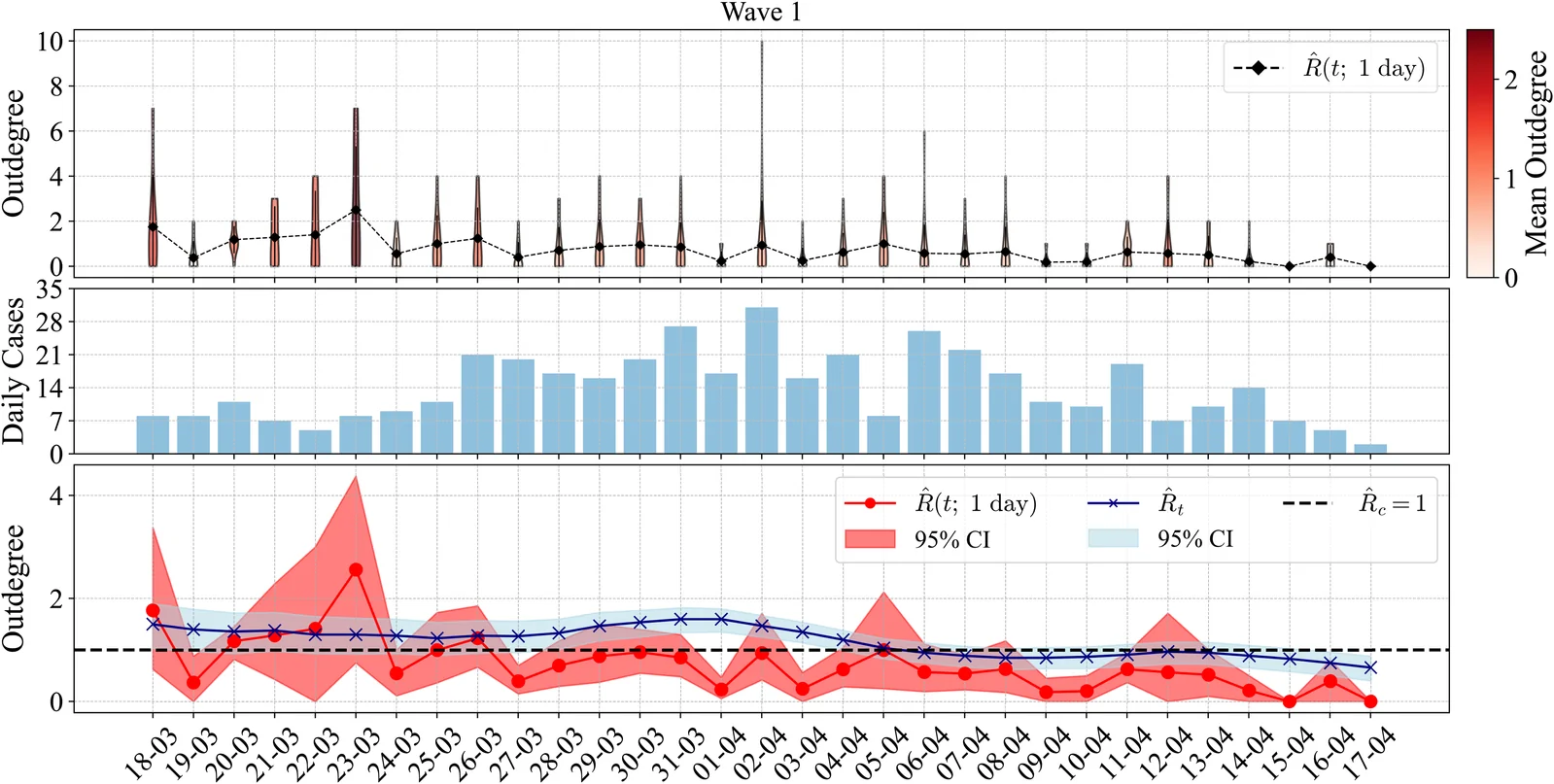
Daily evolution of the first wave of the COVID-19 epidemic in Cyprus. Daily out-degree distribution (top) and daily reported case counts (middle) during the first COVID-19 wave in Cyprus. Violin plots represent the distribution of secondary infections per case per day; color intensity indicates the mean outdegree. (Bottom) Evolution of the effective reproduction number $\hat{R}(t;1\;day)$ in the first wave of the COVID-19 epidemic in Cyprus. The red line shows the mean outdegree for each day derived from bootstrapped samples, with the 95% confidence intervals indicated by the red area. The blue line represents the effective reproduction number reported in [44]. In dashed black the reference line at the critical value ${\hat{R}}_{c}=1$.

**Fig 10:**
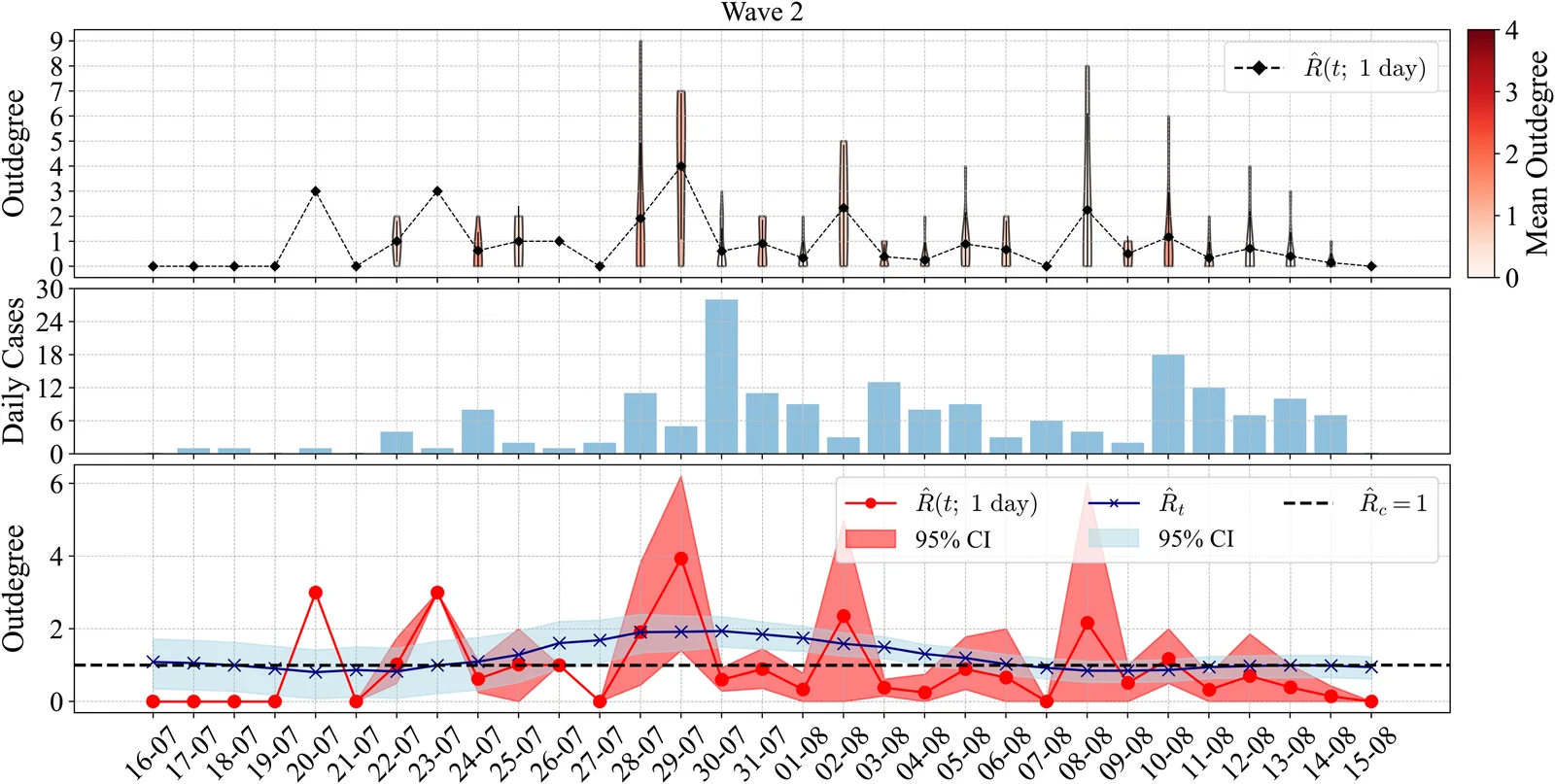
Daily evolution of the second wave of the COVID-19 epidemic in Cyprus. Daily out-degree distribution (top) and daily reported case counts (middle) during the second COVID-19 wave in Cyprus. Violin plots represent the distribution of secondary infections per case per day; color intensity indicates the mean outdegree. (Bottom) Evolution of the effective reproduction number $\hat{R}(t;1\;day)$ in the first wave of the COVID-19 epidemic in Cyprus. The red line shows the mean outdegree for each day derived from bootstrapped samples, with the 95% confidence intervals indicated by the red area. The blue line represents the effective reproduction number reported in [44]. In dashed black the reference line at the critical value ${\hat{R}}_{c}=1$.

Figs 9 and 10 display the number of confirmed cases of SARS-CoV-2 transmission during the first two epidemic waves in Cyprus, while S9 and S10 Figs during the third and fourth waves. The upper panels show violin plots of the daily outdegree distribution for the cases tested positive on that single day. The width of each violin reflects the variability in transmission, while the color intensity corresponds to the mean outdegree $\hat{R}(t;1\;day)$ for that day. Lighter colors indicate lower average secondary transmissions, and darker shades indicate more intense transmission clusters. The central panels display the corresponding number of daily reported cases during the same period. Peaks in the number of daily cases often correspond to broader outdegree distributions with higher average, suggesting super-spreading events or days with higher transmission heterogeneity. In the bottom panel of Fig 9, we compare our effective reproduction number $\hat{R}(t;1\;day)$ for the first wave with the reproduction number ${\hat{R}}_{t}$ reported in [42]. The estimate in [42] was derived using a meta-population model proposed by Peng et al. [45], a generalization of the classical SEIR (susceptible-exposed-infected-recovered) model. For each day, we apply a bootstrap resampling method to generate a distribution of outdegree values, from which we calculate the mean and the 95% confidence intervals. The red points in the figure represent the bootstrapped means, with the red area indicating the 95% confidence intervals. The blue line shows the effective reproduction numbers from [42], along with their own confidence intervals. For wave 1 we observe that for 28 out of the 31 days the value of $\hat{R}(t;1\;day)$ is lower than the ${\hat{R}}_{t}$ computed in [42]. Our estimate $\hat{R}(t;1\;day)$ becomes higher than ${\hat{R}}_{t}$ when the daily outdegree distribution has a very large variance reflecting the presence of superspreaders which are difficult to detect with ordinary compartmental models [24].

Similarly, for wave 2 we compare our estimates with the results from the report of the Cyprus Ministry of Health [44]. The methods employed by the Cyprus MoH in [44] to compute the effective reproduction number are the same as the one in [42]. Fig 10 shows that our estimate $\hat{R}(t;1\;day)$ is lower on 23 out of the 31 days of wave 2, while it is higher when there is a higher variance on the daily outdegree distribution.

**Fig 11:**
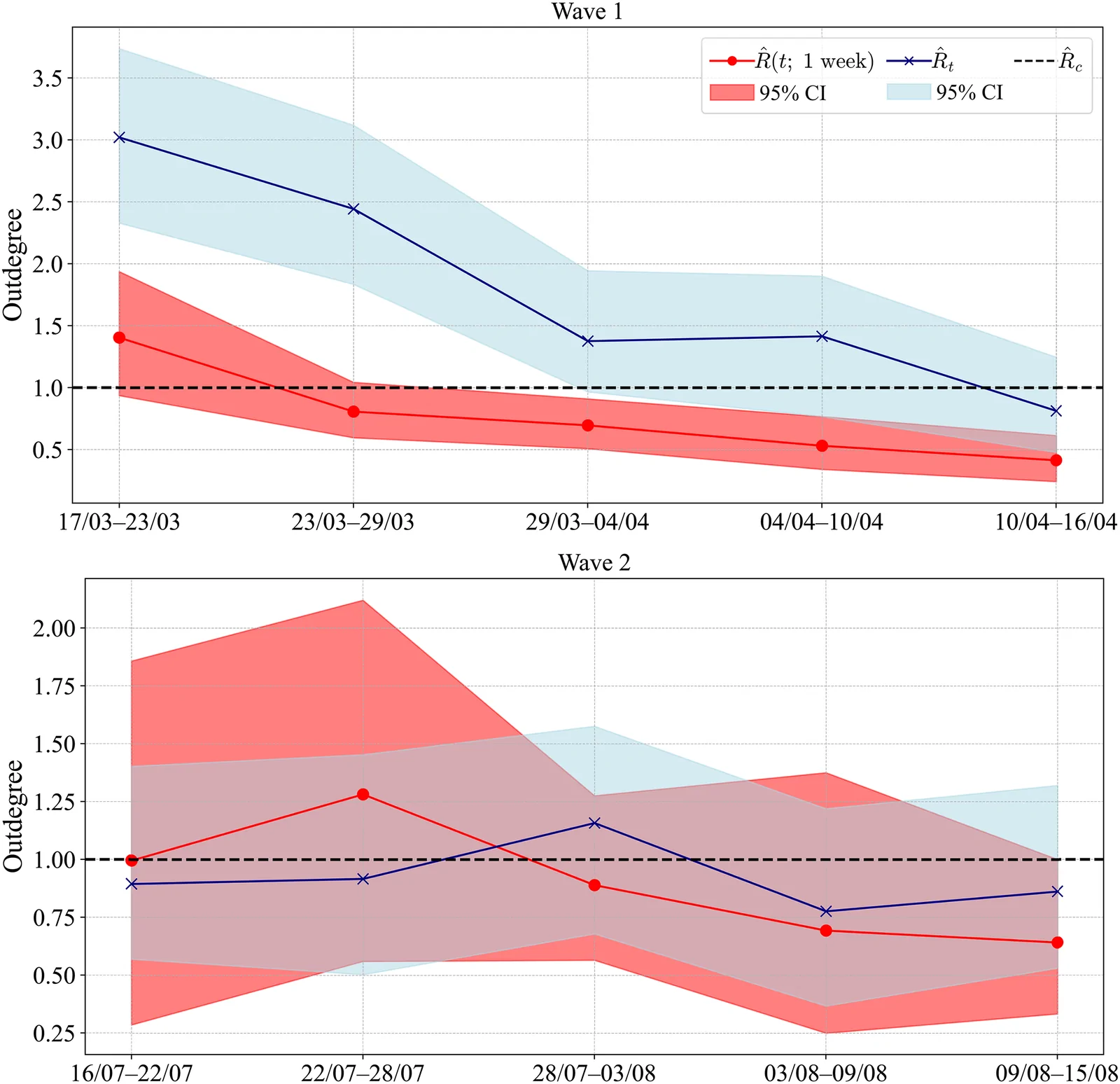
Evolution of the effective reproduction number $\hat{R}(t;1\;week)$ in the first two waves of the COVID-19 epidemic in Cyprus. The red line shows the mean outdegree for each week derived from bootstrapped samples, with the 95% confidence intervals indicated as a red area. The blue line for Wave 1 represents the effective reproduction number reported in Fig 10 of [42]. The blue line for Wave 2 displays values from [44]. In dashed black the reference line at the critical value ${\hat{R}}_{c}=1$.

Successively, we compare our measure $\hat{R}(t;1\;week)$ for the first and second wave with the reproduction number ${\hat{R}}_{t}$ computed in [42,44] employing a meta-population model of Li et al. [41] incorporating information on human movement between the five main districts of Cyprus. The comparison is displayed in Fig 11. We observe that for wave 1 the value of $\hat{R}(t;1\;week)$ is always lower than the ${\hat{R}}_{t}$ computed in [42] and follows the same trend. For wave 2 the reproduction number from [44] always falls in our confidence interval showing a good agreement with our estimate.

For waves 3 and 4, as shown in S9 Fig and S10 Fig, it is even more evident that our effective daily reproduction number $\hat{R}(t;1\;day)$, estimated from the outdegree distribution of the infection trees, consistently falls below the effective reproduction number computed with compartmental models and data from [42].

We thus conclude that our estimate $\hat{R}(t;\Delta t)$ of the effective reproduction number often lower bounds measures obtained with compartmental or meta-population models [42,44]. Indeed, in the computation of the effective reproduction number $\hat{R}(t;\Delta t)$ we include all trees with N≥2 nodes, which are probably partial subtrees of larger infection trees. In small trees the majority of the nodes exhibit an average outdegree smaller than one, thereby reducing the average number of secondary cases.

Finally, a high variance of the outdegree distribution indicates the presence of nodes from larger trees. In such cases, our effective reproduction number $\hat{R}(t;\Delta t)$ increases, and leads to an overestimation of the reproduction number computed in [42,44]: nodes with very high outdegree (sometimes called superspreaders [24]) are often overlooked by mean field compartmental models.

### A simple pruning technique to mitigate the underestimation of the effective reproduction number

**Fig 12:**
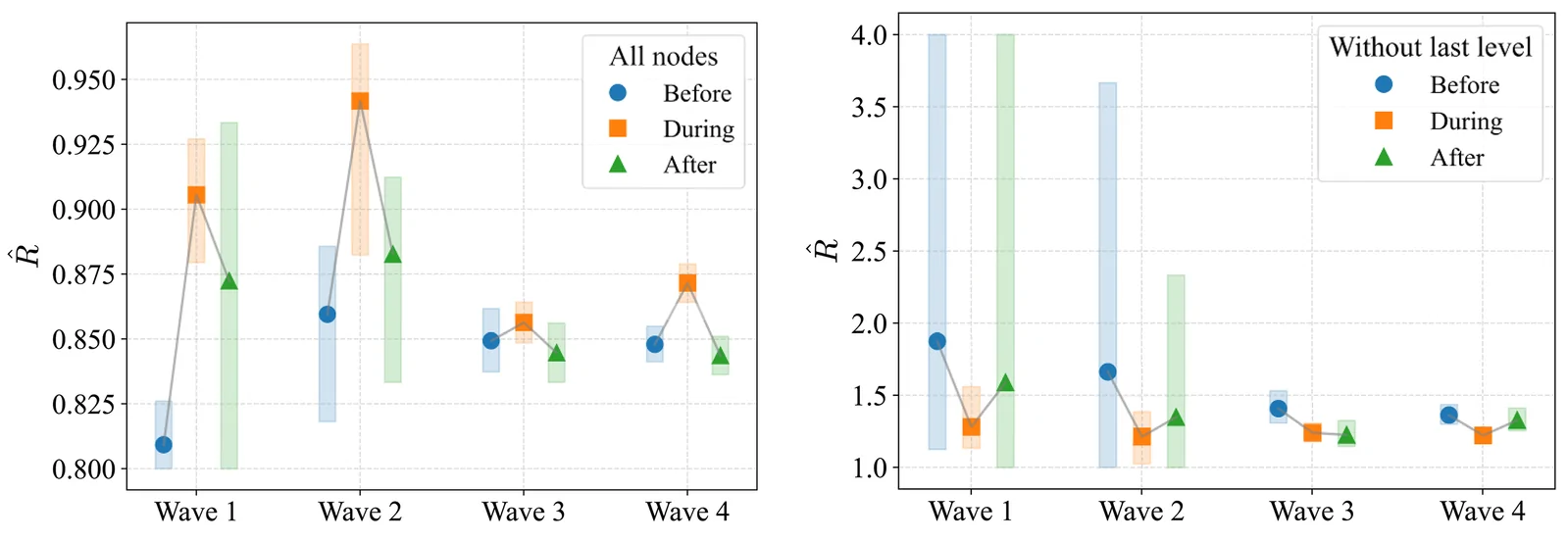
Average outdegree and 95% confidence intervals for the infection trees belonging to different periods around the peak of the four COVID-19 waves in Cyprus. The trees are divided into three groups: trees whose nodes were only tested positive before the peak (Before), trees whose nodes were tested positive before and after the peak (During), and trees whose nodes were only tested positive after the peak (After). On the left the plot when no pruning is applied. On the right the estimate where the last level of the infection trees is pruned.

We conclude proposing a basic procedure to mitigate the bias introduced by the children nodes in the trees: we simply prune the last level of the infection trees and we compute again $\hat{R}(t;\Delta t)$ in different time windows. Fig 12 presents $\hat{R}(t;\Delta t)$ computed in three different time windows before, after or during the peak of the wave (maximum number of confirmed daily cases). On the left the effective reproduction number has been computed retaining the full structure of the trees, on the right removing the last level of any tree. The trees are divided into three groups: trees whose nodes were tested positive only before the peak (Before), trees whose nodes were tested positive before and after the peak (During), and trees whose nodes were only tested positive after the peak (After).

From the results in Fig 12, the estimate without the last level (right figure) is the one which reasonably reflects the expected evolution of the epidemic wave. Without the last level we observe a decrease in the effective reproduction number from before to after the peak of each wave. Retaining all the nodes shows instead smaller relative variations before and after the peak or even an increase in the effective reproduction number, a behavior that is inconsistent with the observed epidemic trajectory. Similar conclusions can be drawn from S11 Fig for the daily effective reproduction number $\hat{R}(t;1\;day)$. The infection trees without the last level provide a more conservative estimate of the effective reproduction number, avoiding the underestimation introduced by leaf nodes.

## Discussion

In this work, we extract from the topology of real infection trees a non-Markovianity measure α, which is the shape parameter of a Weibull (6) distribution. The hopcount distribution alone reveals that infection trees of varying sizes can be described as realizations of a spreading process with non-exponential infection times characterized by a Weibull distribution with shape parameter α>1. Fig 8 illustrates the results for different waves and different tree sizes, highlighting the general non-Markovianity of the underlying spreading process. In our model, the infection time acts as an *effective transmission time* that inherently captures multiple, often unobservable, delays, ranging from true biological latency to human behavioral factors. Additionally, our non-exponential infection time corresponds to an effective non-exponential generation interval [37], in line with previous evidence from COVID-19 [17,18].

Generalized epidemic models [46] can be created by extending the basic SI process with additional compartments (e.g., recovered, exposed) and are hence able to mimic non-exponential infection times via a sum of independent exponential delays. Although such generalized models may provide a valid statistical description of the data (S7 Fig and S1 Table), the underlying Markovian assumptions can lead to fitting parameters with values that contradict established timelines measured in COVID-19 (e.g., incubation rate much larger than the transmission rate S1 Table). Furthermore, generalized epidemic models require additional parameters that are quite difficult to measure, in contrast to our phenomenological approach, that systematically absorbs all the complexity into a single parameter α. Our approach is further validated when a recovery compartment is introduced: S8 Fig shows that a non-Markovian SIR process still necessitates an α>1 to match the empirical data, confirming that the deviations from the Markovianity are not an artifact of our SI modeling assumption. The well-established Markovian case α=1, that produces nearly all currently used mean-field epidemic models, may thus provide a safe upper bound for the nodal infection probabilities in the contact graph and an upper bound for the basic reproduction number [37].

Our computations are based on the assumption that each observed infection tree of size *N* is an independent realization of a non-Markovian SI process on the complete graph $K_{N}$. The assumption of independence between the infection trees is justifiable in large populations, in particular when the global contact network is unknown. By treating the trees as separate and independent, we neglect possible, but hard-to-measure dependencies between trees. The resulting estimate of α per tree size is derived from localized transmission events.

Each infection process in the network is assumed to be independent from the others and the infection time is Weibull distributed with shape parameter α and rate parameter β=1. The choice of the value for the rate parameter β does not affect the SI process [28, Sec. 16.3]: when the infection times are i.i.d random variables, a global rescaling of all link infection times does not change the infection tree structure.

The observed infection trees are usually small and do not contain information about the curings, which makes SI modeling meaningful. The complete graph assumption and the absence of curing processes overestimate the viral force of a single node to infect its neighbors. Adding recoveries in the SI process often requires a higher value of α to obtain a hopcount distribution closer to the empirical one, as shown in S8 Fig, meaning that our estimates of the non-Markovian parameter α are safely larger than 1.

Although contact tracing investigations in Cyprus were conducted thoroughly, it was nearly impossible to trace all infections and construct a single connected infection tree per each wave. As a result, the observed infection trees are sparse and affected by under‑reporting or incomplete case detection [7,33,47,48]. Such limitations may directly affect our estimates: if branches from the root node are missing, the computed α value is underestimated, while if branches are missing from the leaf nodes, α is overestimated. Nonetheless, the key qualitative conclusion α>1 is observed consistently across different waves and multiple tree sizes.

Our estimates of the reproduction number tend to produce lower values compared to previous studies [42,44], except when the daily outdegree distribution variance is very large. To mitigate the effect of the leaf nodes, a simple pruning that removes the last level of the infection trees results in an effective reproduction number estimate which follows more accurately the evolution of the epidemic, as shown in Fig 12.

## Conclusion

We show that real-world epidemics are often characterized by non-exponential infection processes and, consequently, by non-Markovian dynamics. In this study, we quantify the non-Markovianity by the parameter α. Empirical data consistently points to a value of α>1, which indicates that the infection process likely exhibits non-Markovian characteristics. Our study statistically rejects the Markovian hypothesis (α = 1), based solely on the topology of real contact-tracing data.

Additionally, the analysis of infection trees offers an independent and complementary approach for estimating the effective reproduction number, a key epidemiological metric. Finally, our study demonstrates the value of “infection tree construction” and stimulates the design of digital apps that are able to automatically reconstruct the underlying transmission network [49,50].

## Supporting information

S1 File Supplementary material, which includes S1-S5 Appendixes, S1-S11 Figs, and S1 Table. (PDF)
