# Supplementary material for: Non-Markovian dynamics and effective reproduction number in COVID-19: Evidence from Cyprus contact tracing data
Source: PLoS Comput Biol. 2026 Aug 21;22(8):e1014578. doi: 10.1371/journal.pcbi.1014578 (PMC13496441; doi:10.1371/journal.pcbi.1014578)
Supplement: Supplementary file 1 [file pcbi.1014578.s001.pdf]

Supporting Information for "Non-Markovian dynamics and effective reproduction number in COVID-19: evidence from Cyprus contact tracing data"

Pavlos Alexandros Dimitriou[1,2], Matteo D'Alessandro[3*], Brian L. Chang[3], Valentinos Silvestros[4], Elisavet Constantinou[4], Costas Pitris[1,2], Panayiotis Kolios[1,5] and Piet Van Mieghem[3]

1 University of Cyprus, Department of Electrical and Computer Engineering, Nicosia, Cyprus

2 KIOS Research and Innovation Center of Excellence, Nicosia, Cyprus

3 Delft University of Technology, Faculty of Electrical Engineering, Mathematics and Computer Science, Delft, The Netherlands

4 Ministry of Health, Nicosia, Cyprus

5 University of Cyprus, Department of Computer Science, Nicosia, Cyprus

*m.d.dalessandro@tudelft.nl

S1 Additional information on the contact tracing data

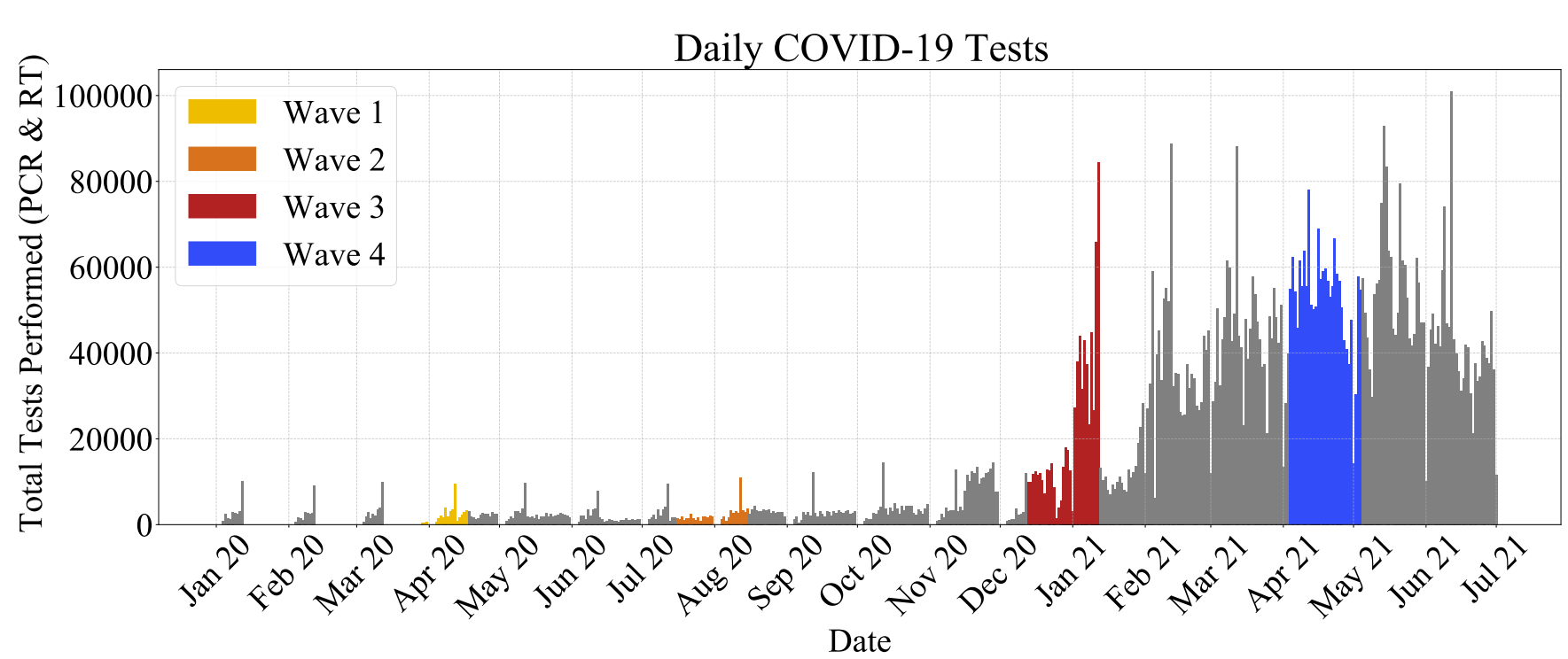

S1 Fig. Number of daily tests over the four waves of the COVID-19 epidemic in Cyprus.

The level of restrictions is measured according to the so called “stringency index” [1] that is calculated based on nine metrics: school closures, workplace closures, cancellation of public events, restrictions on public gatherings, closures of public transport, stay-at-home requirements, public information campaigns, restrictions on internal movement, and international travel controls. The stringency index ranges from 0 to 100. S2 Fig shows the changes of the stringency index during the 31 days of the 4 waves of the COVID-19 in Cyprus. During the first wave, strict measures were in place, with an average stringency index of 85.18. These measures included the closure of most retail services and schools, the suspension of incoming flights, and restrictions on internal movement. During the second wave, the mean stringency index was reduced to 47.22, with fewer restrictions. In the third wave, it increased to 74.28, with some restrictions on internal movement and gatherings. By the fourth wave, the average stringency index was around 65.50 [2].

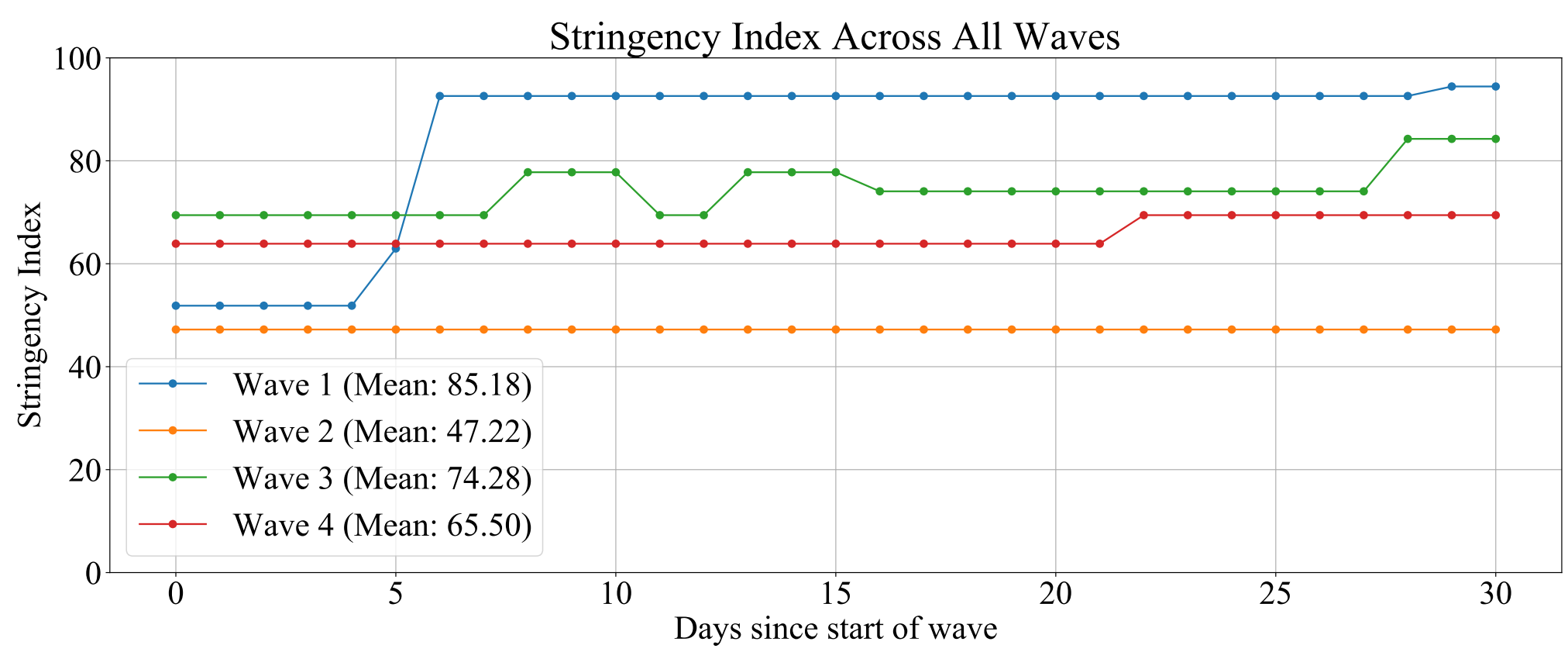

S2 Fig. Stringency index across the 31 selected days of the four COVID-19 epidemic waves in Cyprus.

S2 SI process on graphs

The state of a node i at time t in a Markovian SI process on a graph is specified by a Bernoulli random variable $X_i\,(t) \in \{0,1\}$: $X_i\,(t) = 0$ for a susceptible node and $X_i\,(t) = 1$ for an infected node. A node i at time t can be in one of the two states: *infected*, with probability $w_i(t) = \Pr[X_i(t) = 1]$ or *susceptible* with probability $1 - w_i(t)$. We assume that the infection process per link is a Poisson process with rate β. Only if a node is infected, then it can infect its direct neighbors, that are still susceptible. All the

infection Poisson processes are independent. This is the general continuous-time description of the simplest type of a Susceptible-Infected (SI) process on a network.

A description of the SI epidemic process on a graph is as follows. Let I denote the set of infected nodes in the graph G and let a_{ij} be an element of the adjacency matrix A. Then, the Markov transitions

$$\text{for } j \notin I\text{:} \quad I \mapsto I \cup \{j\} \quad \text{ at rate } \beta \textstyle\sum_{k \in I} a_{kj}$$

detail the dynamics between the infected subgraph I and its complement $I^c = G \backslash I$. A susceptible node can be infected by any of its infected, direct neighbors, where each neighbor acts independently of the others. If each neighbor $k \in \mathcal{N}(i)$ of node i, where $\mathcal{N}(i)$ is the set of neighbors of node i, has rate β, then the node i is infected by that neighbor that transmits the infection the fastest. This means that the time at which the infection event at node i occurs, is the minimum of the infection times $T_{j \to i}$ from each infected neighbor j. The minimum of independent, exponentially distributed times each with rate β is again an exponential random variable with rate $\sum_{k \in \mathcal{N}(i)} \beta$, a property that is further physically explained in a Markov discovery process [3, Section 16.2] that allows us to model an SI process as a stochastic shortest path problem on a graph.

S2.1 Asymptotic non-Markovian hopcount distribution

In the shortest path, the tail of the link distribution is less important [3, Sec. 16.1] and therefore their distribution can be approximated as $1 - e^{-x^\alpha} \simeq x^\alpha$ for small x. Upper bounding then $\Pr[h_N \le k, w_N \le z]$ with the Boole's inequality ($\Pr[\cup_j A_j] \le \sum_j \Pr[A_j]$), and subsequently inferring an asymptotic expression for $\Pr[h_N = k]$ given the choice of a typical value z for the weight w_N of the shortest path between any two nodes in the complete graph K_N, gives an asymptotic formula [4, Sec. 6.1] for the hopcount distribution:

$$\Pr[h_N = k; \alpha] \approx \frac{1}{N} \frac{(\ln \alpha N)^{\alpha k}}{\Gamma(\alpha k + 1)}. \tag{S1}$$

A more rigorous proof for the hopcount distribution of the shortest path tree in the complete graph with i.i.d link weights distributed as $X^{1/\alpha}$ can be found in [5].

S2.2 Root outdegree distribution in non-Markovian SI infection trees

The outdegree distribution of the root of an SI infection tree on the complete graph K_N, with homogeneous and independently drawn infection times, is related to the probability that the hopcount of a randomly selected node is equal to one. In general, the average of the outdegree distribution equals $E[d_{\text{root}}] = N\Pr[h_N = 1]$.

Markovian case In the Markovian case, the URT structure can be exploited to derive the exact root outdegree distribution [3, Eq. (16.46)]

$$\Pr[d_{\text{root}} = k; \alpha = 1] = \frac{(-1)^{N-1-k} S_{N-1}^{(k)}}{(N-1)!}. \tag{S2}$$

Non-Markovian case In the non-Markovian case instead, the exact calculations are again complex, because the probability that the root infects a new node depends on the whole history of the process. Nevertheless, we can prove the following theorem for Weibull residual random variables, which gives an intuitive relation between the outdegree of the root and the shape parameter α of the (Weibull) infection time distribution.

Theorem 1. *Given the two Weibull residual time random variables $R_{u_1}^{(\alpha)}$ and $R_{u_2}^{(\alpha)}$ distributed according to*

$$Pr[R_{u_i}^{(\alpha)} \geq t] = \frac{e^{-(\beta(t+u_i))^\alpha}}{e^{-(\beta u_i)^\alpha}}, \tag{S3}$$

the following inequalities hold:

1. *If $\alpha > 1$ and $u_1 > u_2$*

$$\Pr[R_{u_1}^{(\alpha)} \leq t] > Pr[R_{u_2}^{(\alpha)} \leq t], \quad \forall t \in (0, \infty) \tag{S4}$$

2. *If $\alpha < 1$ and $u_1 > u_2$*

$$\Pr[R_{u_1}^{(\alpha)} \leq t] < Pr[R_{u_2}^{(\alpha)} \leq t], \quad \forall t \in (0, \infty) \tag{S5}$$

Proof of 1. With (S3), (S4) becomes:

$$1 - \frac{e^{-(\beta(t+u_1))^\alpha}}{e^{-(\beta u_1)^\alpha}} > 1 - \frac{e^{-(\beta(t+u_2))^\alpha}}{e^{-(\beta u_2)^\alpha}},$$

simplifying and reversing the inequality we obtain:

$$e^{-(\beta(t+u_1))^\alpha + (\beta u_1)^\alpha} < e^{-(\beta(t+u_2))^\alpha + (\beta u_2)^\alpha},$$

Applying the logarithm on both sides of the inequality as it is a monotonically increasing function and reversing again the inequality because of the minus sign on both sides we write:

$$(\beta(t+u_1))^\alpha - (\beta u_1)^\alpha > (\beta(t+u_2))^\alpha - (\beta u_2)^\alpha. \tag{S6}$$

The left- and the right-hand side of (S6) are two monotonically increasing functions of $t \in [0, +\infty)$ which, for $t = 0$, are both 0. Given that $u_1 > u_2$, we just need to prove that left-hand side of (S6) increases faster than the right-hand side in order for (S6) to be true. We therefore differentiate both sides of (S6) in t to obtain

$$\frac{\partial}{\partial t}[(\beta(t+u_1))^\alpha - (\beta u_1)^\alpha] = \alpha\beta^\alpha(t+u_1)^{\alpha-1} >$$
$$\frac{\partial}{\partial t}[(\beta(t+u_2))^\alpha - (\beta u_2)^\alpha] = \alpha\beta^\alpha(t+u_2)^{\alpha-1},$$

from which

$$(t+u_1)^{\alpha-1} > (t+u_2)^{\alpha-1}. \tag{S7}$$

Given that $\alpha > 1$ and $u_1 > u_2$ (S7) is always true for any $t \in (0, +\infty)$ and therefore (S4) is proven.

□

Proof of 2. We can employ the same steps as in the *Proof of 1* with the only difference that (S7) is reversed because $\alpha < 1$ in this case. From the reversed inequality relation (S5) follows directly. □

When $\alpha = 1$, the memoryless property of the exponential function shows that all the residual variables are exponentially distributed (see Sec. Markovian SI infection tree and shortest path tree with exponential link weights).

Theorem 1 implies that an infection tree of a non-Markovian SI process with homogeneous Weibull distributed infection times on the complete graph will be characterized, compared to the Markovian case, by a smaller depth and a higher root degree for $\alpha > 1$, a greater depth and a lower root degree for $\alpha < 1$. Indeed, the residual random variable $R_u^{(\alpha)}$, which describes the infection time between two nodes given that a time u has already passed, is characterized by a faster than exponential decaying density function for $\alpha > 1$, which favors very small infection times. Instead, a very slow decaying density function appears for $\alpha < 1$, which favors very large infection times. It follows that the average of the root outdegree distribution, for N sufficiently large, increases with α in the non-Markovian case, as we can also observe employing the approximation in equation (S1), which translates to $E[d_{\text{root}}] \simeq \frac{(\ln \alpha N)^{\alpha}}{\Gamma(\alpha+1)}$. Similar properties are already highlighted in [6, 7].

S3 Simulation of the SI infection trees as shortest path trees

The spread of an SI epidemic process on a graph G originating from a single initially infected root node can be mapped exactly to a shortest path problem. The time for the infection to transmit across a link (i, j) is represented by the random infection time $T_{i \to j}$, which acts as the weight of the directed link from node i to node j. The time at which any susceptible node in the network becomes infected is equivalent to the length of the shortest path from the root node to that node, with the path length defined as the sum of the infection times along the links of the shortest path. The shortest path tree, which is the union of all shortest paths from the root to any other node, corresponds directly to the infection tree of the epidemic SI process.

For a classic Markovian SI process (S2 Appendix), the transmission times $T_{i \to j}$ are exponentially distributed, reflecting a memoryless Poisson process with a constant infection rate β. In contrast, our non-Markovian formulation allows $T_{i \to j}$ to follow different probability distributions, such as the Weibull distribution with a shape parameter α and rate parameter β. Simulating the infection spread and extracting the expected hopcount distributions therefore reduces to sampling the link weights $T_{i \to j}$

from the chosen distribution and applying Dijkstra's algorithm to compute the shortest path tree [8].

This shortest-path analogy naturally extends to more complex compartmental models, such as those including exposed (SEI) or recovered (SIR) states:

- **SEI processes (Incubation):** The incubation time T_i^{inc} (or latent period) of node i is the delay of node i between receiving the infection and being infectious. The effective transmission time from node i to a neighbor j becomes the sum of the incubation time T_i^{inc} and the infection time $T_{i\to j}$, and equivalently, the link weights are modified to $T_{i\to j} + T_i^{\text{inc}}$.

- **SIR processes (Recovery):** A recovery period can be modeled by assigning a random recovery time T_i^{rec} to each node i. An infected node i can only transmit the disease to its neighbor j if the infection occurs before recovery ($T_{i\to j} < T_i^{\text{rec}}$). If the sampled transmission time exceeds the recovery time, the transmission does not occur, which is computationally equivalent to removing the link (i, j) from the graph prior to running the shortest path algorithm.

S4 Methods in detail

S4.1 Estimate of non-Markovian dynamics from hopcount distribution

In the following, we detail the method to extract α from the hopcount distribution of data in a statistical significant way.

Part 1: simulations

1. We select a wave in the dataset and we extract the number of trees n_{trees} for any given tree size N. We exclude the trees with size $N < 5$ and for which $n_{\text{trees}} < 3$.

2. For each tree size N we perform n_{trees} Monte Carlo simulations of a non-Markovian SI process on the complete graph with N nodes varying the Weibull parameter α_{sim} in $[0.5, 4.5]$, with steps of 0.25. Each simulation gives n_{trees} trees at fixed N and at a given α_{sim}.

3. For each set of n_{trees} simulated trees at fixed α_{sim} we compute the hopcount distribution as the average number of nodes that are distant k hops from the initially infected root, divided by N:

$$\hat{\Pr}[h_N = k; \alpha_{\text{sim}}] = \frac{1}{N} \frac{1}{n_{\text{trees}}} \sum_{i=1}^{n_{\text{trees}}} v_i, \tag{S8}$$

where v_i is the number of nodes at distance k (in terms of hop) from the root in the i-th simulated tree and $k = 0, ..., N-1$.

4. We fit each empirical distribution (S8) to the approximated expression given by (S1), using the least squares method. From this fit, we estimate the parameter $\hat{\alpha}_{\text{LS}}$.

5. For each (α_{sim}, N) we repeat step 2, 3 and 4 a number $N_{\text{bs}} = 1000$ times and we compute the mean, the 5th and the 95th percentiles of the distribution of the estimated $\hat{\alpha}_{\text{LS}}$. For each value of α_{sim} in the range selected in step 2, we obtain three values characterizing the distribution of the least square estimate $(\hat{\alpha}_{\text{LS5}}, \hat{\alpha}_{\text{LS}}, \hat{\alpha}_{\text{LS95}})$ for a fixed tree size N and a fixed number of trees n_{trees}.

Part 2: data

1. Given the set of n_{trees} trees of size N in the dataset related to the selected wave, we build $N_{\text{bs}} = 1000$ datasets resampling with replacement the initial set of trees.

2. For each resampled dataset we compute the empirical hopcount distribution employing (S8), which in this case we define as $\hat{\Pr}[h_N^{(j)} = k; \text{data}]$, with $j = 1, ..., N_{\text{bs}}$.

3. We fit each bootstrapped empirical distribution $\hat{\Pr}[h_N^{(j)} = k; \text{data}]$ to the approximated expression given by (S1) using the least squares method. From this fit, we obtain the parameter defined $\hat{\alpha}_{\text{data}}^{(j)}$.

4. From the distribution of the $\hat{\alpha}_{\text{data}}^{(j)}$ extracted from the bootstrapped procedure, we compute the mean, the 5th and the 95th percentiles. For each tree size N in the dataset we obtain three values characterizing the distribution of the least square estimate $(\hat{\alpha}_{\text{data5}}, \hat{\alpha}_{\text{data}}, \hat{\alpha}_{\text{data95}})$.

5. To extract a final estimate for a given tree size N, we intersect $(\hat{\alpha}_{\mathrm{data5}}, \hat{\alpha}_{\mathrm{data}}, \hat{\alpha}_{\mathrm{data95}})$ with the line obtained from simulations which describes the relation between $\hat{\alpha}_{\mathrm{LS}}$ and α_{sim} at fixed N for n_{trees}. Using the intersection between the horizontal line for each of the three values $(\hat{\alpha}_{\mathrm{data5}}, \hat{\alpha}_{\mathrm{data}}, \hat{\alpha}_{\mathrm{data95}})$ and the relation α_{sim} vs $\hat{\alpha}_{\mathrm{LS}}$, approximated as a straight line around the intersection point, gives the corresponding values of α_{sim}that corresponds to the final estimate of α on the data. The confidence interval for the estimate is given by the α_{sim} values corresponding to the intersection point between the line α_{sim} vs $\hat{\alpha}_{\mathrm{LS}}$ and the two values α_{data5} and α_{data95}. If the extracted confidence interval is smaller than the one obtained from simulations, the latter is taken as a "safe" upper bound for the real confidence interval. The final estimate for each tree size N in the dataset will be denoted as $(\hat{\alpha}_{\mathrm{h_N}5}, \hat{\alpha}_{\mathrm{h_N}}, \hat{\alpha}_{\mathrm{h_N}95})$.

If after our procedure, the 5th percentile of the distribution of $\hat{\alpha}_{\mathrm{h_N}5}$ for a given tree size N is bigger than 1, with 95% confidence the α of our model is greater than 1 and the data shows non-Markovian properties.

S4.1.1 Correction of the estimates devised from equation (S1)

In order to correct the error introduced by the approximation in (S1), we compare the least squares estimates $\hat{\alpha}_{\mathrm{data}}$ performed on the empirical distribution with the least squares estimates $\hat{\alpha}_{\mathrm{LS}}$ extracted from the simulated hopcount distributions. We recall that for a given number of trees n_{trees} of size N in the selected wave, we simulate n_{tree} instances of a non-Markovian SI process on the complete graph of size N. The infection times are Weibull distributed with shape parameter α_{sim}. Each set of n_{tree} trees is generated 1000 times. For each set of simulated trees an hopcount distribution is computed from which a least square shape parameter is estimated. From the distribution of the estimates $\hat{\alpha}_{\mathrm{LS}}$ is extracted with confidence intervals.

S3 Fig shows the procedure in detail. In black the average and the percentiles related to the $\hat{\alpha}_{\mathrm{data}}$ estimated from the empirical hopcount distribution are shown. The blue line represents the average of the 1000 least squares estimates obtained from the fit of the simulated hopcount distribution. The green and the red lines are the percentiles of the same distribution. The intersection between the black lines and the blue line

corresponds to three values of $\alpha_{\rm sim}$, in orange in S3 Fig. These three values correspond to the values employed in simulations for which the least squares estimate of α, using $n_{\rm trees}$ and N fixed, gives the value $\hat{\alpha}_{\rm data}$ obtained on data. These three values are the final estimate of α and are denoted as $(\hat{\alpha}_{\rm h_N 5}, \hat{\alpha}_{\rm h_N}, \hat{\alpha}_{\rm h_N 95})$. The confidence interval obtained in this way appears to always be greater than the one obtained on the estimate performed on simulated trees, as shown with the green and red lines in S3 Fig.

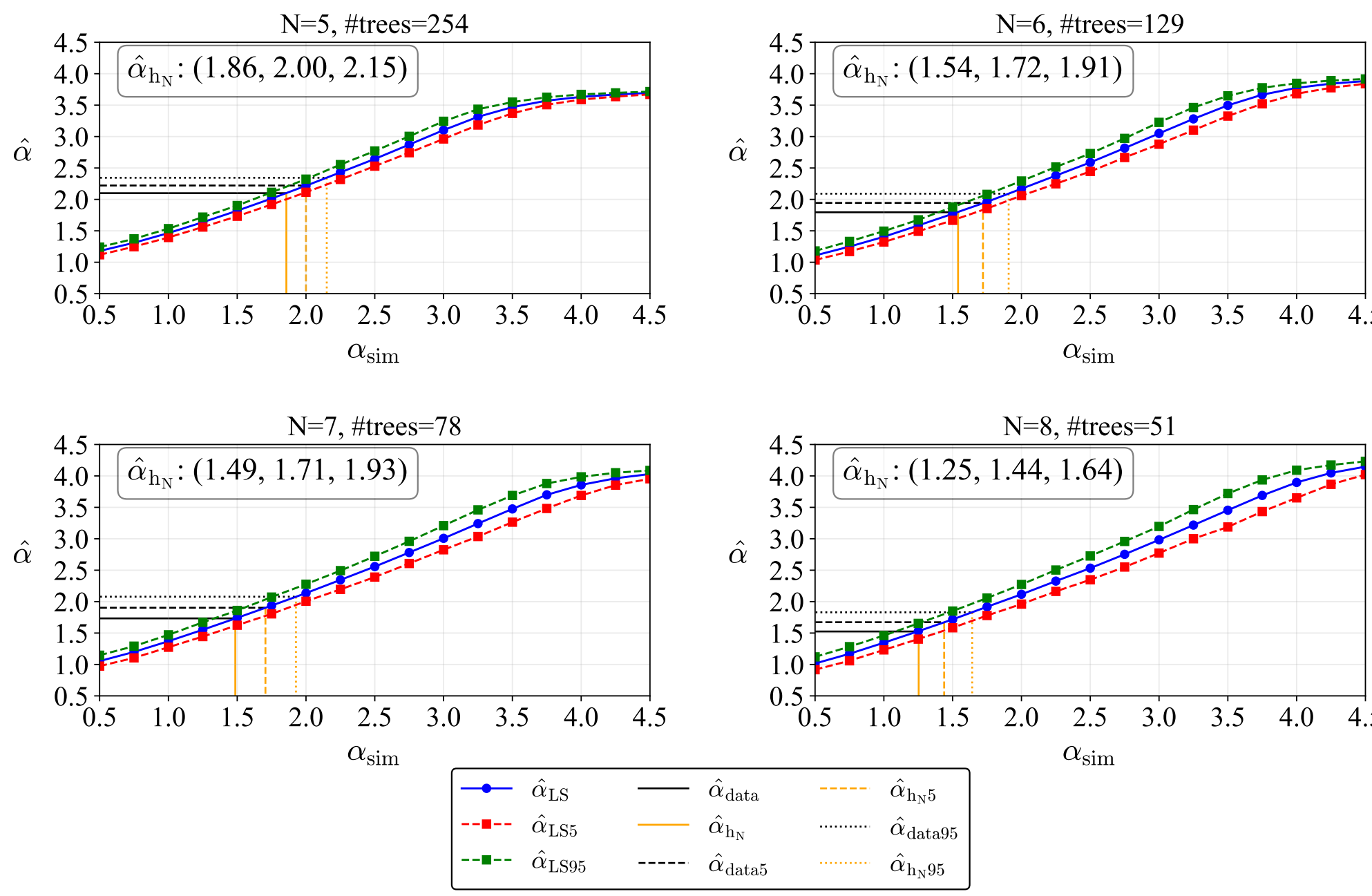

S3 Fig. Application of the method presented in S4.1 Appendix to trees of wave 4 with size $N = 5, 6, 7, 8$. The blue, red and green points are respectively the average, the 5th and the 95th percentiles of the 1000 least squares estimates of α obtained fitting the simulated hopcount distribution (simulation parameters are $\alpha_{\rm sim}$, number of trees, and tree size), with the expression (S1). The black lines represent the average, the 5th, and the 95th percentiles of the least squares estimates of α obtained from the 1000 resampled empirical hopcount distributions. The orange lines are the projection on the x axis of the intersection between the blue and the black lines. The orange values on the x-axis represent the final estimate of the shape parameter of the infection time distribution, as reported in the top left box.

S4.2 Estimate of non-Markovian dynamics from root outdegree distribution

We propose an alternative method to estimate α from the data employing only the root outdegree distribution at a given tree size N.

Part 1: simulations

1. We select a wave in the dataset and we extract the number of trees n_{trees} for any given tree size N. We exclude the trees with size $N < 5$ and for which $n_{\text{trees}} < 3$.

2. For each tree size N we perform 10^5 Monte Carlo simulations of a non-Markovian SI process on the complete graph with N nodes varying the Weibull parameter α_{sim} in $[0.5, 4.5]$, with steps of 0.25. We extract the root outdegree distribution $\hat{\Pr}[d_{\text{root}} = k; \alpha_{\text{sim}}]$ for any α_{sim}.

Part 2: data

1. For any set of n_{trees} trees of size N in the dataset related to the selected wave, we build $N_{\text{bs}} = 1000$ datasets resampling with replacement the initial set of trees.

2. For each resampled dataset we obtain a n_{trees}-dimensional vector of root outdegrees $d_{\text{bs}}^{(j)}$, with $j = 1, ..., N_{\text{bs}}$. We compute the log-likelihood of observing the root outdegree vector according to the distribution $\hat{\Pr}[d_{\text{root}} = k; \alpha_{\text{sim}}]$:

$$\ell(\alpha_{\text{sim}}; d_{\text{bs}}^{(j)}) = \sum_{i=1}^{n_{\text{trees}}} \log(\hat{\Pr}[d_{\text{root}} = (d_{\text{bs}}^{(j)})_i; \alpha_{\text{sim}}]), \tag{S9}$$

   and we take the value of α_{sim} for which $\ell(\alpha_{\text{sim}}; d_{\text{bs}}^{(j)})$ is maximum:

$$\hat{\alpha}_{\text{d}_\text{r}}^{(j)} = \underset{\alpha_{\text{sim}} \in A}{\arg\max}[\ell(\alpha_{\text{sim}}; d_{\text{bs}}^{(j)})],$$

   where A is the set of α_{sim} used in the simulation part.

3. From the distribution of the $\hat{\alpha}_{\text{d}_\text{r}}^{(j)}$ extracted from each bootstrapped sample, we compute the mean, the 5th and the 95th percentiles. For each tree size N in the dataset we obtain three values characterizing the distribution of the maximum likelihood estimate $(\hat{\alpha}_{\text{d}_\text{r}5}, \hat{\alpha}_{\text{d}_\text{r}}, \hat{\alpha}_{\text{d}_\text{r}95})$.

If after the procedure, the 5th percentile of the distribution of $\hat{\alpha}_{\text{d}_\text{r}5}$ for a given tree size N is bigger than 1, with 95% confidence the α of our model is greater than 1 and the data show non-Markovian properties.

S4.2.1 Root outdegree distribution from simulations

For any tree size N in the dataset, we generate 10^5 simulated infection trees with α_{sim} in $[0.5, 4.5]$ and steps of 0.25. S4 Fig shows the resulting root outdegree distribution for $N = 5, 6, 7, 8$ and for four of the α_{sim} values employed. The black dotted line corresponds to (S2) which, as expected, perfectly overlaps with the Markovian simulations ($\alpha = 1$). The dependence on α_{sim} of the distributions in S4 Fig is also in line with Theorem 1, as the probability to have a higher outdegree for the root increases with α.

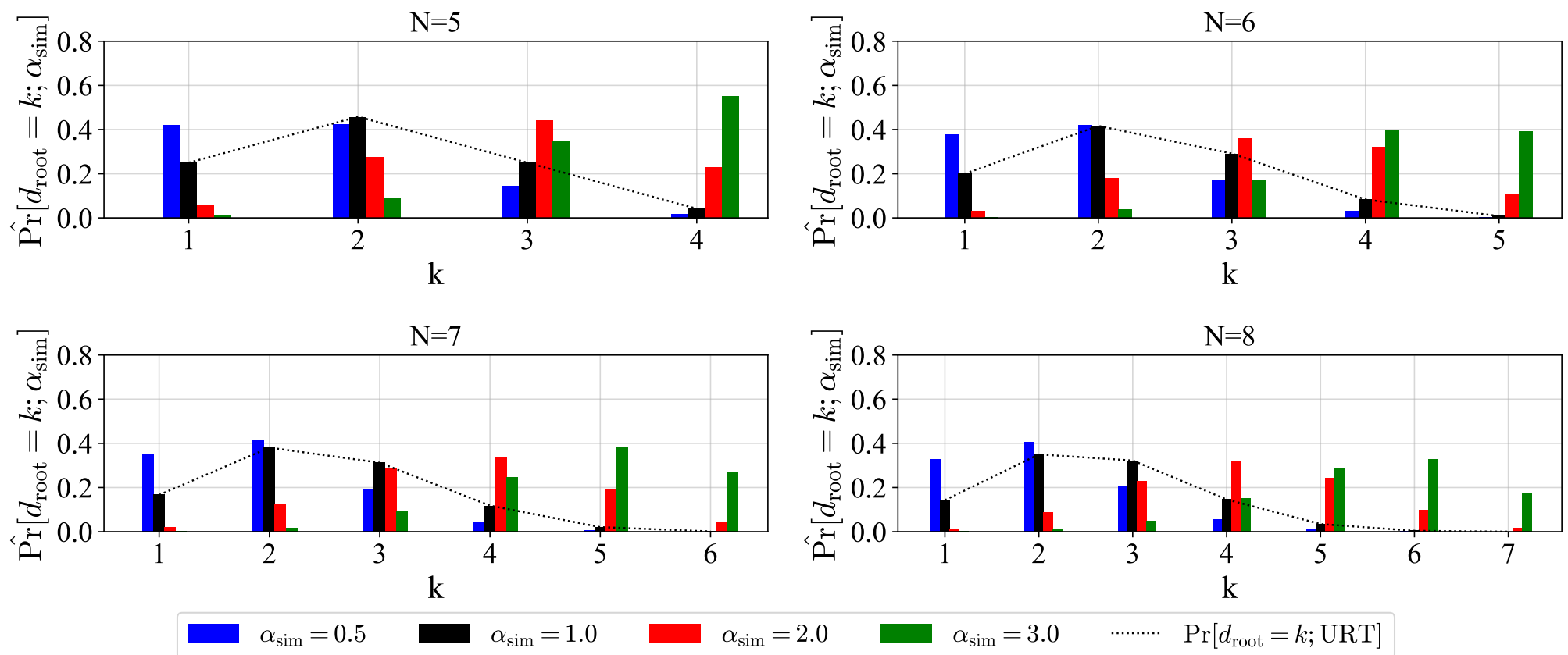

S4 Fig. Root outdegree distribution obtained from 10^5 non-Markovian SI simulation on the complete graph with size $N = 5, 6, 7, 8$, Weibull infection times with rate parameter 1 and shape parameter α_{sim}. In black the Markovian expression ($\alpha = 1$) reported in (S2).

S4.3 Comparative analysis of the two methods to estimate α from the infection trees

We have proposed two methods to estimate the shape parameter α from infection trees: S4.1 Appendix describes the first method based on the hopcount distribution, while S4.2 Appendix describes the second method based on the root outdegree. Here, we assess the robustness and statistical power of the two proposed methods by performing a comparative analysis on synthetic data. We generate synthetic sets of infection trees (see S3 Appendix) using the non-Markovian SI process with Weibull infection times with shape parameter $\alpha_{\text{sim}} \in [1.0, 3.0]$. We vary the tree size $N \in \{5, 10, 15\}$ and the number of trees n_{trees} in a set from 3 to 100.

For each configuration of parameters $\{\alpha_{\text{sim}}, N, n_{\text{trees}}\}$, we generate N_{boot} sets of

independent infection trees. We estimate the shape parameter $\hat{\alpha}_{\mathrm{h}_N}$ using the hopcount distribution method on each set of infection trees, obtaining a distribution of $\hat{\alpha}_{\mathrm{h}_N}$ for the given parameters $\{\alpha_{\mathrm{sim}}, N, n_{\mathrm{trees}}\}$. We then compute the mean, 5th percentile and 95th percentile (i.e. the 90% confidence interval) of the estimated shape parameter $\hat{\alpha}_{\mathrm{h}_N}$. We repeat the same process for the shape parameter $\hat{\alpha}_{d_{\mathrm{r}}}$ estimated using the root outdegree method.

S5 Fig shows the estimated shape parameter $\hat{\alpha}_{\mathrm{h}_N}$ using the hopcount distribution method (green) and the estimated shape parameter $\hat{\alpha}_{d_{\mathrm{r}}}$ using the root outdegree method (red). The infection time shape parameter α_{sim} is on the x-axis and the estimated shape parameter is on the y-axis. The points show the mean and the error bars show the 90% confidence interval which allow us to evaluate the *statistical power* of the estimation method: if the error bars are above the dotted line, indicating the Markovian hypothesis $\alpha = 1$, then the estimation method correctly rejects the Markovian hypothesis with 95% statistical significance.

Each panel shows a different tree size N and tree set size n_{trees}. When we have larger trees N or more trees n_{trees}, the variance in the estimated shape parameter $\hat{\alpha}$ decreases. From left to right, the panels show increasing tree size N and the error bars become smaller for both the hopcount distribution estimation method and the outdegree estimation method. From top to bottom, the panels show increasing tree set size n_{trees}, for which the error bars also become smaller in both estimation methods.

Rather remarkably, we observe that the confidence intervals for the root outdegree estimation method are comparable to those of the hopcount distribution estimation method, even when data is sparse. The root outdegree, despite observing only the initial branching of the infection tree, is extremely sensitive to structural changes and effectively captures the non-Markovian dynamics. For $\alpha_{\mathrm{sim}} = 1.5$, we observe that both estimation methods correctly reject the Markovian hypothesis with a p-value of 0.05 when the number of trees $n_{\mathrm{trees}} = 10$ on all of the tree sizes N. If we have a very small number of trees $n_{\mathrm{trees}} = 3$, the Markovian hypothesis cannot be rejected with 95% statistical significance for $\alpha_{\mathrm{sim}} = 1.5$ unless the trees are sufficiently large $N = 15$. However, if the infection time shape parameter is sufficiently large $\alpha_{\mathrm{sim}} > 2$, both estimation methods are able to correctly reject the Markovian hypothesis (95% statistical significance) with just $n_{\mathrm{trees}} = 3$ trees of size $N = 5$.

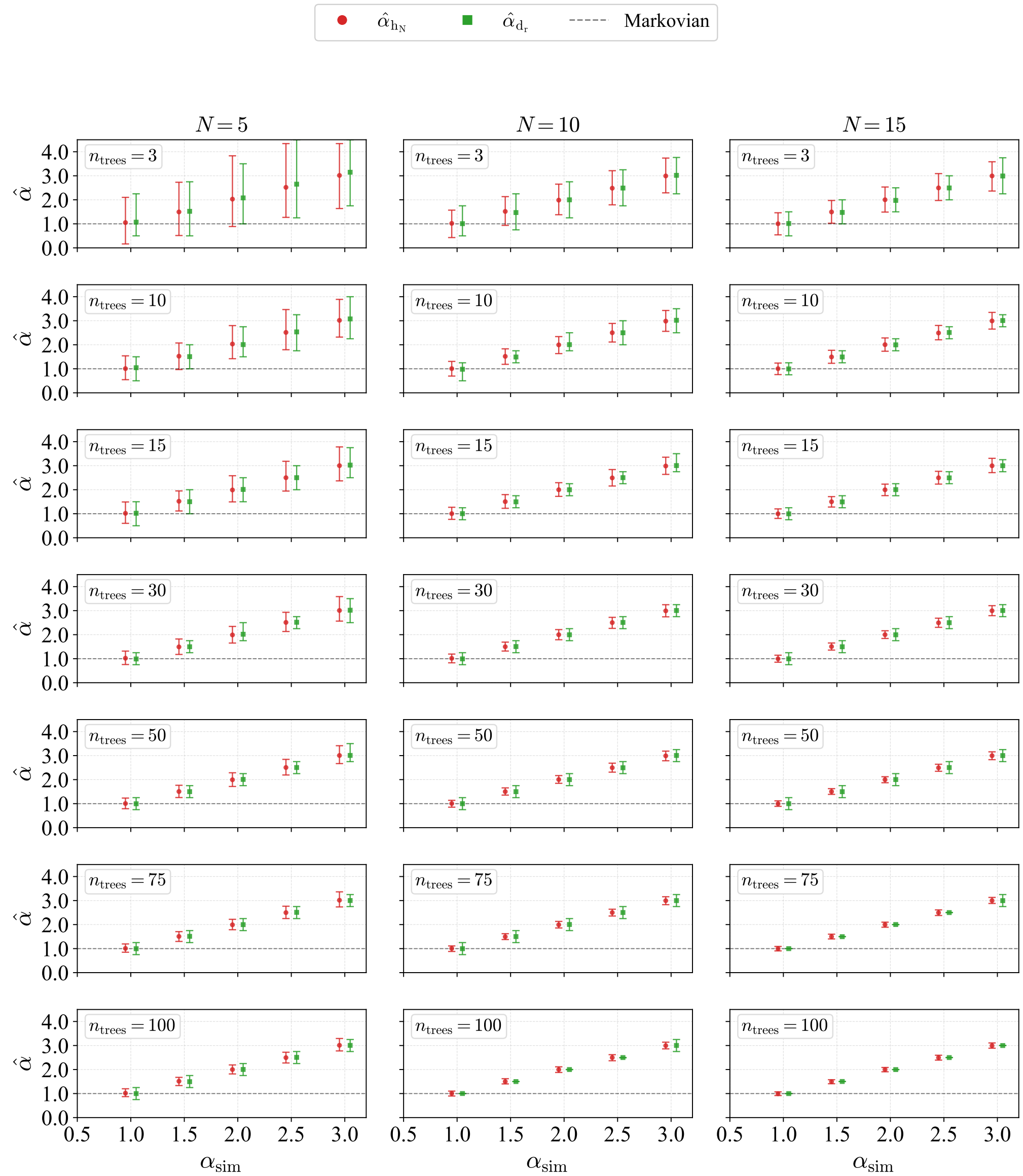

S5 Fig. Comparative analysis of the hopcount and root outdegree estimation methods (red and green markers respectively). The figure displays the estimated shape parameter $\hat{\alpha}$ (mean, along with 5th and 95th percentiles) as a function of the number of trees (n_{trees}), tree size (N), and varying true values of $\alpha_{\text{sim}} \in [1.0, 3.0]$. The baseline for the Markovian hypothesis ($\alpha = 1$) is the dashed gray line. Both estimators accurately track the true parameter α, with uncertainty bounds progressively narrowing as the sample size (n_{trees}) and tree size (N) increase.

S5 Additions to the Results

S5.1 Markovian benchmark vs Non-Markovian process

S5.1.1 Markovian SI vs non-Markovian SI

We compare the hopcount distribution of the infection trees obtained from the Cyprus contact tracing data with the hopcount distribution of a Markovian SI process and a non-Markovian SI process. We quantify the difference between the distributions using the Wasserstein distance. For two discrete probability distribution functions (i.e. cumulative distributions) $F_1(x)$ and $F_2(x)$ with support X, the Wasserstein-1 distance is defined as

$$W_1(F_1(x), F_2(x)) = \sum_{k \in X} |F_1(k) - F_2(k)| \tag{S10}$$

In our case, $F_1(x) = \Pr[X_1 \leq x]$ is the distribution function of the hopcount obtained from simulations of a Markovian or non-Markovian SI process, $F_2(x) = \Pr[X_1 \leq x]$ is the distribution function of the hopcount obtained from the contact tracing data, and the support $X = \{k \,|\, k \in \mathbb{N},\, 0 \leq k < N\}$, where N is the size of the infection tree. The distribution functions are computed summing (S8) over the support.

For each infection tree size N in each wave of the Cyprus contact tracing data, we compute the Weibull shape parameter $\hat{\alpha}_{h_N}$ which best fits the data according to the Method presented in S4.1 Appendix (we omit wave 2 due to insufficient data). We then perform 10^4 Monte Carlo trials of a SI simulation (S3 Appendix) with Weibull infection time with shape parameter $\alpha_{\text{sim}} = \hat{\alpha}_{h_N}$ and compute the Wasserstein distance (S10) between the distribution function $F_1(x)$ of the hopcount of a non-Markovian SI process and the distribution function $F_2(x)$ of the hopcount of the contact tracing data. Likewise, we perform 10^4 Monte Carlo trials of a SI simulation with exponential infection time ($\alpha_{\text{sim}} = 1$) and compute the Wasserstein distance (S10) between the distribution function $F_1(x)$ of the hopcount of a Markovian SI process and the distribution function $F_2(x)$ of the hopcount of the contact tracing data.

In order to fairly compare between different tree sizes N, we normalize the Wasserstein distance by dividing by $N - 1$. S6 Fig shows the normalized Wasserstein distance between the hopcount distribution of the Markovian SI process and the contact tracing data in grey, and the Wasserstein distance between the hopcount distribution of

the non-Markovian SI process and the contact tracing data in orange. For every tree size N in every wave of the contact tracing data, the Wasserstein distance for the non-Markovian SI process is much lower than that of the Markovian SI process.

A Markovian SI process inherently assumes a constant infection rate due to the memoryless property of the exponential distribution. By definition, a non-Markovian SI process therefore has a time-varying infection rate. The Markovian assumption of a constant infection rate is unlikely to be realistic in real epidemics due to the temporal complexities of real-world infection processes. By using a Weibull distribution to model the non-exponential infection time and tuning the shape parameter α, we are able to much better reproduce the hopcount distribution of the contact tracing data.

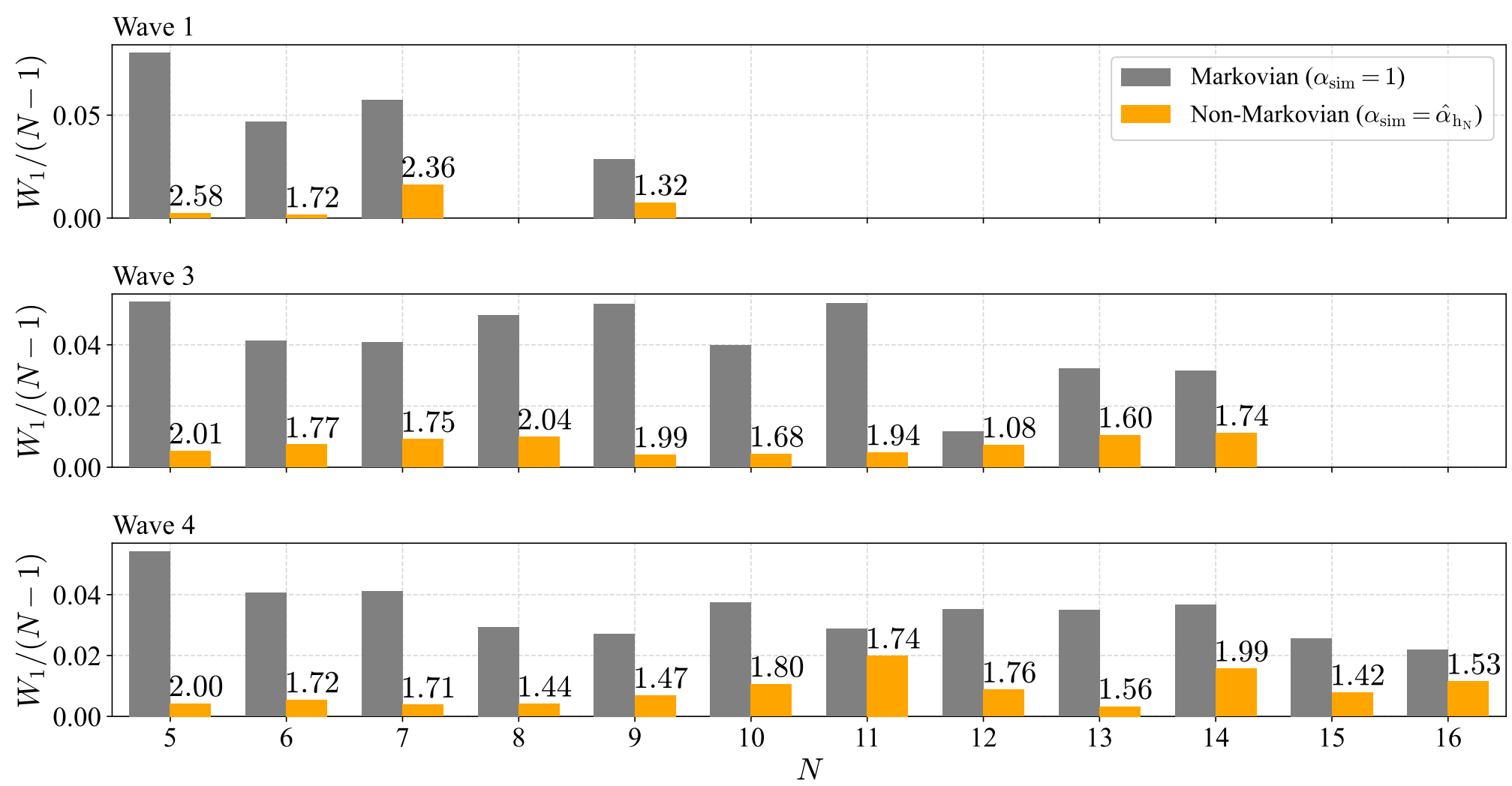

S6 Fig. Normalized Wasserstein distance between the hopcount distribution of the infection trees obtained from the Cyprus contact tracing data and the hopcount distribution obtained from 10^4 Monte Carlo simulation of an SI process on the complete graph of size N. In gray the distance for the simulations with exponential infection times $\alpha = 1$. In orange the distance for the simulations with Weibull infection times with shape parameter $\hat{\alpha}_{h_N}$ indicated above each orange bar. The $\hat{\alpha}_{h_N}$ values are obtained from the best fit to the data according to the Method presented in Sec S4.1. Results presented for Wave 1 (top), Wave 3 (middle), and Wave 4 (bottom) across available tree sizes N.

S5.1.2 Markovian SEI vs non-Markovian SI

In the previous section, we have shown that the Markovian SI process cannot reproduce the hopcount distribution obtained from infection trees in the Cyprus contact tracing data. In a Markovian SI process, the infection rate is constant meaning that as soon as a node i becomes infected, node i starts infecting all of its neighbors at a constant rate.

However, in many real infectious diseases, such as COVID-19 [9, 10], there is an incubation period: after an individual becomes infected, there is a period of time before the individual becomes contagious [11]. During the incubation period, the individual cannot yet transmit the virus to others. The incubation period may be modeled explicitly using the SEI model which extends the SI model with an additional Exposed compartment. When node j infects node i, node i becomes exposed but cannot yet infect others. After the incubation time T_i^{inc}, node i becomes infectious and can now infect its neighbors.

Here, we consider whether a Markovian SEI process can reproduce the hopcount distribution of the infection trees obtained from the Cyprus contact tracing data. In the Markovian SEI process, the incubation time T^{inc} is exponentially distributed with incubation rate γ and the infection time T is exponentially distributed with rate β. When node i becomes infected, the time before node i can infect a susceptible neighbor j is therefore $H_{i\to j} = T_i^{\text{inc}} + T_{i\to j}$ with probability density function [3, Sec. 3.3.1]

$$f(t) = \frac{\gamma\beta}{\gamma - \beta}(e^{-\beta t} - e^{-\gamma t}) \tag{S11}$$

if $\gamma \neq \beta$. If $\gamma = \beta$, then the density function is $f(t) = \beta^2 t e^{-\beta t}$. The single maximum of $f(t)$ occurs at $t_{\max} = \frac{\log(\beta)-\log(\gamma)}{\beta-\gamma}$ and $t_{\max} = \frac{1}{\beta}$ if $\gamma = \beta$. The density $f(t)$ in (S11) of two independent exponential random variables is zero in $t = 0$ and unimodal, similarly to a Weibull density function with $\alpha > 1$.

Since the incubation rate γ and infection rate β are not known, we define the normalized incubation time $E[T^{\text{inc}}]/E[T] = \beta/\gamma$ as a parameter and consider a range of possible values $\beta/\gamma \in \{0.1, 0.3, 0.5, 1, 5\}$. A normalized incubation time $\beta/\gamma = 0.5$ implies that the mean incubation time $E[T^{\text{inc}}]$ is half the mean infection time $E[T]$. For each normalized incubation time β/γ, we perform 10^4 Monte Carlo trials of a Markovian SEI simulation (S3 Appendix). As in the previous section, we use the Wasserstein distance (S10) to quantify the distance between the distribution function obtained from simulations of the hopcount in a Markovian SEI process $F_1(x)$ and the distribution function of the hopcount obtained from the contact tracing data $F_2(x)$.

In S7 Fig, we plot the normalized Wasserstein distance between the hopcount distribution of the Markovian SEI process and the contact tracing data in grey, using

their distribution functions. Darker shades of grey correspond to a higher normalized incubation time β/γ. For comparison, we also plot the normalized Wasserstein distance between the hopcount distribution of the non-Markovian SI process and the contact tracing data in orange (the results of the previous section in S6 Fig).

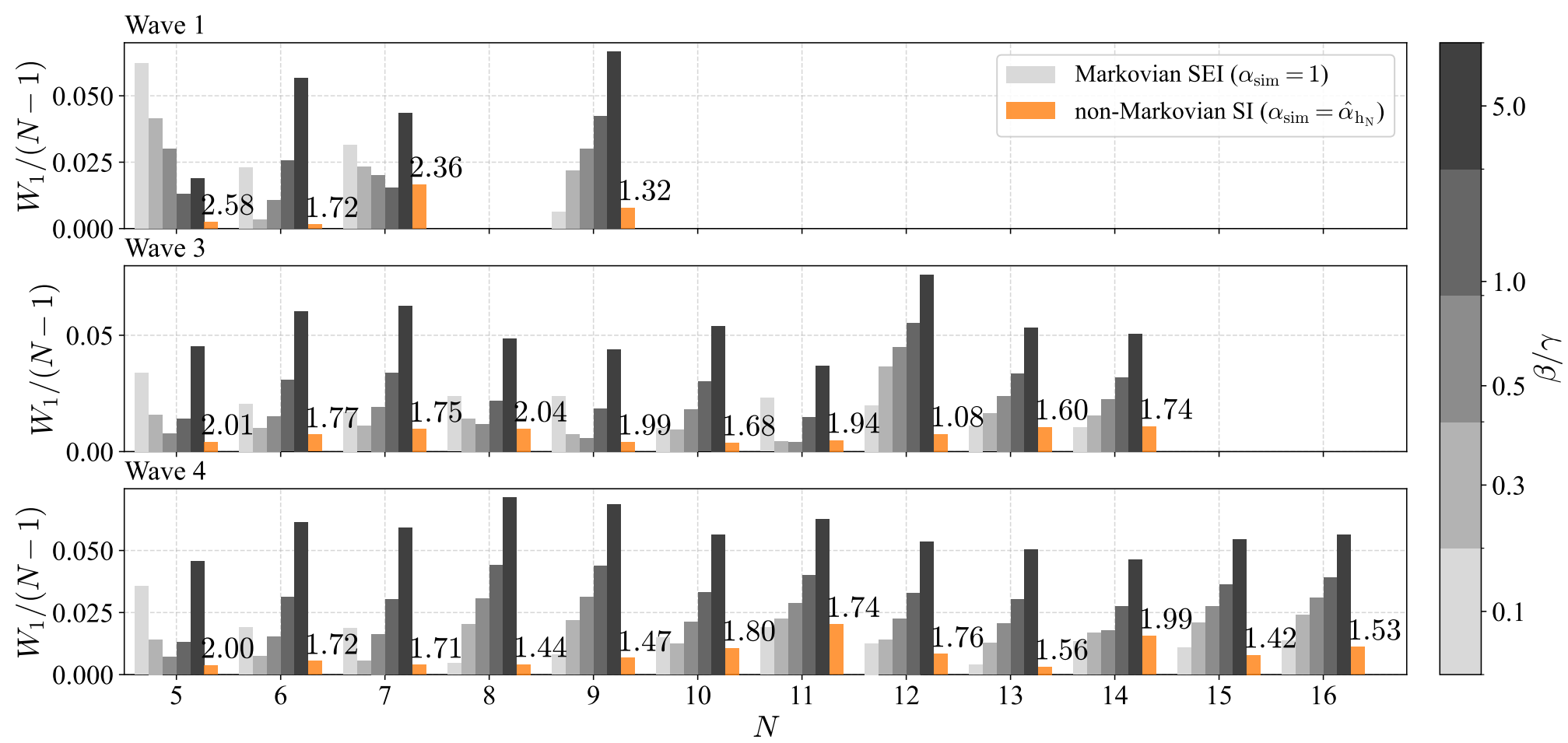

S7 Fig. Normalized Wasserstein distance between the hopcount distribution obtained from Cyprus contact tracing data and the one obtained from 10^4 Monte Carlo simulation of an SEI process on the complete graph of size N. In gray scale the distance for the simulations with exponential infection and incubation times $\alpha = 1$, with rate parameters β and γ, respectively. In orange the distance for the simulations with Weibull infection times with shape parameter $\hat{\alpha}_{h_N}$ indicated above each orange bar. The $\hat{\alpha}_{h_N}$ values are obtained from the best fit to the data according to the Method presented in S4.1 Appendix.

S1 Table summarizes the waves and parameters for which the Markovian SEI process has a lower Wasserstein distance than the non-Markovian SI process with Weibull infection time. Although the Markovian SEI process sometimes achieves a better fit, the assumption that the incubation time and infection time are both exponentially distributed is unrealistic and may lead to misleading conclusions [12, 13]. In the Markovian SEI process, the fitting parameter is the normalized incubation time $\beta/\gamma = E[T^{\text{inc}}]/E[T]$, which ranges from 0.1 to 1.0 in S1 Table. These values are unrealistic and contradict established COVID-19 clinical timelines [10, 12]: the incubation rate γ is generally smaller than the transmission rate β, corresponding to a normalized incubation time β/γ bigger than 1 [10, 12].

Wave	N	β/γ	SEI	SI
1	7	1.0	0.0154	0.0167
1	9	0.1	0.0064	0.0079
3	11	0.3	0.0046	0.0049
3	11	0.5	0.0041	0.0049
3	14	0.1	0.0104	0.0110
4	11	0.1	0.0191	0.0204
4	14	0.1	0.0133	0.0156

S1 Table. Infection trees sets in the Cyprus contact tracing data where the Markovian SEI Wasserstein distance from the hopcount distribution computed from data is smaller than non-Markovian SI one obtained from simulations.

Hence, we conclude that the Markovian SEI process is not realistic for modeling the hopcount distribution of infection trees obtained from the contact tracing data. Although the Markovian SEI process may be able to reproduce the hopcount distribution, the inferred physics of the spreading process are unrealistic and lead to misleading conclusions about the duration of the incubation and infection time. In presence of better data about the incubation and infection times we could build a more suitable non-Markovian SEI process which would provide a better fit to the data. In contrast, our non-Markovian SI process with Weibull infection time only models the net effect of the spreading process and it is parameterized by a single shape parameter α which is consistently above 1.0 across different waves and tree sizes (Fig 8).

S5.2 Shape parameter α in non-Markovian SIR

In this section, we test whether the infection time shape parameter obtained from the Cyprus contact tracing data, is still larger than 1 when a more general susceptible-infected-recovered (SIR) model is considered. The only difference with the previous SI model is that a node i can recover after a random time T_i^{rec} hence stopping its infection attempts.

We assume that both the infection and the recovery times are Weibull distributed with shape parameter α and rate parameter β and δ respectively. We define the ratio between the two rates as $\tau = \beta/\delta$. We then simulate (see S3 Appendix) a non-Markovian SIR on a complete graph K_N for any available tree size in the dataset, for different values of $\alpha \in [0.5, 3.5]$ and $\tau \in [0.5, 2]$. We then extract the α values for which the Wasserstein distance (S10) between the empirical and the simulated hopcount distribution is minimum. Given that in an SIR process not every node in the graph is

infected, we only retain simulations where all nodes get infected. This choice allows us to avoid any additional assumption about a larger underlying graph and is the closest possible to the original SI model.

S8 Fig shows that the shape parameter $\hat{\alpha}$ is almost always larger than the previous estimate $\hat{\alpha}_{h_N}$ obtained from data with the Method presented in S4.1 Appendix. The results indicate that when recoveries are introduced, the process may need a larger shape parameter α in order to reproduce the data. The infection process needs indeed to compensate for the competition with the recovery process and a larger α increases the number of successful transmissions from the early infected nodes. The results confirm that our main findings summarized in Fig 8, i.e. $\hat{\alpha} > 1$ for all tree sizes and waves, are robust to the introduction of the recovery compartment.

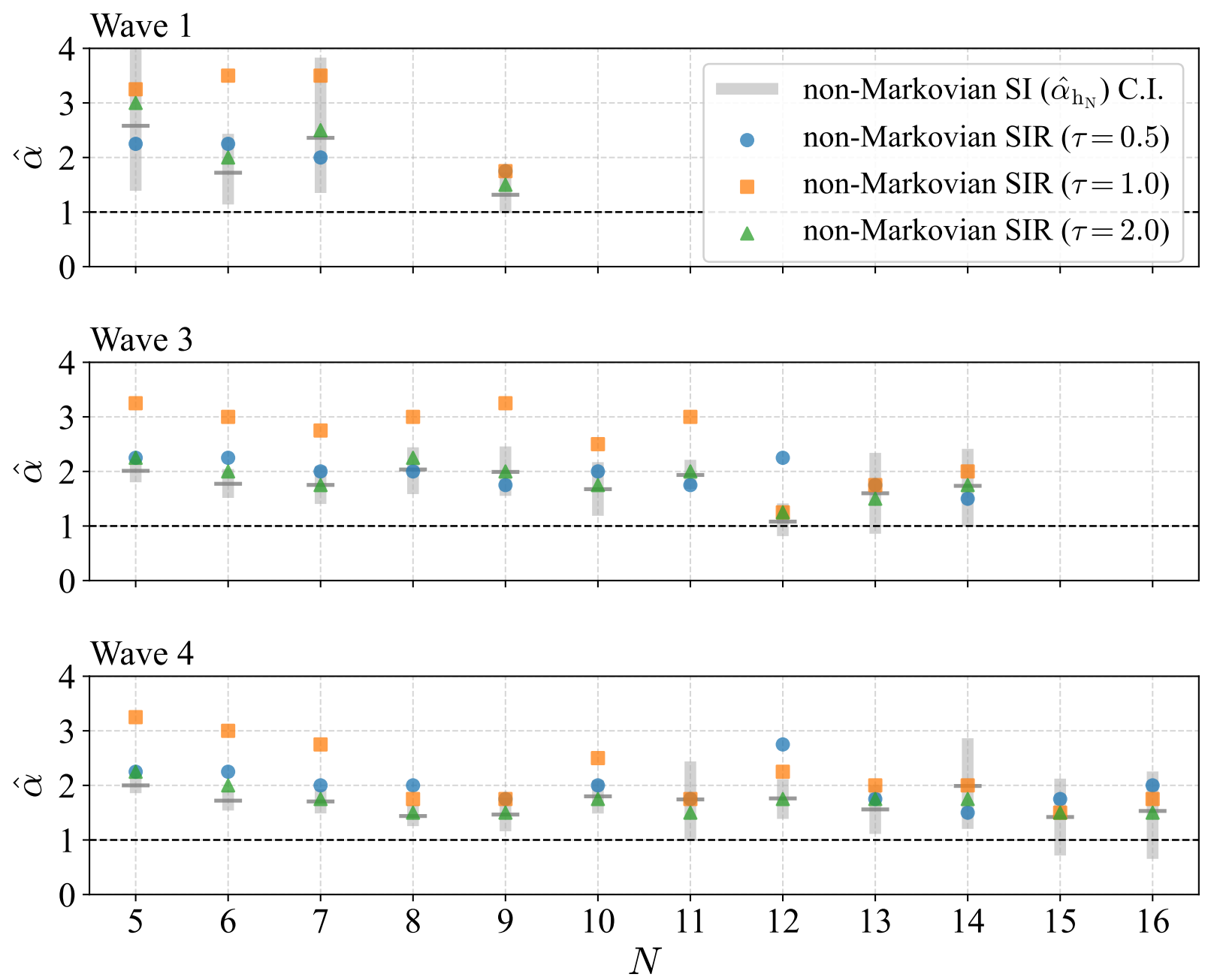

S8 Fig. Estimated Weibull shape parameter $\hat{\alpha}$ obtained by minimizing the Wasserstein distance between the hopcount distribution obtained from the Cyprus contact tracing data and the simulated hopcount distributions computed from 10^3 Monte Carlo simulations of the non-Markovian SIR process with Weibull infection and recoveries (same shape parameter α) on a complete graph K_N. Results are shown for different values of the rate ratio between the infection and recovery Weibull rate parameters $\tau = \beta/\delta$: $\tau = 0.5$ (blue circles), $\tau = 1.0$ (orange squares), and $\tau = 2.0$ (green triangles). The gray shaded regions and horizontal gray bars indicate the confidence intervals and mean estimates for the non-Markovian SI model simulated with shaper parameter $\hat{\alpha}_{h_N}$ obtained with the Method presented in S4.1 Appendix. The horizontal dashed line at $\alpha = 1.0$ represents the baseline for exponential infection and recovery times. Results presented for Wave 1 (top), Wave 3 (middle), and Wave 4 (bottom) across available tree sizes N.

S5.3 Outdegree distribution in wave 3 and 4

For waves 3 and 4, the authors of [10] do not report estimates of the effective reproduction number but provide publicly available code to estimate it from daily cases using the meta-population model by Peng et al. [14]. We apply the code to estimate the daily effective reproduction number to their data corresponding to the period of our waves 3 and 4. Results are shown in S9 and S10 Figs. For waves 3 and 4 it is even more evident that our effective daily reproduction number $\hat{R}(t; 1 \text{ day})$, estimated from the outdegree distribution of the infection trees, consistently falls below the effective reproduction number $\hat{R}_1$ computed with the compartmental model.

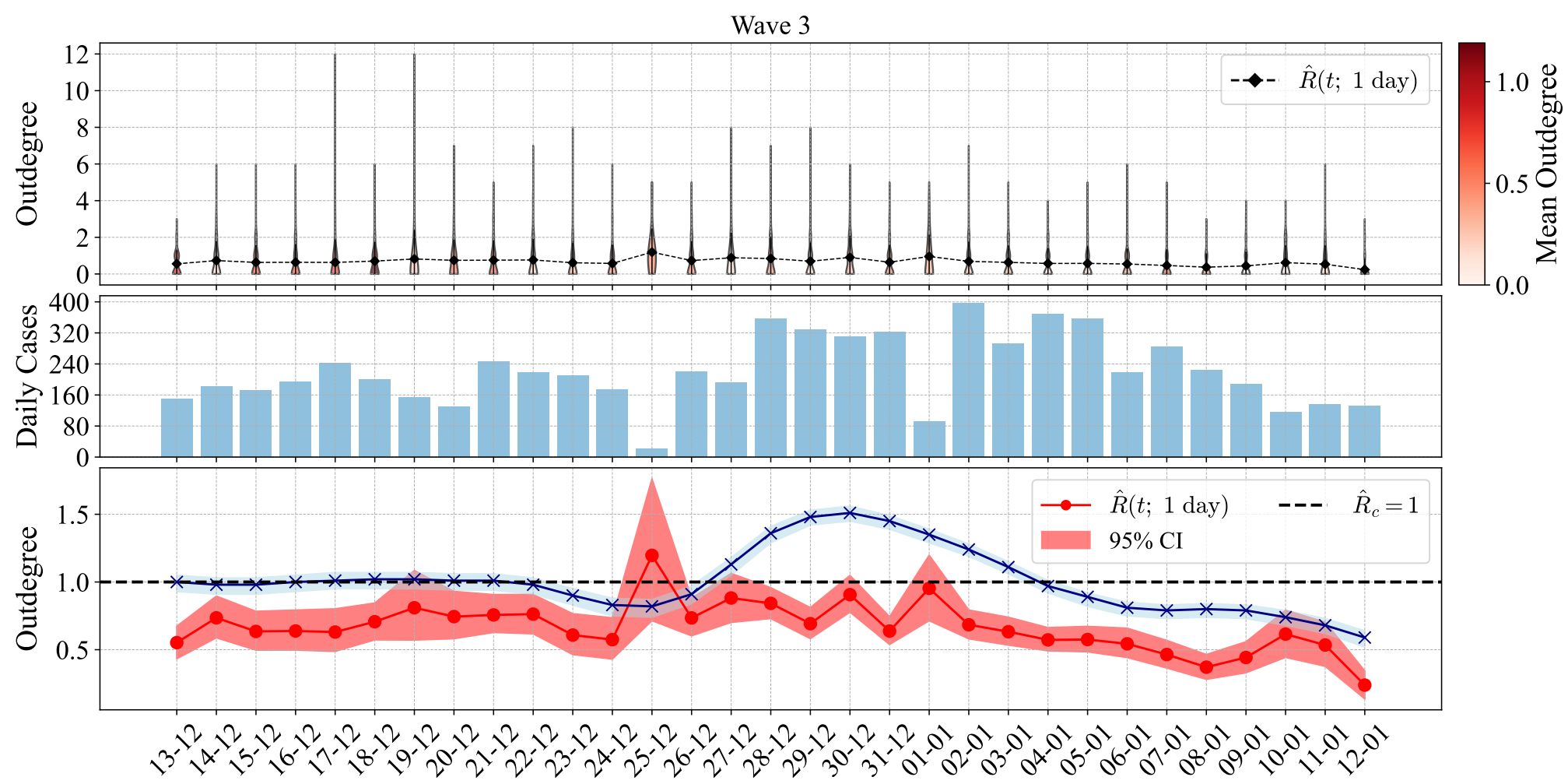

S9 Fig. Daily out-degree distribution (top) and reported case counts (bottom) during the third COVID-19 wave in Cyprus. Violin plots represent the distribution of secondary infections per case per day; color intensity indicates the mean outdegree. The lower subplot shows the corresponding number of daily reported cases. Evolution of the effective reproduction number $\hat{R}(t; 1 \text{ day})$ in the third wave of the COVID-19 epidemic in Cyprus. The red line shows the mean outdegree for each day derived from bootstrapped samples, with the 95% confidence intervals indicated as a red area. The blue line represents the effective reproduction number estimated using the method and the data from [10] for the same epidemic event. In dashed black the reference line at the critical value $\hat{R}_c = 1$.

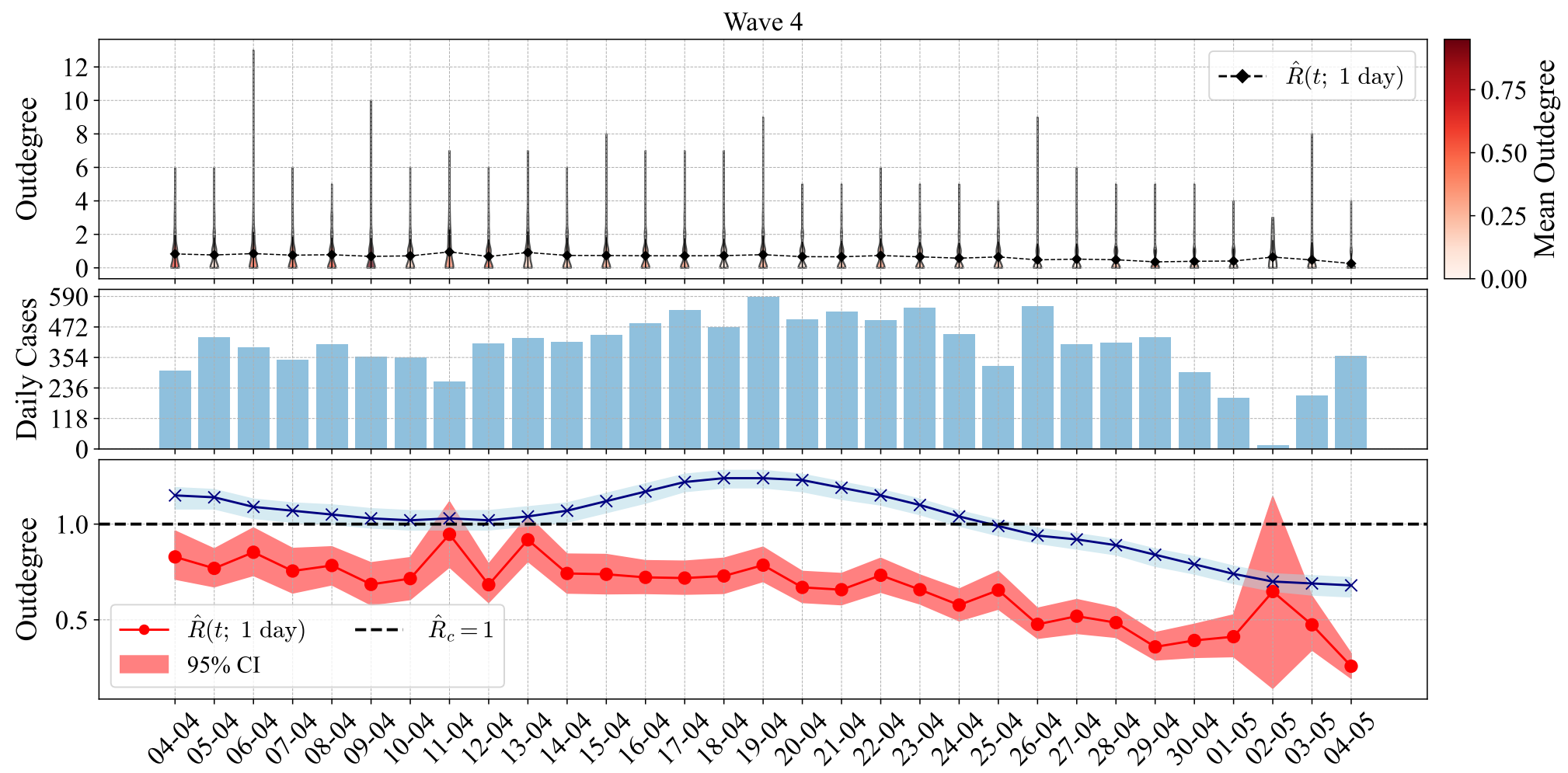

S10 Fig. Daily out-degree distribution (top) and reported case counts (bottom) during the fourth COVID-19 wave in Cyprus. Violin plots represent the distribution of secondary infections per case per day; color intensity indicates the mean outdegree. The lower subplot shows the corresponding number of daily reported cases. Evolution of the effective reproduction number $\hat{R}(t; 1 \text{ day})$ in the fourth wave of the COVID-19 epidemic in Cyprus. The red line shows the mean outdegree for each day derived from bootstrapped samples, with the 95% confidence intervals indicated as a red area. The blue line represents the effective reproduction number estimated using the method and the data from [10] for the same epidemic event. In dashed black the reference line at the critical value $\hat{R}_c = 1$.

S11 Fig shows the results across all four waves when applying a pruning technique to mitigate the systematic underestimation of the effective reproduction number.

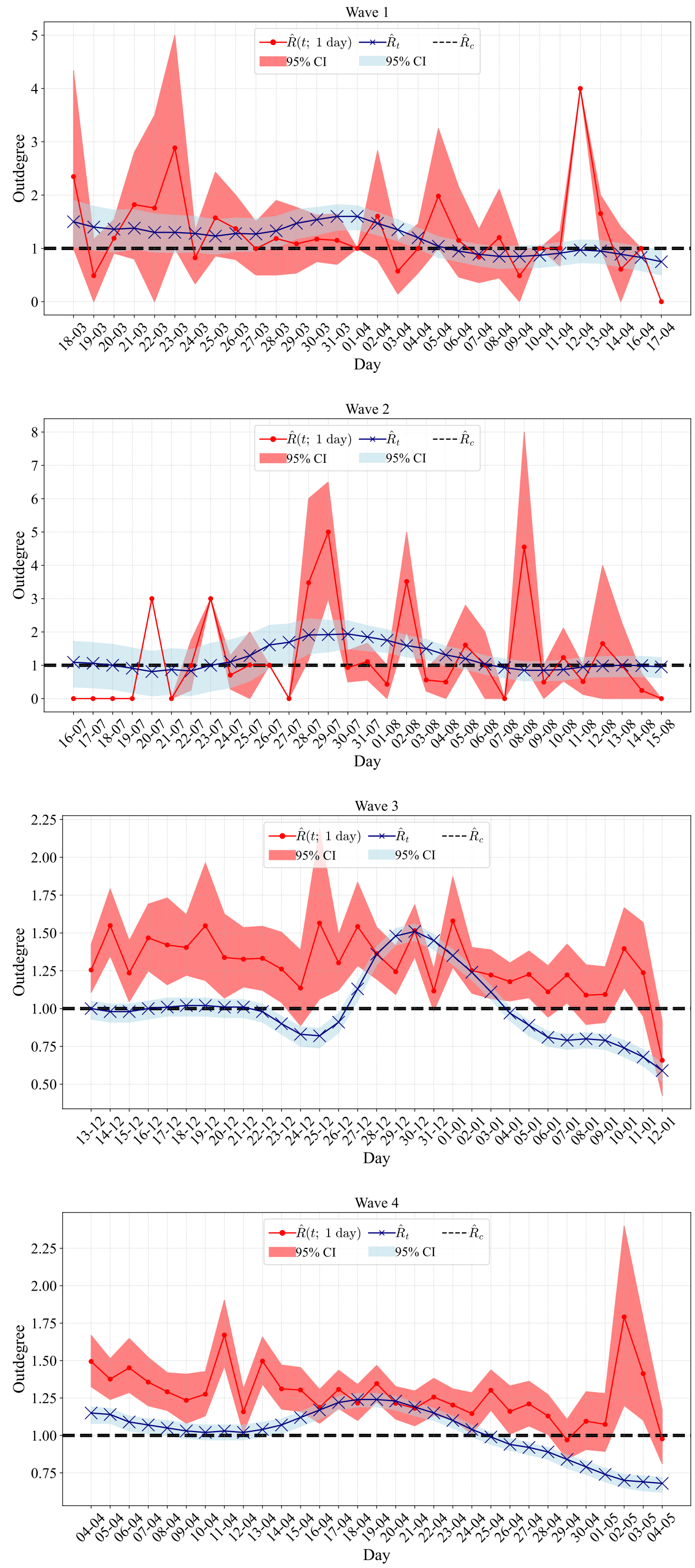

S11 Fig. Evolution of the effective reproduction number $\hat{R}(t; 1 \text{ day})$ in the 4 waves of the COVID-19 epidemic in Cyprus having removed the last level of all the infection trees. The red line shows the mean outdegree for each day derived from bootstrapped samples, with the 95% confidence intervals indicated as a red area. For wave 1 and 2 the blue line represents the effective reproduction number reported in Fig. 11 of [10] and in [15], respectively. For waves 3 and 4 the blue line represents the effective reproduction number estimated using the method and the data from [10] for the same epidemic event. In dashed black the reference line at the critical value $\hat{R}_c = 1$.

References

1. Hale T, Angrist N, Goldszmidt R, Kira B, Petherick A, Phillips T, et al. A global panel database of pandemic policies (Oxford COVID-19 Government Response Tracker). Nature human behaviour. 2021;5(4):529-38.

2. Press and Information Office of Cyprus. COVID-19 Information Portal. Press and Information Office of Cyprus; 2025. Accessed: March 20, 2025. Available from: `https://www.pio.gov.cy/coronavirus/eng`.

3. Van Mieghem P. Performance analysis of complex networks and systems. Cambridge University Press; 2014.

4. Van Mieghem P, Hooghiemstra G, van der Hofstad R. A scaling law for the hopcount in internet. Delft University of Technology, report. 2000;2000125.

5. Bhamidi S, Van Der Hofstad R. Weak disorder asymptotics in the stochastic mean-field model of distance. Ann Appl Probab 22(1): 29-69. 2012.

6. Van Mieghem P, van Langen S. Influence of the link weight structure on the shortest path. Physical Review E—Statistical, Nonlinear, and Soft Matter Physics. 2005;71(5):056113.

7. Van Mieghem P, Magdalena SM. Phase transition in the link weight structure of networks. Physical Review E—Statistical, Nonlinear, and Soft Matter Physics. 2005;72(5):056138.

8. Dijkstra EW. A note on two problems in connexion with graphs. In: Apt KR, Hoare T, editors. Edsger Wybe Dijkstra: His Life, Work, and Legacy. ACM Books; 2022. p. 287-90. doi:10.1145/3544585.3544600.

9. Zhao S, Tang B, Musa SS, Ma S, Zhang J, Zeng M, et al. Estimating the generation interval and inferring the latent period of COVID-19 from the contact tracing data. Epidemics. 2021;36:100482.

10. Agapiou S, Anastasiou A, Baxevani A, Nicolaides C, Hadjigeorgiou G, Christofides T, et al. Modeling the first wave of Covid-19 pandemic in the Republic of Cyprus. Scientific Reports. 2021;11(1):7342.

11. Nelson KE, Williams CM. Infectious disease epidemiology: theory and practice. Jones & Bartlett Publishers; 2014.

12. Zhang L, Zhu J, Wang X, Yang J, Liu XF, Xu XK. Characterizing COVID-19 transmission: incubation period, reproduction rate, and multiple-generation spreading. Frontiers in Physics. 2021;8:589963.

13. Backer JA, Eggink D, Andeweg SP, Veldhuijzen IK, van Maarseveen N, Vermaas K, et al. Shorter serial intervals in SARS-CoV-2 cases with Omicron BA.1 variant compared with Delta variant, the Netherlands, 13 to 26 December 2021. Eurosurveillance. 2022;27(6):pii=220042.

14. Peng L, Yang W, Zhang D, Zhuge C, Hong L. Epidemic analysis of COVID-19 in China by dynamical modeling. arXiv preprint arXiv:200206563. 2020.

15. Office CGP. National Report on COVID-19 in Cyprus up to 22/09/2020; 2020. Accessed: 2025-04-07. Available from: `https://www.pio.gov.cy/coronavirus/pdf/ep22092020.pdf`.
