# Supplementary material for: Non-Markovian dynamics and effective reproduction number in COVID-19: Evidence from Cyprus contact tracing data
Source: PLoS Comput Biol. 2026 Aug 21;22(8):e1014578. doi: 10.1371/journal.pcbi.1014578 (PMC13496441; doi:10.1371/journal.pcbi.1014578)
Supplement: Supplementary file 2 [file pcbi.1014578.s019.pdf]

13th May, 2026

Dear Editor, Dear Reviewers,

We sincerely thank you for your insightful and constructive feedback on our manuscript. We greatly appreciate the time and effort invested in providing detailed feedback which has helped us improve the clarity and quality of our work. We report here your comments and we reply **(R)** in red to all of them individually. All the changes in the manuscript are highlighted in red.

We have updated the dataset used for our analysis and made it permanently available at the link indicated in the Data Availability statement. In the main text, we have added Figure 2 and updated all existing figures, with the exception of Figures 4 and 5. While we have kept the overall structure of the manuscript unchanged, we have made various changes to the text belonging to the Methods, Results and Discussion sections. These revisions clarify specific properties of the dataset and our methodology, and allow us to incorporate new findings derived from additional simulations, which are detailed in the Supporting Information.

We denote sections and figures in the Supporting Information with the prefix "S". To improve logical flow, we have replaced the previous Section S4 with Section S3; this change ensures that our simulation technique can be more easily extended to include additional compartments in the epidemic model.

Furthermore, we have added Section S4.3 to provide a comparative analysis of the two methods used to estimate the parameter alpha from infection trees, summarizing the results for synthetic data in Figure S3. In Section S5.1 and Figure S4, we quantify the performance of the non-Markovian fit relative to the Markovian benchmark. In Section S5.1.2 we examine the impact of introducing incubation time into our analysis; these results are presented in Figure S5 and Table S1. Finally, in Section S5.2 and Figure S6, we illustrate how the inclusion of recoveries affects the non-Markovian measures within the dataset. We have also updated all other figures in the Supporting Information to ensure formatting consistency.

Reviewer #1: This manuscript presents a well-structured and insightful study that reconstructs infection trees across multiple COVID-19 waves using detailed contact tracing data. By extending the Markovian SI infection tree framework to a non-Markovian setting, the authors provide an elegant and rigorous quantification of non-Markovianity through the Weibull shape parameter. The analyses of hopcount and outdegree distributions convincingly demonstrate that real-world spreading processes deviate from exponential assumptions, with infection times better described by Weibull distributions (shape > 1). The proposed pruning technique is appropriate and effectively reduces bias in estimating the effective reproduction number. Overall, this is a coherent and methodologically sound work that offers valuable insights for incorporating Non-Markovian dynamics into epidemic modeling.

A major concern is that the authors choose to use the data with at least 1 offspring case in their contact tracing data. If they use this dataset for the estimation of reproduction number, this will introduce the selection bias, and the estimate of reproduction would be lower than the real value (under-estimation).

(R) We are grateful to the Reviewer for highlighting the selection bias in our dataset. We agree that excluding isolated nodes leads to a systematic underestimation of the effective reproduction number. Indeed, as illustrated in Figures 9, 10, 11, S9 and S10, our estimates are consistently lower than the official estimates provided by the Cyprus Ministry of Health (MoH). The only exceptions coincide with periods of high variance in the daily outdegree distribution, which can be explained by the influence of "superspreading" events.

Our decision to restrict the analysis to cases with at least one observed offspring is motivated by the limitations of the data. In particular, cases with zero observed offspring (isolated cases) are fundamentally ambiguous. For such cases, there is no information about how the infection occurred: they may correspond to external introductions into the community or to missing links with already observed cases due to incomplete contact tracing. Considering isolated cases would thus necessitate uninformed assumptions about missing infection links. To maintain the integrity of the contact-tracing evidence, we have opted to stick strictly to the confirmed links.

To address the resulting underestimation, we have proposed to remove the final generation (leaves) from all infection trees. Fig. 12 shows that this approach mitigates the underestimation of the effective reproduction number and provides estimates which reflect more faithfully the evolution of the epidemic spreading. Indeed, since our dataset covers the unfolding of four epidemic waves, for each of them the effective reproduction number is expected to decrease progressively from before to after the peak. The unpruned estimates fail to capture this behaviour (Fig. 12 left), showing little variation or even an increase after the peak. By contrast, the pruned estimates consistently show the expected decreasing trend across all four waves (Fig. 12 right).

Finally, in Fig. S11 we show that pruning the infection trees may often provide an upper bound for the reproduction number compared to the Cyprus MoH reference values (especially for waves 3 and 4 where more data is available).

Reviewer #2: Authors use contact tracing from Cyprus to construct detailed infection trees of the recent SARS-CoV viral epidemic spread. This allows them to determine to what degree that a Markov model, which assumes that probability of infection follows a exponential distribution, are adequate to describe the observed infection trees. They find that the required prob distribution
more closely follows that of a Weibull distribution with alpha > 1. Overall I found the paper interesting and useful for the discussion on modelling viral spread using SIR models which typically

use exponential distributions to model the viral transmission rates. I have a couple of points which Authors should address.

Major

The Authors say that alpha > 1 has a better fit to the data then alpha =1 which would be the exponential distribution. This makes sense as infection rate from an infected individual to a non-infected one is not constant but increases after initial exposure, then falls off as the individual enters the recovery phase. So the source of the non-markov behavior is not that the current state depends explicitly on previous states per se, but more that the probability distribution of transmission is not exponential. There are other models which have been used to model SARS-cov spread which look at additional compartments, e.g. a SIER, or Susceptible, Exposed, Infected and recovered. One could in effect model a changing beta for infection rate by using the additional Exposed compartment to assume at this stage infection rate is not maximal, but has a smaller probability (or larger) prior to infection and recovery. It would be useful for the Authors to answer the questions:

1) To what degree is a SIR model with standard Markov assumptions able to reproduce the infection tree data, i.e. how "bad" is it compared with the non-Markov Weibull case. And equally, how much does the alpha > 1 case improve the fit to the data. (I am not sure this was brought out effectively in the paper).

(R) We thank the Reviewer for this insightful comment. In the manuscript, our method for estimating the shape parameter alpha relies on either least squares (corrected via simulations) or maximum likelihood estimation to ensure we obtain the value that provides the best possible fit to the data under our model. However, we agree that explicitly comparing the fit of the Markovian versus the non-Markovian model helps to highlight the significance of the alpha > 1 case.

To address this, we have added a new subsection in the Supporting Information (sec. S5.1). We computed the Wasserstein distance between the empirical hopcount distribution, and the simulated distributions generated by both the standard Markovian SI model (alpha = 1) and the non-Markovian SI model with Weibull infection times (using the best-fit alpha derived from the data). As shown in the new Fig. S4, the Wasserstein distance for the Markovian process is often 5 to 10 times higher than that of the non-Markovian process.

Furthermore, to test the robustness of our results, we investigated whether incorporating recoveries into our model would alter our conclusions. As shown in the new Fig. S6, a non-Markovian SIR model with Weibull-distributed infection and recovery times requires an even higher alpha to minimize the Wasserstein distance to the empirical distribution. This confirms that the strong non-Markovian signature (alpha > 1) is a robust feature of the data and not an artifact of our SI model assumptions.

We believe these additions substantially strengthen our findings by explicitly quantifying the limitations of the Markovian model, and we are very grateful to the Reviewer for raising this point.

2) To what degree does the addition of more compartments (e.g. the Exposed category) improve the Markov type models fit to the data. I don't expect you to go to great lengths making several more compartments, but seeing the inclusion of say the Exposed compartment and its effects on fitting to the data might be useful.

(R) We are very grateful for this comment, as it allowed us to delve deeper into the nature of the non-Markovianity observed in the data. Adding an "Exposed" (E) compartment introduces an incubation period, which effectively creates an effective non-exponential infection time that can mimic the properties of a Weibull infection time with a shape parameter alpha > 1.

To explore this, we have added a new section in the Supporting Information (S5.1.2 "Markovian SEI vs non-Markovian SI") comparing the fit of our non-Markovian SI process against a Markovian SEI process. By tuning the ratio between the transmission rate and the incubation rate (beta/gamma in our notation), we found that the SEI model can indeed provide a fit comparable to, and occasionally slightly better than, our non-Markovian model (Figure S5 and Table S1). However, achieving this optimal fit often requires a beta/gamma ratio ranging from 0.1 to 1.0, which often implies an incubation rate that is substantially larger than the transmission rate, a scenario that contradicts established clinical timelines for COVID-19 (ref. [8,17,18,42]).

Although the Markovian SEI process may be able to reproduce the hopcount distribution, the inferred physics of the spreading process are unrealistic and lead to misleading conclusions about the duration of the incubation and infection time. In presence of better data about the incubation and infection times we could build a more suitable non-Markovian SEI process which would provide a better fit to the data. In contrast, our non-Markovian SI process with Weibull infection time only models the net effect of the spreading process and it is parameterized by a single shape parameter alpha which is consistently above 1.0 across different waves and tree sizes (Fig. 8).

Thereby, our model allows us to successfully capture the deviations from the Markovianity through the topological properties of the infection trees without requiring rigid assumptions or multiple parameters (e.g. incubation, recovery rates) for unobserved compartments. While we agree that expanding to more general compartmental models is highly valuable for mechanistic modeling, our framework offers a parsimonious alternative for analyzing transmission topology when the exact timelines of various disease stages are poorly known, as in our dataset.

Other than in Supporting Information (Section S5.1.2), we have mentioned this additional results in the "Discussion" section (L535-551).

To sum up, since Markov models are very useful because they are easy to model, it would be

nice to gauge how terrible (or close enough) they are compared with a more accurate distribution (such as Weibull) since these distributions can sometimes be more difficult to sample.

(R) We thank the Reviewer for raising this important final remark. We fully agree that the primary appeal of Markov models lies in their simplicity and analytical tractability. As demonstrated in our supplementary analysis, expanding a Markovian framework by introducing more compartments (e.g., transitioning to an SEI model) can indeed bridge the gap to the empirical data while preserving traditional Markov modeling techniques.

However, in light of our results, particularly the remarkable robustness of the alpha parameter across different epidemic waves and tree sizes, and the rather "unphysical" infection and incubation rates needed to fit the data, we believe that our non-Markovian approach offers a more elegant and insightful alternative. Rather than developing a multi-compartment model to force exponential distributed times, a single robust parameter (alpha) successfully captures the necessary memory effects and transmission delays. This keeps the model highly interpretable while firmly grounding in the empirical tree topology.

Furthermore, regarding the computational concern, the Weibull distribution is natively supported by standard statistical software and is numerically very well-behaved, especially for shape parameters alpha > 1. From a simulation and sampling perspective, employing it to generate infection times does not introduce any meaningful computational bottlenecks or slow-downs compared to standard exponential sampling.

Reviewer #3: The authors analyzed large-scale COVID contact-tracing data from Cyprus and reconstructed infection trees across four epidemic waves before Omicron waves. They assessed the degree of non-Markovianity in transmission using hopcount and outdegree distributions. They further estimated dynamic effective reproduction numbers and compare them with previous model-based estimates.

The work may be more suitable for a mathematical biology journals such as the Journal of Theoretical Biology or Journal of Mathematical Biology. It is not easy to find insights from the computational biology perspective in this specific work.
(R) We appreciate the Reviewer's perspective regarding the excessive theoretical nature of our study. However, we believe our manuscript is a good fit for PLOS Computational Biology, specifically within the Epidemiology & Public Health section. While the foundation of our work is theoretical, its primary objective is to derive epidemiological insights by applying computational methods to real-world contact tracing data provided by the Cyprus Ministry of Health.

Our research moves beyond pure theory by demonstrating, through the structure of measured infection trees, that the actual infection process of COVID-19 is governed by non-Markovian dynamics. We develop a methodology, well-grounded in the theory of stochastic epidemics on networks, which provides evidence that infection time follows a unimodal distribution rather than the memoryless process often assumed in compartmental models. Our findings

offer significant insight into how the virus spreads within a human population and suggest that computational models must account for these dynamics to accurately predict epidemic outcomes.

Additionally, we believe our work demonstrates the value of contact tracing, both in deepening the understanding of biological processes and in improving the monitoring of epidemic evolution.

I have the following suggestions:

(1) It is unclear whether the authors have incorporated under-reporting within the observed infection tree. It would be helpful to the authors could incorporate the uncertainty in case reporting over each wave into the model.

(R) We are grateful to the Reviewer for raising the issue of under-reporting. While we did not explicitly model under-reporting, as such specific metadata was not available from the Ministry of Health, we have addressed this uncertainty through a two-fold approach.

To account for the variability inherent in contact-tracing data, where approximately 25% to 30% of cases were not linked to a specific tree, we have complemented all estimates with non-parametric bootstrap confidence intervals. This ensures that our core conclusion, that COVID-19 dynamics are governed by a non-Markovian process with alpha>1, remains statistically significant despite potential under-reporting. This finding is consistently aligned with recent literature (ref. [37]), regarding the unimodal distribution of generation intervals.

Furthermore, we acknowledge that the effective reproduction number is more sensitive to under-reporting than the shape parameter. Because of the exclusion of isolated positive cases, and due to the fact that our analysis includes smaller trees, which may be sub-components of larger, unobserved chains, our primary calculation of the reproduction number serves as a lower bound (Fig. 9, 10, 11, S9, S10) to the estimates provided by the Cyprus Ministry of Health (MoH). To address this resulting underestimation, we included an alternative analysis both in Main text and in the Supporting Information. By excluding the last level from all infection trees, we provide an estimate that compensate for the under-reporting as shown in Fig. 12 and S11.

(2) The public health and biological implications of 'memory effects' in disease transmission are unclear. It would be better if the authors could link their memory effects findings to the effectiveness of interventions or population behaviour changes. Otherwise, it is not easy to understand why memory effects in disease transmission is helpful to understand real-world public health problems.

(R) We appreciate this comment as it provides an opportunity to clarify how the shift from a Markovian assumption (alpha = 1) to an empirically grounded non-Markovian framework (alpha>1) directly impacts the effectiveness of public health interventions.

By "memory effects," we imply that the probability of transmission depends heavily on the time elapsed since the host was infected. This is a fundamental property, reflecting the natural progression of the transmission probability with the duration of a contact, often discarded in standard Markovian compartmental models for the sake of mathematical simplicity.

A non-Markovian infection process characterized by a Weibull-like distribution with a shape parameter alpha>1 implies a transmission window that is more predictable and concentrated around a mean generation interval. From a public health perspective, this means the timing of contact tracing and isolation is essential; even slight delays in isolating contacts during this peak window can lead to a disproportionate failure in breaking the chain of transmission.

In addition, because the probability of infection is not constant, the non-Markovian dynamics shift the critical threshold required for an epidemic to persist (ref. [19]). Specifically, an epidemic governed by a Weibull distribution with alpha>1 typically requires a higher effective infection rate (i.e. the ratio between the average infection and recovery times) to infect the same fraction of the population compared to a memoryless Markovian process. This suggests that the window for applying non-pharmaceutical interventions may be more nuanced than standard models suggest. Understanding these dynamics helps authorities avoid both premature or delayed lockdowns by identifying the true critical values of transmission.

Reviewer #4: Using detailed contact-tracing data from Cyprus, this paper reconstructs COVID-19 infection trees across the first four epidemic waves to test whether transmission follows Markovian (memoryless) assumptions. The authors show that the data are best explained by non-Markovian dynamics. This indicates that real COVID-19 transmission exhibits memory effects. The study also estimates the effective reproduction number directly from the outdegree (number of secondary infections) in the infection trees over time.

The authors use a complex data system (transmission trees) to demonstrate the non-regularity (non-Markovian process) of the behaviors of living organisms and propose a counting approach for the estimation of the number of reproductions. The basic principle is that the behavior of individuals in life sciences is not regular. The principle of statistical modeling is to identify the common part of this behavior due to factors that everyone shares and to consider the individual parts as accidental variations. Markov approaches are used to assume that, in an homogeneous group, everyone transmits in the same way and individual differences are accidental. If different rates of transmission are estimated, this cannot be an estimate that is not attributable to epidemic behavior. Otherwise, it would be useless. I would like to estimate for example a \alpha value as a function of the stringency index, which would be meaningful. Otherwise, shown as it is, this study is not reproductible and will not find big interest out of this specific Cyprian context.

(R) We thank the Reviewer for this thoughtful comment. We agree that observed transmission patterns reflect both mechanisms common across individuals (e.g., pathogen biology, contact opportunities, and interventions) and individual-level variability. A central goal of our work is

to extract such shared properties of the spreading process from infection-tree structure by comparing the data to a Markovian SI baseline.

In our framework, the parameter alpha does not represent an individual transmission rate, and we do not interpret it as person-specific behavior. Rather, alpha summarizes a fundamental property of the distribution of transmission times (i.e., non-Markovianity of the infection process). The Markovian case (alpha = 1) corresponds to a constant transmission hazard over time, whereas alpha > 1 corresponds to a time-dependent hazard that increases with elapsed time. Importantly, we estimate alpha from topological properties of infection trees and find similar values across multiple waves and across tree sizes (Fig. 8). These findings suggest that alpha captures a robust feature of the transmission-time dynamics rather than accidental individual differences.

We agree that relating alpha to non-pharmaceutical interventions (e.g., a stringency index) is an interesting and potentially meaningful research direction. Based on the consistency of alpha across waves (Fig. 8), with different restriction phases (Fig. S2), we hypothesize that alpha is likely weakly dependent on the stringency index at the level resolved by our data. A more definitive assessment would require additional information to disentangle changes in contact structure from changes in transmission-time dynamics (e.g. time-resolved contact-networks, or a clearer mapping between policy measures and behavior).

Finally, we emphasize that the study is fully reproducible: the tree construction and the statistical methods employed are specified in detail and can be applied to any dataset of reconstructed transmission trees with comparable fields. The Cyprus dataset is unusually detailed, enabling a strong empirical test, but the approach itself is not specific to Cyprus.

Page 4, L37. The concept of "SI" should be defined here, not on page 8.

(R) We thank the Reviewer for the suggestion. We have revised the Introduction to define the SI process (L37-40).

Page 6. 11573 were removed because their parent source could not be found. And for imported cases? How can one identify the roots then?

(R) We appreciate the Reviewer's comment regarding imported cases. Unfortunately, our dataset does not explicitly identify which cases were imported.

In our analysis, any individual who tested positive in Cyprus without a traceable local contact is identified as a root node. This means that in our model both imported cases and local cases with unidentified sources are treated identically as the root of new infection trees.

Page 6. How are the epidemic waves defined? It is confusing that these are not contiguous? Aren't the period between two consecutive waves considered as part of the epidemic period?

(R) We thank the Reviewer for this observation regarding the definition of the epidemic waves. The waves in this study are defined by centering a 31-day analysis window around the peak of each wave, identified by the maximum number of daily positive cases.

The waves used in our analysis are provided in this form by the Cyprus Ministry of Health and correspond to well-validated peak periods during which epidemiological investigations were complete and transmission links could be reliably reconstructed. The periods between consecutive waves were not included in the dataset, as contact-tracing coverage during those intervals was substantially lower and did not support reliable reconstruction of transmission trees.

While we agree that the epidemic is fundamentally a continuous process, having non-contiguous periods helps us for a specific methodological purpose. By studying separated high-activity windows, we can study each wave independently. This allows us to verify whether the identified non-Markovian dynamics and the specific shape of the infection time distribution are robust across different periods, rather than being artifacts of a single point in time.

In view of this comment, we have revised the “Dataset” section (L104-107) and the "Infection trees" section (L117-119).

Page 7. We don't see difference between domestic and imported cases in trees?

(R) We thank the Reviewer for their concern about the difference between domestic and imported cases. As we mentioned in the response to a previous comment, our dataset does not explicitly identify which cases were imported. The transmission trees are then reconstructed based on contacts tracked within the island, without distinguishing domestic and imported cases. Therefore, any individual with no recorded infector is effectively identified as a new root in the system.

In view of this and the previous comment we have added a point in the “Data limitations” section (L159-161).

Page 7. It feels like trees are built in the waves independently. One should be able to draw a trajectory from patient zero to the last infected patient. Here we have isolated trees. Maybe this should be discussed more.

(R) We are grateful to the Reviewer for this insightful comment, and we agree with the concern regarding the assumed independence between infection trees. While it is true that every infection belongs to a single, global infection tree (or multiple ones in the case of imported cases), the presence of isolated trees is expected when working with real-world contact-tracing data, which are inherently sparse and incomplete. Missing links arise from unreported contacts, incomplete recall, or cases that were not successfully traced, and naturally result in fragmented transmission structures rather than a single continuous chain from the first to the last infected individual. As documented in previous works (ref. [7,9,33]), incomplete information is common in such datasets.

In the absence of granular information regarding the universal contact network or the precise paths connecting disparate clusters, we adopt a conservative framework that avoids the introduction of speculative links. The assumption of independence is a standard and necessary framework for large-population modeling, particularly when the it is unknown how each infection tree belongs to the global "shortest path" tree of the entire epidemic. This approach allows for a rigorous theoretical and simulation-based analysis while avoiding the risk of over-interpreting the data. To ensure our conclusions remain robust despite the high variability inherent in such measurements, we employ bootstrap techniques to calculate confidence intervals, providing a characterization of the infection process that is firmly grounded in empirical evidence.

In view of this extremely important comment, we have revised the “Infection trees” section (L122-126) and the "Discussion" section (L554-558).

Page 7. The authors did talk about public health measures and analyzed stringency indexes in the supplementary material. How are these taken into account in modelling?

(R) We thank the Reviewer for mentioning the role of public health measures in our work, as we think it’s an important point to address. While we display the Stringency Index in the Supporting material (Fig. S2) to offer context on the epidemic's environment, it is not explicitly incorporated into the modeling of the shape parameter.

We have two reasons for this choice. First, significant variations in the Stringency Index were primarily observed during the initial phase of Wave 1, while the indices remained relatively stable across the other analyzed waves (Fig. S2). We believe that public health restrictions primarily influence the topology of the local contact graph (e.g., disconnecting workplace contacts while leaving household contacts intact) rather than the intrinsic distribution of transmission times. Since our estimate of the shape parameter alpha characterizes the non-Markovian dynamics of the transmission process itself, it should remain robust to changes in the number or type of available contacts.

Second, quantifying the precise impact of stringency index variations on the topology of the infection trees is highly complex and would require making assumptions about the dynamics of the underlying contact graph, which remains unknown. In the absence of granular information regarding this underlying graph, any attempt to model how policy shifts alter the measured tree structure would move beyond the empirical evidence provided by the contact-tracing data. The assumption of a local complete graph is indeed made in view of the limited information available, with the scope of avoiding the introduction of speculative biases that could distort our characterization of the infection dynamics.

While the reproduction number reflects these policy changes more directly, as seen in the prevalence decrease following the stringency index increase in Wave 1, our focus remains on the temporal characteristics of the transmission. By relying on the recorded infection trees, we maintain a conservative estimate of the infection dynamics that is robust to the structural uncertainties of the underlying network.

Page 13. How does the model take into account the period of infectivity? As well defined, the reproductive number estimate the infections in a given period. Is that considered somewhere in the estimation?

(R) We thank the Reviewer for this comment. In our study, the exact duration of the infectious period is not explicitly modeled, because these quantities are not reported in the contact-tracing trees (we only have the date at which an individual tested positive and its inferred transmission links). Our effective reproduction number is therefore defined as a tree-based reproduction number: it is the mean outdegree of individuals whose positive-test date falls within the window $W_{\Delta t} = (t - \Delta t, t]$. The time window is used to group primary cases by their reporting date and it does not imply that secondary infections positively tested within the same calendar window. Consistently with this, at the end of the "Effective reproduction number from the evolution of the outdegree distribution" section (L386-389), we note that our estimate is not intended for real-time monitoring, because secondary infections linked to cases in $W_{\Delta t}$ may occur after the window closes. A real 'instantaneous' reproduction number would require additional assumptions and data (generation-interval/infectiousness kernel and delay/censoring corrections) that are not available for our dataset.

Page 13 L263. The definition of the effective reproduction number should be reconsidered. It is not the estimate of the basic reproduction number.

(R) We thank the Reviewer for pointing out this terminological inconsistency. We agree that the value estimated from the infection trees represents the effective reproduction number, which is often smaller than the value of the basic reproduction number, and it reflects the impact of control measures and depletion of susceptible people during the epidemic. We have revised the manuscript (L304-311) to consistently refer to our estimate as the effective reproduction number and have removed references to the basic reproduction number in the context of our empirical measurements to ensure technical accuracy. Additionally, we have rephrased our definition of the effective reproduction number to highlight its dynamical nature which is crucial in the context of the infection trees.

Page 14. It cannot be assumed that all trees have the same length N. The trees are supposed random, with random number of leaves. Explain how these can be considered with fixed length.

(R) We thank the Reviewer for raising this important point. We agree that, in general, infection trees are random objects, and their size (and number of leaves) is stochastic. Importantly, in our work we do not assume that all empirical trees have the same size. In our analysis, N denotes the observed number of nodes in a given reconstructed tree. Because the dataset contains trees of many different sizes (as explained in Sec. "Infection Trees"), we proceed by conditioning on tree size: for each integer N we form an ensemble consisting of all empirical trees with exactly N nodes, and we compute the corresponding empirical hopcount (and outdegree) distribution within that ensemble. We then fit our model to each

conditional distribution separately. Therefore, when we fix N we are not doing it for the overall dataset, but only for a collection of trees of that recorded size.

To compare with theory, we model each empirical tree of size N as an independent realization of a local spreading process on a fully connected graph with Weibull infection times. This assumption is a choice motivated by the limitations of contact-tracing reconstructions: the underlying contact network and missing transmission links are not observed, and introducing a model for missing links would require additional assumptions. Conditioning on N allows us to: (i) use the reconstructed trees directly, (ii) obtain a tractable theoretical hopcount benchmark for each size, and (iii) estimate the non-Markovianity parameter alpha in a way that is comparable across tree sizes.

Finally, we explicitly check whether the inferred alpha depends on N. As shown in Fig. 8, the estimates of alpha are consistently greater than 1 across the range of available tree sizes, supporting the interpretation that the non-Markovian signature is not an artifact of selecting a particular tree size but a robust feature of the empirical spreading dynamics.

Page 15. We have the estimate \alpha, what about \beta?
(R) We thank the Reviewer for this question. In our Weibull model, alpha controls the shape (non-Markovianity), whereas beta is a rate parameter that sets the overall time unit. Our inference is based only on the topology of the reconstructed infection trees (hopcount and outdegree distributions), which depends on the relative ordering of infection times along links rather than on their absolute durations. Consequently, a global rescaling of all link infection times does not change the infection tree structure: changing beta is equivalent to multiply all link weights by a constant factor, which leaves the hopcount and the tree topology invariant.

Therefore, beta is not identifiable from tree topology alone, and we set beta = 1 without loss of generality. Estimating beta would require additional temporal information beyond the transmission ordering (e.g., generation/serial intervals) and/or individual-level data (demographic/clinical/behavioral) to model heterogeneity in infectiousness or contact intensity. Since the infection trees contain only case identifiers and infector identifiers, we focus on the identifiable parameter alpha, which governs the non-Markovian signature and can be inferred from the infection-tree structure.

Finally, introducing heterogeneous infection rates across links would add extra degrees of freedom that cannot be reliably estimated from topology-only observations. We therefore treat infection rates as approximately homogeneous within each local ”community” (i.e., within a single small transmission cluster), which we consider a reasonable first-order approximation given the limited information available and the localized nature of the reconstructed trees.

In view of this precious comment, we have revised the "Discussion" section to explain more in detail the choice of beta. (L561-563)

Page 16. Finally, the reproduction numbers are determined by counting. So I wonder about the usefulness of the whole modeling approach. The Markovian assumption was rejected, but no other modeling approach is proposed. Then the method cannot be reproduced elsewhere.

(R) We thank the Reviewer for raising these concerns. Our modeling framework is not primarily introduced to compute the reproduction number, but to test (and quantify) whether the observed transmission trees are compatible with a Markovian, memoryless spreading mechanism. Using the equivalence between SI infection trees and shortest-path trees, we define a minimal non-Markovian SI baseline with Weibull infection times, where the shape parameter alpha captures departures from the Markovian case (alpha = 1). This yields a reproducible estimate of non-Markovianity from tree-structure statistics (hopcount and root outdegree) that can be compared across tree sizes and waves. If the underlying contact network (and ideally its temporal dynamics) were known, the inferred non-Markovian infection-time distribution could then be incorporated into a network-based non-Markovian epidemic model to forecast the epidemic evolution.

Regarding the effective reproduction number, we agree it is based on counting secondary infections (outdegrees), which is the standard estimator when transmission trees are available. Here it mainly provides a transparent comparison with prior estimates. The bootstrap confidence intervals allow to take into account both the uncertainty in the contact tracing and the variability of the spreading dynamics; while the explicit treatment of biases due to incomplete trees with pruning techniques is employed to tackle the selection bias of the data.

Finally, the overall workflow is reproducible elsewhere because it requires only reconstructed transmission trees (who-infected-whom) and case dates, without reliance on population-specific compartmental assumptions.

Page17 L349. Fig. is used 2 times in the reference

(R) Thanks for noticing this typo, we have corrected it.

Reviewer #5: This manuscript constructs infection trees for the first four waves of the COVID-19 epidemic based on contact tracing data provided by the Cyprus Ministry of Health, revealing the non-Markovian dynamics of epidemic transmission. Furthermore, the authors estimate the effective reproduction number using the structure of infection trees and compare it with official model results. The research has strong practical significance and academic value, with a reasonable methodological design and complete content. However, some concepts and conclusions require further explanation and clarification. Specific suggestions are as follows:

1. Regarding the explanation of "hopcount": It is recommended to illustrate the concept of "hopcount" with examples to help readers intuitively understand its meaning within infection trees.

(R) We thank the Reviewer for this comment. We agree that the hopcount is better illustrated with the help of an example from the dataset. We have revised the “Infection trees” section (L146-150) and we have added an example of an infection tree (Fig. 2) from the dataset.

2. Regarding the explanation of research innovation: There is currently a consensus that non-Markovian models, characterized by non-exponentially distributed infection and recovery times, can more realistically describe epidemic transmission processes. Please further clarify which conclusions this paper has drawn that were not explicitly identified or quantified in previous studies.

(R) We are very grateful for raising this concern. We agree that the use of non-Markovian epidemic models is by now well established, and our main innovation is indeed not simply in the fit of empirical data with the help of non-exponential distributions.

The specific conclusion that, to our knowledge, has not been explicitly identified or quantified in previous studies is that non-Markovianity can be reliably inferred from topology alone in real contact-tracing infection trees. In our dataset, reliable generation-interval information is not available at the level needed to directly fit time distributions. Nevertheless, by exploiting the equivalence between SI infection trees and shortest-path trees, we show that the empirical hopcount distribution carries enough information to estimate the Weibull shape parameter alpha, i.e. to detect non-Markovian effects in the underlying transmission process without knowing inter-event times.

Importantly, this is not a fragile, single-wave observation: the inferred values consistently satisfy alpha > 1 across multiple epidemic waves and across different tree sizes, and the estimates remain stable under bootstrap resampling. This robustness supports a stronger, more practical conclusion: even in the absence of complete temporal data, the hierarchical structure produced by contact tracing contains information about the nature of transmission dynamics and can be used to quantitatively reject the Markovian hypothesis in a statistically principled way.

In view of this important point, we have revised the Abstract and Conclusion to make more explicit the main innovation of our paper.

3. Regarding the definition of "infection times": It is recommended to clarify the specific definition of "infection times," for example, whether it refers to the interval from exposure to becoming infectious, or the time from infection occurrence to being recorded.

(R) We thank the Reviewer for highlighting this important point, as precise terminology is crucial for interpreting our modeling framework.

In the theoretical context of our network SI model, the "infection time" is defined as the time needed for a just infected node to infect one of its direct, susceptible neighbors. Because the epidemic spreads through competing processes across the contact graph, a susceptible individual is ultimately infected by the neighbor whose drawn infection time is the shortest.

In practical terms, our infection time does not exclusively represent a single biological phase (such as the strict interval from exposure to infectiousness). Rather, it acts as an effective transmission time that inherently captures a convolution of multiple, often unobservable, compounding delays. These delays could span from true biological latency and incubation periods to human behavioral factors, such as symptom reporting delays, testing lags, and the time taken to isolate.

Furthermore, a recent study (ref. [37]) analytically proved that utilizing a Weibull infection time with a shape parameter alpha>1 defined on network links, as we do in our framework, leads to an effective generation interval (the time from the infection of the infector to the infection of the infectee) that exhibits a qualitatively similar unimodal distribution, which aligns with empirical evidence for COVID-19 (ref. [8,17,18,42]).

To ensure this concept is absolutely clear to the readers, we have updated the manuscript to explicitly include these definitions. Specifically, we have added explanatory text detailing the exact nature of the infection time to the "Introduction" section (L42-43), as well as to the "Markovian SI infection tree and shortest path tree with exponential link weights" section (L187-191).

4. Regarding the explanation of model equivalence: The manuscript states: "We employ the equivalence between the non-Markovian SI process in a contact graph and the shortest path problem in a graph: an infection tree is equivalent to a shortest path tree if infection times correspond to link weights." It is suggested to further explain the theoretical basis and applicable conditions of this equivalence to enhance understanding for non-specialist readers.

(R) We are very grateful to the Reviewer for this comments as it allows us to explain better the theoretical foundations of our model already in the “Introduction”. The equivalence follows by viewing the transmission from the root to any node as finding the shortest path between them: assigning an independent infection time to each contact, the time at which an individual becomes infected is the minimum total infection time over all paths from the root, i.e. a shortest-path distance. Under the SI assumptions (no recovery/reinfection), the resulting parent-child relations form the corresponding shortest-path tree.

In view of this comments, in the “Infection trees as shortest path trees” (L175-180) we have added a more detailed explanation.

5. Regarding the handling of isolated cases: Please explain how isolated cases with an unidentifiable source of infection were handled when constructing the infection trees, and the potential impact of this handling on subsequent analyses.

(R) We are deeply grateful to the Reviewer for asking about the potential consequences of the selection bias inherent in our dataset. Not having any reliable information to handle isolated cases and to maintain the integrity of the contact-tracing evidence, we have opted to stick strictly to the confirmed links, therefore neglecting the isolated nodes.

For the estimation of the non-Markovianity parameter alpha, this exclusion means that our inference is conditional on the subset of cases belonging to reconstructed transmission chains.

In principle, missing links can bias the observed hopcount distribution in either direction (increasing or decreasing alpha) depending on where links are missing (e.g., missing branches close to the root versus missing terminal branches), and therefore the direction of the bias on alpha cannot be determined a priori. Our results are thus conditional on this intrinsic limitation, which we explicitly acknowledge in the "Discussion" section.

Regarding the effective reproduction number, this choice leads to a systematic underestimation. Indeed, as illustrated in Figures 9, 10, and 11, our estimates are often lower than the official estimates provided by the Cyprus Ministry of Health (MoH). The only exceptions coincide with periods of high variance in the daily outdegree distribution, which can be explained by "superspreading" events.

To address the resulting underestimation, we have proposed to remove the final generation (leaves) from all infection trees. Fig. 12 shows that this approach mitigates the underestimation of the effective reproduction number and provides estimates which reflect more faithfully the evolution of the epidemic spreading. Indeed, since our dataset covers the unfolding of four epidemic waves, for each of them the effective reproduction number is expected to decrease progressively from before to after the peak. The unpruned estimates fail to capture this behaviour (Fig. 12 left), showing little variation or even an increase after the peak. By contrast, the pruned estimates consistently show the expected decreasing trend across all four waves (Fig. 12 right). Besides, in Fig. S11 we show that pruning of the infection trees may often provide an upper bound for the reproduction number compared to the Cyprus MoH reference values (especially for waves 3 and 4 where more data is available).

We acknowledge, however, that missing links due to isolated cases may more broadly affect the structural properties of the reconstructed trees, and quantifying this effect rigorously remains an open challenge. This is currently an active line of research for some of the authors of the manuscript, who are developing link-prediction algorithms to infer plausible unobserved links. Once validated, these methods will allow to systematically assess the resulting bias.

6. Regarding the variation in the number of infection trees across epidemic waves: The number of infection trees varies considerably across the four waves. Please analyze the possible reasons for this phenomenon (e.g., changes in transmission patterns, impact of control policies, data recording methods).

(R) We thank the Reviewer for this observation. Indeed, the variation in the number of infection trees across the four waves is driven by multiple concurrent factors: (I) the epidemic started with wave 1 and then progressively intensified over the following months, culminating in waves 3 and 4 with orders of magnitude more reported infections than waves 1 and 2 (Table 1); as a consequence, many more cases with a traceable infector were identified in the later waves, naturally increasing the number of reconstructed transmission chains/trees; (II) as shown by the evolution of daily tests (Fig. S1), testing volume increased substantially in waves 3 and 4 with widespread rapid antigen testing, leading to higher case

detection and to the identification of more infector–infectee links, which in turn increased the number of observed trees. (III) During Wave 3 the national surveillance system transitioned from telephone-based interviews to electronic documentation, which may have affected the completeness and consistency of contact-tracing records. While changes in interventions (stringency index) can in principle affect transmission patterns and fragmentation into multiple chains, in our data this effect is not straightforward to isolate from the large temporal increase in testing and prevalence, which likely dominates the observed variation in the number of reconstructed trees.

In view of this comment, we have revised the “Infection trees” section to explain the possible reasons for the variation in the number of infection trees across epidemic waves (L131-139).

7. Regarding the impact of interventions: In the presence of non-pharmaceutical interventions, how can one distinguish between the basic reproduction number under natural transmission and the effective reproduction number under controlled conditions? It is recommended to clarify this point.

(R) We thank the Reviewer for raising this important point. To clarify, our methodology inherently measures the effective reproduction number, rather than the basic reproduction number. Because our analysis relies on empirically reconstructed infection trees from contact tracing data, the number of secondary infections we measure (the outdegree of the nodes) already reflects the impact of the non-pharmaceutical interventions (NPIs) that were in place at the time.

As detailed in the Supporting Information (and shown in Fig. S2), Cyprus implemented strict non-pharmaceutical interventions very early in the pandemic, with the stringency index reaching ~85/100 during the first wave and remaining relatively high (between roughly 47/100 and 75/100) throughout the subsequent waves. Because there was virtually no period of unmitigated, "natural" transmission in our dataset, it is not possible to isolate the theoretical basic reproduction number using these infection trees. Therefore, our study focuses solely on the effective reproduction number, which captures the actual transmission dynamics under these controlled conditions.

We agree that this distinction is crucial, and we have added a brief clarifying statement in the manuscript “Effective reproduction number from the evolution in time of the outdegree distribution” section (L390-393) to ensure readers understand that our estimates reflect transmission under the presence of these recorded interventions.

8. Regarding the impact of testing and confirmation time: The actual reported time of secondary cases depends not only on the infection time but also on delays in testing and confirmation. Has this factor been considered or discussed in the manuscript? Could the testing and confirmation time affect the estimation of relevant distributions?

R) We are very grateful for this insightful question. The Reviewer raises a highly relevant point regarding the exact nature of the timelines captured in epidemiological data.

In short, yes, the observed testing and confirmation times may affect the topological progression of the reconstructed infection trees. As detailed in our previous responses, the empirical trees may not be affected only by the biological transmission. Instead, they are affected by both biological generation intervals and administrative/behavioral delays, including the precise testing and confirmation lags the Reviewer mentions.

Because it is exceptionally difficult to fully decouple these different delays, especially in absence of precise information in our data, we avoid making rigid assumptions about unobserved events, and we simplify the model adopting a conservative approach. The "infection time" in our model acts as an effective transmission time. By utilizing a Weibull distribution with a shape parameter $\alpha > 1$, our framework systematically absorbs all of these real-world delays into a single parameter that governs the entire transmission attempt.

Regarding the effective reproduction number estimates, our approach groups infection trees into aggregated time windows based on the date individuals were tested positive. Because nodes in the dataset are recorded as infected based on their test date, any delays in testing and confirmation are inherently embedded within the chronological progression of the trees. Consequently, these administrative delays are implicitly reflected in our temporal estimates of the effective reproduction number. As with the infection time discussed above, our technique does not attempt to explicitly quantify or decouple these testing delays from the biological transmission intervals. Instead, the estimated reproduction numbers capture the combined, real-world dynamics of both the disease spread and the contact tracing process as they unfolded during each epidemic wave.

9. Regarding sample selection in the Results section: At the beginning of the Results section, it is recommended to explain why N = 5, 6, 7, 8 were selected for analysis. Additionally, why does Figure 7 only show results for waves 1, 3, and 4? Please provide a supplementary explanation.

(R) We thank the Reviewer for these insightful questions regarding our sample selection and Figure presentation in the Results section. We have decided to display the hopcount distribution for trees of sizes 5,6,7,8 in wave 4 because they represent the groups with the highest volume of reconstructed trees in a single wave. By focusing on these specific samples, we visualize the distributions where the noise should play the smallest role. For transparency and reproducibility, the code provided in our repository allows for the plotting of these distributions for any other tree size or wave where data is sufficient to build a hopcount distribution. We have added a clarification in Sec. "Infection trees show non-Markovian properties" (L397-398).

On the other hand, the new Figure 8 omits Wave 2 because the frequency of reconstructed trees of size N>4 was insufficient to build a reliable hopcount distribution. We have added clarifying note in the text following Figure 7 to explicitly state this choice (L413-417).

10. Regarding the readability of data in Figure 7: Please explain more clearly in the figure legend or the main text the meaning of each data point in Figure 7, such as the specific values

represented by the size and color of the points (e.g., 3, 24, 240) and their statistical significance.

(R) We are very grateful for raising concerns about the readability of the figure presenting the results across different waves and tree sizes. We have thus decided to revise the picture (now Fig. 8) removing the “bubbles” and indicating with a label on top of each estimate how many infection trees of size N were employed to estimate alpha in each wave. The number of trees “n” employed are the same for both methods (green and red points). We believe that these changes improve the readability and the clarity of the figure.

11. Regarding the basis for the pruning analysis conclusion: The manuscript states: "From the results in Fig.11 the estimate without the last level is the one which can reasonably reflect the evolution of the epidemic wave." How is it determined that this estimate is more "reasonable"? Is there a true reproduction number available as a reference?

(R) We thank the Reviewer for this valuable question. As noted in our response to a previous comment, Fig. 12 (formerly Fig. 11) demonstrates that pruning the last level of the trees provides estimates of the effective reproduction number that more faithfully reflect the known progression of an epidemic. While a "true" reproduction number is not available as an absolute reference, our assessment of what is "reasonable" is based on established epidemiological expectations: during the unfolding of a single epidemic wave, the effective reproduction number is expected to decrease progressively as it moves from the pre-peak phase to the post-peak phase.

The unpruned estimates fail to capture this characteristic behavior (Fig. 12, left). In several instances, specifically during waves 1 and 2, the unpruned data actually show an increase in the reproduction number after the peak has occurred. In contrast, the pruned estimates consistently demonstrate the expected decreasing trend across all four waves (Fig. 12, right), aligning more closely with the underlying dynamics of the transmission process.

12. Regarding the clarity of method comparison: The \alpha estimates obtained by the two methods (based on hopcount distribution and root outdegree distribution) show some differences (as shown in Figure 7). It is recommended to add a comparative analysis of the statistical power and robustness of the two methods, especially their performance when data is sparse, and to explain why the confidence intervals for one method are wider in certain cases.

(R) We thank the Reviewer for this helpful suggestion. We have added a dedicated comparative analysis of the two estimators in the Supporting Information (Section S4.3; Fig S3), quantifying how their uncertainty depends on data sparsity (n_trees) and tree size (N). Specifically, for each triplet (N, n_trees, alpha) we generated N_boot = 1000 independent synthetic datasets (each consisting of n_trees infection trees of size N generated by the non-Markovian SI process with Weibull infection times) and applied both methods to estimate alpha, reporting the mean and the 5th and 95th percentiles of the 1000 samples distribution.

As expected, Fig S3 shows that confidence intervals narrow as N and n_trees increase, consistent with the fact that more information improves identifiability of the underlying alpha. We also observe that for intermediate non-Markovianity (e.g., alpha from around 2), even sparse datasets of small trees can already reject the Markovian hypothesis with p-val of 0.05. Overall, the two methods exhibit comparable statistical power and robustness across the explored parameter space.

Consequently, the differences between the empirical confidence intervals observed in the main-text (e.g., Fig 8) are primarily driven by the intrinsic variability and heterogeneity of real-world contact-tracing data (finite-size effects, sampling variability across tree-sizes, and reconstruction noise), rather than by a systematic loss of power of either estimator. When modest differences in interval width appear, they can be attributed to the different summaries used by the two approaches (full hopcount distribution versus root outdegree), which respond differently to finite-sample variability in sparse regimes.

We have modified the manuscript to explicitly point to this comparative analysis both in the "Estimate of non-Markovian dynamics from infection trees" section (L364-368) and in the "Infection trees show non-Markovian properties" section (L434-442).

Reviewer #6: The article describes methods for reconstruction of infection trees from contract-tracing data and their use for estimation of epidemiological parameters (e.g., the reproduction number) and for accessing whether the epidemiological process is Markovian. Authors apply these methods to very rare and complete data from COVID-19 epidemic in Cyprus, and show that the process is non-Markovian, and that their method allows estimating a lower bound for the reproduction number. This is indeed a very rare dataset, which makes very interesting analyses possible.

I see three points that need to be considered or explained in the article:

1. Authors found out that the process is non-Markovian but Weibull. The process they consider is SI. However, COVID-19 features an incubation period, which makes it SEIR. One can model incubation with an exponential distribution and then infectious period with another exponential distribution. The sum on both will not be an exponential distribution and can explain the non-Markovian part. I think it is important to assess/discuss the impact of incubation here.

(R) We are very grateful for this comment, as it allowed us to delve deeper into the nature of the non-Markovianity observed in the data. Adding an "Exposed" (E) compartment introduces an incubation period, which effectively creates an effective non-exponential infection time that can mimic the properties of a Weibull infection time with a shape parameter alpha > 1.

To explore this, we have added a new section in the Supporting Information (S5.1.2 "Markovian SEI vs non-Markovian SI") comparing the fit of our non-Markovian SI process

against a Markovian SEI process. By tuning the ratio between the transmission rate and the incubation rate (beta/gamma in our notation), we found that the SEI model can indeed provide a fit comparable to, and occasionally slightly better than, our non-Markovian model (Figure S5 and Table S1). However, achieving this optimal fit often requires a beta/gamma ratio ranging from 0.1 to 1.0, which often implies an incubation rate that is substantially larger than the transmission rate, a scenario that contradicts established clinical timelines for COVID-19 (ref. [8,17,18,42]).

Although the Markovian SEI process may be able to reproduce the hopcount distribution, the inferred physics of the spreading process are unrealistic and lead to misleading conclusions about the duration of the incubation and infection time. In presence of better data about the incubation and infection times we could build a more suitable non-Markovian SEI process which would provide a better fit to the data. In contrast, our non-Markovian SI process with Weibull infection time only models the net effect of the spreading process and it is parameterized by a single shape parameter alpha which is consistently above 1.0 across different waves and tree sizes (Fig. 8).

Thereby, our non-Markovian model allows us to successfully capture the "memory effects" and the topological signature of the epidemic without requiring rigid assumptions or multiple parameters for unobserved compartments. While we agree that expanding to more general compartmental models is highly valuable for mechanistic modeling, our framework offers a parsimonious alternative for analyzing transmission topology when the exact timelines of various disease stages are poorly known, as in our dataset.

Other than in Supporting Information (Section S5.1.2), we have mentioned this additional results in the “Discussion” section (L535-551).

2. It was not fully clear to me how the authors deal with the ongoing epidemic. Do they assume that they have all the population and the epidemic has stopped? (Assumption 2?) How realistic is that? If not, then there is a problem of censoring: one cannot just count the outdegree of nodes (e.g., lines 266-268) as some of the nodes might still be infectious and might increase their degree in the future. This needs to be properly accounted for (e.g., with survival analysis). The same issue arises when picking the values of delta t for the reproduction number estimation (as the authors state in lines 331-335). The choice of delta t values should be further explained.

(R) We thank the Reviewer for raising these important points regarding data censoring and the ongoing nature of the epidemic.

Regarding the assumption of an ongoing epidemic, it is important to clarify that our study is strictly retrospective, analyzing historical data from closed and distinct epidemiological waves (March 2020 – May 2021).

For each wave, the dataset is comprised of reconstructed transmission trees initiated by positive tests within a selected 31-day window. These trees are not artificially truncated; even if secondary infections occurred outside the initial timeframe, they are fully captured.

Consequently, each dataset represents a closed and exhaustive record of the wave, encompassing every registered individual linked to those transmission chains. Because no further cases related to these trees were recorded after the observation period, the data is considered complete (conditioned on the inherently partial information of the contact tracing data), with no possibility of additional cases emerging.

Because our methodology is strictly topological, we apply a topological equivalent to correct for any remaining censoring due to incomplete data: pruning the terminal leaves (the last level) of the infection trees. As demonstrated in our analysis (Fig. 12, S11), excluding the last level successfully increases the outdegree removing nodes that have no offspring because their secondary infections were unrecorded.

Regarding the choice of delta t for estimating the effective reproduction number, our selection of delta t = 1 day and delta t = 1 week was not arbitrary. These specific temporal windows were chosen to allow for direct numerical benchmarking against the official epidemiological reports published by ref. [42], which calculated reproduction numbers on daily and weekly bases.

Finally, as explicitly mentioned in the manuscript, our definition of the dynamic effective reproduction number is not designed for real-time monitoring. Its computation relies on complete retrospective information about secondary cases, including infections recorded after the window has closed. Computing a true instantaneous reproduction number would require precise data on individual infectivity periods and contact networks, which would necessitate additional assumptions that cannot be reliably made with our empirical dataset. We have specified these points in “Effective reproduction number from the evolution of the outdegree distribution" (L386-389).

3. The choice of treating different trees independently instead of considering that there are missing links between them is another assumption whose consequences should be further discussed. What does it mean from a practical point of view? Different independent introductions to Cyprus, is it realistic?

(R) We are grateful to the Reviewer for this insightful comment, and we agree with the concern regarding the assumed independence between infection trees. While it is true that every infection belongs to a single, global infection tree (or multiple ones in the case of imported cases), our methodology adheres to the empirical evidence of the contact-tracing data. In the absence of granular information regarding the universal contact network or the precise paths connecting different clusters, we adopt a conservative framework that avoids the introduction of speculative links.

From a practical point of view, treating the infection trees independently means that we are analyzing the epidemic as a collection of localized, verified transmission clusters. This means that our calculated metrics, such as the non-Markovian parameter alpha and the effective reproduction number, measure the local spreading dynamics (how the virus propagates within a verified cluster).

In practice, this is a conservative approach. It ensures that our estimates are built only on highly reliable, directly observed transmission events, avoiding the compounding errors that would naturally arise if we attempted to guess unverified connections between different clusters.

Another consideration to take into account is that there is a whole field of phylodynamics dealing with SIR-like models on transmission trees (see doi:10.1016/j.jtbi.2010.09.010 for example). If there are sampling and infection times available in the contact-tracing data from Cyprus, one could transform them into time-scaled transmission trees and apply phylodynamic models.

(R) We are grateful to the Reviewer for mentioning the field of phylodynamics and its connection with our work. We agree that in presence of reliable information about sampling, infection and recovery times we could apply birth-death models to our infection tree data. Additionally, as explained in Ypma et al "Relating phylogenetic trees to transmission trees of infectious disease outbreaks", if genetic sequences of sampled pathogens were available, we could also combine infection and phylogenetic trees to infer key epidemiological parameters (e.g. infection time distribution, reproduction number) in a more reliable way.

Unfortunately, the contact-tracing dataset provided for this study lacks the required temporal and genetic granularity. While we possess the dates of positive tests (sampling times), we do not have precise, verified records of the exact infection times, symptom onsets, recovery times, or viral genomes. Transforming our current data into time-scaled transmission trees would require imputing these missing temporal intervals, which would introduce strong, speculative assumptions and potential biases into our framework. Consequently, for our measures (i.e. alpha and effective reproduction umber), we relied exclusively on the measured transmission links.

Nevertheless, we completely agree that integrating phylodynamic modeling with extensive contact-tracing datasets represents the gold standard for future epidemiological surveillance and note this as a highly promising direction for future research when more granular temporal and genomic data will be available.

Minor:
Line 37: SI process is mentioned for the first time. Introduce the acronym please.
(R) We thank the Reviewer for this comment, we have updated the manuscript accordingly.

Fig. 1: It would be helpful to layout the zoomed-in trees without intersections in their branches.
(R) We are grateful to the Reviewer for this comment. We have modified Figure 1 to show the zoomed-in trees without intersections in their branches.

Line 212 and elsewhere: Please put quotes around the section name as otherwise it is not clear where the name of the section finishes.

(R) We thank the Reviewer for this comment. We have modified the cross reference to make the Section names stand out more clearly.

We hope these answers are satisfactory to the Editor and the Reviewers; we remain available for any further clarification you might deem necessary.

Best regards,

Matteo D'Alessandro, Pavlos Alexandros Dimitriou, Brian L. Chang, Valentinos Silvestros, Elisavet Constantinou, Costas Pitris, Panayiotis Kolios and Piet Van Mieghem
