# Supplementary material for: Non-Markovian dynamics and effective reproduction number in COVID-19: Evidence from Cyprus contact tracing data
Source: PLoS Comput Biol. 2026 Aug 21;22(8):e1014578. doi: 10.1371/journal.pcbi.1014578 (PMC13496441; doi:10.1371/journal.pcbi.1014578)
Supplement: Supplementary file 3 [file pcbi.1014578.s020.pdf]

29th June 2026

Dear Editor, Dear Reviewers,

First, we would like to express our gratitude to the original six reviewers. We were very pleased to see that our extensive revisions in the first round successfully addressed all of their concerns, and we appreciate the time they dedicated to our work.

We also thank the newly assigned Reviewer #7 and #8 for their time and feedback. In accordance with the Editorial Board's guidance, we have provided a detailed, point-by-point response (**(R)** in red below) to address the reviewer's comments.

After careful consideration, we believe the manuscript in its current form successfully captures the necessary scope and methodology of our study; therefore, the core text remains unchanged. In the responses below, we highlight where the requested clarifications are already integrated into the current version. Furthermore, we clarify why the remaining suggestions represent distinct, valuable research directions; some of which have already begun or will be tackled in future work, as indicated by two newly added references (ref. [47] and [48]).

Editor Comments:

1) In the online submission form, you indicated that "Daily COVID-19 case numbers are publicly accessible at https://www.data.gov.cy/. Detailed infection trees can be made available upon request to the Ministry of Health of the Republic of Cyprus https://www.gov.cy/moh/en/contact/)". All PLOS journals now require all data underlying the findings described in their manuscript to be freely available to other researchers, either

1. In a public repository

2. Within the manuscript itself

3. Uploaded as supplementary information.

This policy applies to all data except where public deposition would breach compliance with the protocol approved by your research ethics board. If your data cannot be made publicly available for ethical or legal reasons (e.g., public availability would compromise patient privacy), please explain your reasons by return email and your exemption request will be escalated to the editor for approval. Your exemption request will be handled independently and will not hold up the peer review process, but will need to be resolved should your manuscript be accepted for publication. One of the Editorial team will then be in touch if there are any issues.

We have updated the public repository such that all data underlying the findings described in the manuscript are available (https://shorturl.at/ImdjX).

2) Thank you for including an Ethics Statement for your study. Please include:

i) A statement that formal consent was obtained (must state whether verbal/written) OR the reason consent was not obtained (e.g. anonymity). NOTE: If child participants, the statement must declare that formal consent was obtained from the parent/guardian.].

Thank you for this comment. We have revised the Ethics Statement.

Reviewers' comments:

Reviewer #7: This manuscript uses Cyprus contact-tracing data and constructs infection trees for four COVID-19 waves. Then it compares hopcount and root-outdegree summaries with Weibull SI simulations on complete graphs. The dataset is valuable, and the connection to shortest-path tree theory is interesting. However, I think the main claims are stronger than what the inference setting can support. The estimator for α depends on the complete-graph SI assumption, the strong assumption on completed infection trees, and the selection in contact-tracing data. Also, the part on the effective reproduction number depends on a heuristic pruning rule. Therefore, I recommend major revision.

The abstract states that non-Markovianity can be detected only from the topology of infection trees. However, the inference is conditional on Assumption 1 in the Modeling assumptions section, namely Weibull SI on a complete graph K_N. On the other hand, for example, Ogura and Preciado [1] already study non-Markovian transmission and recovery on networks with richer timing structure. Therefore, the authors should rewrite the main claim as a fit-based statement under this latent model, not as a topology-only detection result.
(R) We thank the reviewer for raising this important conceptual point. We fully agree that our findings are conditional on our modeling framework, which is precisely why we explicitly outline these conditions and their implications in both the "Modeling assumptions" and "Discussion" sections of the manuscript.

To clarify our terminology, when we state that "non-Markovianity can be detected solely from the topology of the infection trees" we specifically mean that our inference procedure does not rely on any exact timelines between epidemiological events (such as explicit timestamps of exposure, onset, or reporting). Although the empirical tree topology itself is derived from epidemiological reconstruction that incorporates temporal information, the inference method does not require explicit chronological data beyond the reconstructed network structure.

Given the absence of highly granular empirical data regarding the exact underlying contact network structure, precise transmission links, latency, and individual recovery times, we believe our modeling framework represents an unbiased and justifiable approach. Specifically, modeling the local transmission clusters as complete graphs reflects the assumption that small infection trees typically generate within dense, highly connected local social groups. Furthermore, because our empirical contact-tracing data does not indicate recovery or "curing" events, adopting an SI (Susceptible-Infectious) process provides a meaningful and robust baseline.

The complete graph assumption and the absence of a curing process overestimate the viral force of a single node to infect its neighbors therefore giving an upper bound on the value of the shape parameter alpha, which is then safely larger than 1.

Additionally, the infection time in our model does not exclusively represent a single biological phase (such as the strict interval from exposure to infectiousness). Rather, it acts as an effective transmission time that inherently captures a convolution of multiple, often unobservable, compounding delays. These delays could span from true biological latency and incubation periods to human behavioral factors, such as symptom reporting delays, testing lags, and the time taken to isolate.

Finally, we appreciate the reviewer pointing out the relevant work by Ogura and Preciado. While their study provides valuable insights into non-Markovian dynamics on networks, we believe our current manuscript already thoroughly frames our approach within the well-established literature by citing several key foundational works on non-Markovian epidemics in networks (specifically, references [19, 20, 21, 22]).

Assumption 2 says that each observed tree of size N represents a complete outbreak in a fully connected community, where all N individuals are eventually infected. This assumption is difficult to match with the manuscript's own description that the traced clusters are sparse and incomplete. Also, this assumption directly defines the benchmark distribution used in the fitting. The manuscript should justify this assumption from an epidemiological viewpoint or relax it. The authors should also position the model more clearly against earlier tree-shape memory studies such as Plazzotta et al. [2].

(R) We thank the reviewer for this important and insightful comment regarding our assumptions and literature positioning.

As noted, we assume that each observed tree of size N represents a complete outbreak in a fully connected community, where all N individuals are eventually infected. In the "Infection trees" section, we explicitly acknowledge that incomplete information is a well-known limitation inherent to real-world contact-tracing data, which naturally

results in multiple fragmented or disconnected transmission chains rather than a single, perfectly unified tree for an entire epidemic wave.

Our local assumption is not incompatible with the sparsity and incompleteness of the data because every small infection trees can be seen as a subtree of the large global transmission network of the epidemic. To remain as conservative as possible, we refrain from introducing speculative or artificial links between these isolated trees to reconstruct a global network. The complete graph assumption is thus local and only applies to the nodes in the local infection trees. From an epidemiological perspective, this represents a spreading process occurring within a small local cluster (such as a household, workplace, or localized gathering). In the absence of highly granular sociodemographic or spatial tracking data, assuming a local complete graph is the most unbiased baseline, implying that any individual within that specific cluster could potentially have infected any other contact.

Regarding the work by Plazzotta et al., we have already cited (ref. [25]) and integrated this reference in the Introduction section of our manuscript (Line 36). We prefer not to go too much in detail in the comparison with their modeling approach because of two main reasons: (1) Plazzotta et al. employ a Crump-Mode-Jagers branching process generated by independent point processes for each infected individual; in contrast, our approach utilizes a network-based process that explicitly captures the competitive dynamics between multiple infected individuals attempting to transmit the virus to common, finite neighbors; (2) they apply their model to phylogenetic trees derived from genetic sequences, whereas our study directly analyzes empirical transmission trees reconstructed from real-world epidemiological contact-tracing data. We thank the reviewer for highlighting this valuable reference, and we are glad that its relevance is already reflected in our current manuscript.

The Discussion already notes that missing links near the root and missing links near the leaves can bias the estimated α in opposite directions. Therefore, the sign of the bias is not known a priori from the data. I think a formal sensitivity study with simulated missing-link mechanisms would be important before $\alpha>1$ is interpreted as evidence for non-Markovian infection times. Incomplete tracing and heterogeneity in real outbreaks can affect the observed transmission-network structure, and therefore also the reconstructed tree summaries [3]. These effects should be quantified by applying the same pipeline to simulated and empirical trees, if possible.

(R) We are grateful to the reviewer for this insightful comment regarding the potential competing biases introduced by missing links in the infection trees.

While we agree that a formal sensitivity study incorporating simulated missing-link mechanisms would provide valuable results, we firmly believe that constructing and

validating such a simulation framework falls outside the scope of the current study. Our primary objective here is to introduce a topological method applied directly to the infection trees, quantifying the non-Markovianity of the underlying spreading process.

Accurately modeling, quantifying, and correcting for missing links requires a sophisticated network reconstruction methodology. To address this exact challenge, some of the authors of this paper have been working on advanced link-prediction tools specifically designed to reconstruct missing links within this empirical Cyprus dataset (refs. [47, 48]). Merging these predictive algorithms with our non-Markovian inference represents a major, self-contained research direction that we intend to tackle in a dedicated future work.

Regarding the study by K. Sun et al., we completely agree that it offers an excellent and highly relevant demonstration of how incomplete tracing and outbreak heterogeneity alter observed transmission network structures. This is precisely why their work is already integrated in our current manuscript (ref. [7]).

References

[1] M. Ogura and V. M. Preciado. Stability of SIS Spreading Processes in Networks with Non-Markovian Transmission and Recovery. IEEE Transactions on Control of Network Systems, 7(1), 349-359, 2020.
[2] G. Plazzotta, C. Kwan, M. Boyd, and C. Colijn. Effects of Memory on the Shapes of Simple Outbreak Trees. Scientific Reports, 6, 21159, 2016.
[3] K. Sun, W. Wang, L. Gao, and others. Transmission Heterogeneities, Kinetics, and Controllability of SARS-CoV-2. Science, 371(6526), eabe2424, 2021.
