# Supplementary material for: Non-Markovian dynamics and effective reproduction number in COVID-19: Evidence from Cyprus contact tracing data
Source: PLoS Comput Biol. 2026 Aug 21;22(8):e1014578. doi: 10.1371/journal.pcbi.1014578 (PMC13496441; doi:10.1371/journal.pcbi.1014578)
Supplement: Peer review history

# Peer review: Decision Letter 0

**DOI:** 10.1371/journal.pcbi.1014578.r001

**Authors:** Roger Kouyos, Xiaomin Wan

5 Mar 2026

PCOMPBIOL-D-25-02049

Non-Markovian dynamics and effective reproduction number in COVID-19: evidence from Cyprus contact tracing data

PLOS Computational Biology

Dear Dr. D'Alessandro,

Thank you for submitting your manuscript to PLOS Computational Biology. After careful consideration, we feel that it has merit but does not fully meet PLOS Computational Biology's publication criteria as it currently stands. Therefore, we invite you to submit a revised version of the manuscript that addresses the points raised during the review process.

Please submit your revised manuscript by May 05 2026 11:59PM. If you will need more time than this to complete your revisions, please reply to this message or contact the journal office at ploscompbiol@plos.org. When you're ready to submit your revision, log on to https://www.editorialmanager.com/pcompbiol/ and select the 'Submissions Needing Revision' folder to locate your manuscript file.

Please include the following items when submitting your revised manuscript:

* A letter that responds to each point raised by the editor and reviewer(s). You should upload this letter as a separate file labeled 'Response to Reviewers'. This file does not need to include responses to formatting updates and technical items listed in the 'Journal Requirements' section below.

* A marked-up copy of your manuscript that highlights changes made to the original version. You should upload this as a separate file labeled 'Revised Manuscript with Track Changes'.

* An unmarked version of your revised paper without tracked changes. You should upload this as a separate file labeled 'Manuscript'.

If you would like to make changes to your financial disclosure, competing interests statement, or data availability statement, please make these updates within the submission form at the time of resubmission. Guidelines for resubmitting your figure files are available below the reviewer comments at the end of this letter

We look forward to receiving your revised manuscript.

Kind regards,

Xiaomin Wan

Academic Editor

PLOS Computational Biology

Roger Kouyos

Section Editor

PLOS Computational Biology

**Additional Editor Comments:**

Please revise the manuscript point by point in accordance with all reviewers’ comments.

**Journal Requirements:**

1) Please ensure that the CRediT author contributions listed for every co-author are completed accurately and in full.

At this stage, the following Authors/Authors require contributions: Matteo D'Alessandro, Elisavet Constantinou, and Piet Van Mieghem. Please ensure that the full contributions of each author are acknowledged in the "Add/Edit/Remove Authors" section of our submission form.

The list of CRediT author contributions may be found here: https://journals.plos.org/ploscompbiol/s/authorship#loc-author-contributions

2) We ask that a manuscript source file is provided at Revision. Please upload your manuscript file as a .doc, .docx, .rtf or .tex. If you are providing a .tex file, please upload it under the item type u2018LaTeX Source Fileu2019 and leave your .pdf version as the item type u2018Manuscriptu2019.

3) Please upload all main figures as separate Figure files in .tif or .eps format. For more information about how to convert and format your figure files please see our guidelines:

https://journals.plos.org/ploscompbiol/s/figures

**Reviewers' comments:**

Reviewer's Responses to Questions

**Comments to the Authors:**

**Please note here if the review is uploaded as an attachment.**

Reviewer #1: This manuscript presents a well-structured and insightful study that reconstructs infection trees across multiple COVID-19 waves using detailed contact tracing data. By extending the Markovian SI infection tree framework to a non-Markovian setting, the authors provide an elegant and rigorous quantification of non-Markovianity through the Weibull shape parameter. The analyses of hopcount and outdegree distributions convincingly demonstrate that real-world spreading processes deviate from exponential assumptions, with infection times better described by Weibull distributions (shape > 1). The proposed pruning technique is appropriate and effectively reduces bias in estimating the effective reproduction number. Overall, this is a coherent and methodologically sound work that offers valuable insights for incorporating Non-Markovian dynamics into epidemic modeling.

A major concern is that the authors choose to use the data with at least 1 offspring case in their contact tracing data. If they use this dataset for the estimation of reproduction number, this will introduce the selection bias, and the estimate of reproduction would be lower than the real value (under-estimation).

Reviewer #2: Authors use contact tracing from Cyprus to construct detailed infection trees of the recent SARS-CoV viral epidemic spread. This allows them to determine to what degree that a Markov model, which assumes that probability of infection follows a exponential distribution, are adequate to describe the observed infection trees. They find that the required prob distribution

more closely follows that of a Weibull distribution with alpha > 1.

Overall I found the paper interesting and useful for the discussion on modelling viral spread using SIR models which typically

use exponential distributions to model the viral transmission rates. I have a couple of points which Authors should address.

Major

The Authors say that alpha > 1 has a better fit to the data then alpha =1 which would be the exponential distribution. This makes sense as infection rate from an infected individual to a non-infected one is not constant but increases after initial exposure, then falls off as the individual enters the recovery phase. So the source of the non-markov behavior is not that the current state depends explicitly on previous states per se, but more that the probability distribution of transmission is not exponential. There are other models which have been used to model SARS-cov spread which look at additional compartments, e.g. a SIER, or Susceptible, Exposed, Infected and recovered. One could in effect model a changing beta for infection rate by using the additional Exposed compartment to assume at this stage infection rate is not maximal, but has a smaller probability (or larger) prior to infection and recovery. It would be useful for the Authors to answer the questions:

1) To what degree is a SIR model with standard Markov assumptions able to reproduce the infection tree data, i.e. how "bad" is it compared with the non-markov Weibull case. And equally, how much does the alpha > 1 case improve the fit to the data. (I am not sure this was brought out effectively in the paper).

2) To what degree does the addition of more compartments (e.g. the Exposed category) improve the Markov type models fit to the data. I dont expect you to go to great lengths making several more compartments, but seeing the inclusion of say the Exposed compartment and its effects on fitting to the data might be useful.

To sum up, since Markov models are very useful because they are easy to model, it would be nice to gauge how terrible (or close enough) they are compared with a more accurate distribution (such as Weibull) since these distributions can sometimes be more difficult to sample.

Reviewer #3: The authors analyzed large-scale COVID contact‑tracing data from Cyprus and reconstructed infection trees across four epidemic waves before Omicron waves. They assessed the degree of non‑Markovianity in transmission using hopcount and outdegree distributions. They further estimated dynamic effective reproduction numbers and compare them with previous model‑based estimates.

The work may be more suitable for a mathematical biology journals such as the Journal of Theoretical Biology or Journal of Mathematical Biology. It is not easy to find insights from the computational biology perspective in this specific work.

I have the following suggestions:

(1) It is unclear whether the authors have incorporated under-reporting within the observed infection tree. It would be helpful to the authors could incorporate the uncertainty in case reporting over each wave into the model.

(2) The public health and biological implications of ‘memory effects’ in disease transmission are unclear. It would be better if the authors could link their memory effects findings to the effectiveness of interventions or population behaviour changes. Otherwise, it is not easy to understand why memory effects in disease transmission is helpful to understand real-world public health problems.

Reviewer #4: Using detailed contact-tracing data from Cyprus, this paper reconstructs COVID-19 infection trees across the first four epidemic waves to test whether transmission follows Markovian (memoryless) assumptions. The authors show that the data are best explained by non-Markovian dynamics. This indicates that real COVID-19 transmission exhibits memory effects.The study also estimates the effective reproduction number directly from the outdegree (number of secondary infections) in the infection trees over time.

The authors use a complex data system (transmission trees) to demonstrate the non-regularity (non-Markovian process) of the behaviors of living organisms and propose a counting approach for the estimation of the number of reproductions. The basic principle is that the behavior of individuals in life sciences is not regular. The principle of statistical modeling is to identify the common part of this behavior due to factors that everyone shares and to consider the individual parts as accidental variations. Markov approaches are used to assume that, in an homogeneous group, everyone transmits in the same way and individual differences are accidental. If different rates of transmission are estimated, this cannot be an estimate that is not attributable to epidemic behavior. Otherwise, it would be useless. I would like to estimate for example a \alpha value as a function of the stringency index, which would be meaningful. Otherwise, shown as it is, this study is not reproductible and will not find big interest out of this specific Cyprian context.

Page 4, L37. The concept of "SI" should be defined here, not on page 8.

Page 6. 11573 were removed because their parent source could not be found. And for imported cases? How can one identify the roots then?

Page 6. How are the epidemic waves defined? It is confusing that these are not contiguous? Aren't the period between two consecutive waves considered as part of the epidemic period?

Page 7. We don't see difference between domestic and imported cases in trees?

Page 7. It feels like trees are built in the waves independently. One should be able to draw a trajectory from patient zero to the last infected patient. Here we have isolated trees. Maybe this should be discussed more.

Page 7. The authors did talk about public health measures and analyzed stringency indexes in the supplementary material. How are these taken into account in modelling?

Page 13. How does the model take into account the period of infectivity? As well defined, the reproductive number estimate the infections in a given period. Is that considered somewhere in the estimation?

Page 13 L263. The definition of the effective reproduction number should be reconsidered. It is not the estimate of the basic reproduction number.

Page 14. It cannot be assumed that all trees have the same length N. The trees are supposed random, with random number of leaves. Explain how these can be considered with fixed length.

Page 15. We have the estimate \alpha, what about \beta?

Page 16. Finally, the reproduction numbers are determined by counting. So I wonder about the usefulness of the whole modeling approach. The Markovian assumption was rejected, but no other modeling approach is proposed. Then the method cannot be reproduced elsewhere.

Page17 L349. Fig. is used 2 times in the reference

Reviewer #5: This manuscript constructs infection trees for the first four waves of the COVID-19 epidemic based on contact tracing data provided by the Cyprus Ministry of Health, revealing the non-Markovian dynamics of epidemic transmission. Furthermore, the authors estimate the effective reproduction number using the structure of infection trees and compare it with official model results. The research has strong practical significance and academic value, with a reasonable methodological design and complete content. However, some concepts and conclusions require further explanation and clarification. Specific suggestions are as follows:

1. Regarding the explanation of "hopcount": It is recommended to illustrate the concept of "hopcount" with examples to help readers intuitively understand its meaning within infection trees.

2. Regarding the explanation of research innovation: There is currently a consensus that non-Markovian models, characterized by non-exponentially distributed infection and recovery times, can more realistically describe epidemic transmission processes. Please further clarify which conclusions this paper has drawn that were not explicitly identified or quantified in previous studies.

3. Regarding the definition of "infection times": It is recommended to clarify the specific definition of "infection times," for example, whether it refers to the interval from exposure to becoming infectious, or the time from infection occurrence to being recorded.

4. Regarding the explanation of model equivalence: The manuscript states: "We employ the equivalence between the non-Markovian SI process in a contact graph and the shortest path problem in a graph: an infection tree is equivalent to a shortest path tree if infection times correspond to link weights." It is suggested to further explain the theoretical basis and applicable conditions of this equivalence to enhance understanding for non-specialist readers.

5. Regarding the handling of isolated cases: Please explain how isolated cases with an unidentifiable source of infection were handled when constructing the infection trees, and the potential impact of this handling on subsequent analyses.

6. Regarding the variation in the number of infection trees across epidemic waves: The number of infection trees varies considerably across the four waves. Please analyze the possible reasons for this phenomenon (e.g., changes in transmission patterns, impact of control policies, data recording methods).

7. Regarding the impact of interventions: In the presence of non-pharmaceutical interventions, how can one distinguish between the basic reproduction number under natural transmission and the effective reproduction number under controlled conditions? It is recommended to clarify this point.

8. Regarding the impact of testing and confirmation time: The actual reported time of secondary cases depends not only on the infection time but also on delays in testing and confirmation. Has this factor been considered or discussed in the manuscript? Could the testing and confirmation time affect the estimation of relevant distributions?

9. Regarding sample selection in the Results section: At the beginning of the Results section, it is recommended to explain why N = 5, 6, 7, 8 were selected for analysis. Additionally, why does Figure 7 only show results for waves 1, 3, and 4? Please provide a supplementary explanation.

10. Regarding the readability of data in Figure 7: Please explain more clearly in the figure legend or the main text the meaning of each data point in Figure 7, such as the specific values represented by the size and color of the points (e.g., 3, 24, 240) and their statistical significance.

11. Regarding the basis for the pruning analysis conclusion: The manuscript states: "From the results in Fig.11 the estimate without the last level is the one which can reasonably reflect the evolution of the epidemic wave." How is it determined that this estimate is more "reasonable"? Is there a true reproduction number available as a reference?

12. Regarding the clarity of method comparison: The \alpha estimates obtained by the two methods (based on hopcount distribution and root outdegree distribution) show some differences (as shown in Figure 7). It is recommended to add a comparative analysis of the statistical power and robustness of the two methods, especially their performance when data is sparse, and to explain why the confidence intervals for one method are wider in certain cases.

Reviewer #6: The article describes methods for reconstruction of infection trees from contract-tracing data and their use for estimation of epidemiological parameters (e.g., the reproduction number) and for accessing whether the epidemiological process is Markovian. Authors apply these methods to very rare and complete data from COVID-19 epidemic in Cyprus, and show that the process is non-Markovian, and that their method allows estimating a lower bound for the reproduction number. This is indeed a very rare dataset, which makes very interesting analyses possible.

I see three points that need to be considered or explained in the article:

1. Authors found out that the process is non-Markovian but Weibull. The process they consider is SI. However, COVID-19 features an incubation period, which makes it SEIR. One can model incubation with an exponential distribution and then infectious period with another exponential distribution. The sum on both will not be an exponential distribution and can explain the non-Markovian part. I think it is important to assess/discuss the impact of incubation here.

2. It was not fully clear to me how the authors deal with the ongoing epidemic. Do they assume that they have all the population and the epidemic has stopped? (Assumption 2?) How realistic is that? If not, then there is a problem of censoring: one cannot just count the outdegree of nodes (e.g., lines 266-268) as some of the nodes might still be infectious and might increase their degree in the future. This needs to be properly accounted for (e.g., with survival analysis). The same issue arises when picking the values of delta t for the reproduction number estimation (as the authors state in lines 331-335). The choice of delta t values should be further explained.

3. The choice of treating different trees independently instead of considering that there are missing links between them is another assumption whose consequences should be further discussed. What does it mean from a practical point of view? Different independent introductions to Cyprus, is it realistic?

Another consideration to take into account is that there is a whole field of phylodynamics dealing with SIR-like models on transmission trees (see doi:10.1016/j.jtbi.2010.09.010 for example). If there are sampling and infection times available in the contact-tracing data from Cyprus, one could transform them into time-scaled transmission trees and apply phylodynamic models.

Minor:

Line 37: SI process is mentioned for the first time. Introduce the acronym please.

Fig. 1: It would be helpful to layout the zoomed-in trees without intersections in their branches.

Line 212 and elsewhere: Please put quotes around the section name as otherwise it is not clear where the name of the section finishes.

**********

**Have the authors made all data and (if applicable) computational code underlying the findings in their manuscript fully available?**

The PLOS Data policy requires authors to make all data and code underlying the findings described in their manuscript fully available without restriction, with rare exception (please refer to the Data Availability Statement in the manuscript PDF file). The data and code should be provided as part of the manuscript or its supporting information, or deposited to a public repository. For example, in addition to summary statistics, the data points behind means, medians and variance measures should be available. If there are restrictions on publicly sharing data or code —e.g. participant privacy or use of data from a third party—those must be specified.

Reviewer #1: None

Reviewer #2: Yes

Reviewer #3: **No:** The authors also provide aggregated data for generating figures in the manuscript. Could not find original case data and infection tree data for reproducing their results

Reviewer #4: Yes

Reviewer #5: None

Reviewer #6: **No:** I could not access the data on Zenodo and the infection trees are listed as "can be made available upon request"

**********

PLOS authors have the option to publish the peer review history of their article (what does this mean?). If published, this will include your full peer review and any attached files.

If you choose “no”, your identity will remain anonymous but your review may still be made public.

**Do you want your identity to be public for this peer review?**  For information about this choice, including consent withdrawal, please see our Privacy Policy.

Reviewer #1: No

Reviewer #2: No

Reviewer #3: No

Reviewer #4: No

Reviewer #5: No

Reviewer #6: No

[NOTE: If reviewer comments were submitted as an attachment file, they will be attached to this email and accessible via the submission site. Please log into your account, locate the manuscript record, and check for the action link "View Attachments". If this link does not appear, there are no attachment files.]

**Figure resubmission:**

While revising your submission, we strongly recommend that you use PLOS’s NAAS tool (https://ngplosjournals.pagemajik.ai/artanalysis) to test your figure files. NAAS can convert your figure files to the TIFF file type and meet basic requirements (such as print size, resolution), or provide you with a report on issues that do not meet our requirements and that NAAS cannot fix.

After uploading your figures to PLOS’s NAAS tool - https://ngplosjournals.pagemajik.ai/artanalysis, NAAS will process the files provided and display the results in the "Uploaded Files" section of the page as the processing is complete. If the uploaded figures meet our requirements (or NAAS is able to fix the files to meet our requirements), the figure will be marked as "fixed" above. If NAAS is unable to fix the files, a red "failed" label will appear above. When NAAS has confirmed that the figure files meet our requirements, please download the file via the download option, and include these NAAS processed figure files when submitting your revised manuscript.

**Reproducibility:**

To enhance the reproducibility of your results, we recommend that authors of applicable studies deposit laboratory protocols in protocols.io, where a protocol can be assigned its own identifier (DOI) such that it can be cited independently in the future. Additionally, PLOS ONE offers an option to publish peer-reviewed clinical study protocols. Read more information on sharing protocols at https://plos.org/protocols?utm_medium=editorial-email&utm_source=authorletters&utm_campaign=protocols

# Author comment: Author response to Decision Letter 1

**DOI:** 10.1371/journal.pcbi.1014578.r002

14 May 2026

Attachment Submitted filename: Response to Reviewers.pdf

# Peer review: Decision Letter 1

**DOI:** 10.1371/journal.pcbi.1014578.r003

**Authors:** Roger Kouyos, Xiaomin Wan

24 Jun 2026

PCOMPBIOL-D-25-02049R1

Non-Markovian dynamics and effective reproduction number in COVID-19: evidence from Cyprus contact tracing data

PLOS Computational Biology

Dear Dr. D'Alessandro,

Thank you for submitting your manuscript to PLOS Computational Biology. After careful consideration, we feel that it has merit but does not fully meet PLOS Computational Biology's publication criteria as it currently stands. Therefore, we invite you to submit a revised version of the manuscript that addresses the points raised during the review process.

Please submit your revised manuscript by Aug 24 2026 11:59PM. If you will need more time than this to complete your revisions, please reply to this message or contact the journal office at ploscompbiol@plos.org. When you're ready to submit your revision, log on to https://www.editorialmanager.com/pcompbiol/ and select the 'Submissions Needing Revision' folder to locate your manuscript file.

Please include the following items when submitting your revised manuscript:

* A letter that responds to each point raised by the editor and reviewer(s). You should upload this letter as a separate file labeled 'Response to Reviewers'. This file does not need to include responses to formatting updates and technical items listed in the 'Journal Requirements' section below.

* A marked-up copy of your manuscript that highlights changes made to the original version. You should upload this as a separate file labeled 'Revised Manuscript with Track Changes'.

* An unmarked version of your revised paper without tracked changes. You should upload this as a separate file labeled 'Manuscript'.

If you would like to make changes to your financial disclosure, competing interests statement, or data availability statement, please make these updates within the submission form at the time of resubmission. Guidelines for resubmitting your figure files are available below the reviewer comments at the end of this letter.

As the corresponding author, your ORCID iD is verified in the submission system and will appear in the published article. PLOS supports the use of ORCID, and we encourage all coauthors to register for an ORCID iD and use it as well. Please encourage your coauthors to verify their ORCID iD within the submission system before final acceptance, as unverified ORCID iDs will not appear in the published article. Only the individual author can complete the verification step; PLOS staff cannot verify ORCID iDs on behalf of authors.

We look forward to receiving your revised manuscript.

Kind regards,

Xiaomin Wan

Academic Editor

PLOS Computational Biology

Roger Kouyos

Section Editor

PLOS Computational Biology

**Additional Editor Comments (if provided):**

Please revise the manuscript point by point in accordance with all reviewers’ comments.

**Journal Requirements:**

If the reviewer comments include a recommendation to cite specific previously published works, please review and evaluate these publications to determine whether they are relevant and should be cited. There is no requirement to cite these works unless the editor has indicated otherwise.

1) In the online submission form, you indicated that "Daily COVID-19 case numbers are publicly accessible at https://www.data.gov.cy/. Detailed infection trees can be made available upon request to the Ministry of Health of the Republic of Cyprus (https://www.gov.cy/moh/en/contact/)". All PLOS journals now require all data underlying the findings described in their manuscript to be freely available to other researchers, either

1. In a public repository

2. Within the manuscript itself

3. Uploaded as supplementary information.

This policy applies to all data except where public deposition would breach compliance with the protocol approved by your research ethics board. If your data cannot be made publicly available for ethical or legal reasons (e.g., public availability would compromise patient privacy), please explain your reasons by return email and your exemption request will be escalated to the editor for approval. Your exemption request will be handled independently and will not hold up the peer review process, but will need to be resolved should your manuscript be accepted for publication. One of the Editorial team will then be in touch if there are any issues.

2) <carina-action-element class="ng-star-inserted">Thank you for including an Ethics Statement for your study. Please include:</carina-action-element> <carina-action-element class="ng-star-inserted"></carina-action-element>

<carina-action-element class="ng-star-inserted">i) A statement that formal consent was obtained (must state whether verbal/written) OR the reason consent was not obtained (e.g. anonymity). NOTE: If child participants, the statement must declare that formal consent was obtained from the parent/guardian.].</carina-action-element>

--><carina-action-element class="ng-star-inserted"></carina-action-element>

**Reviewers' comments:**

Reviewer's Responses to Questions

**Comments to the Authors:**

**Please note here if the review is uploaded as an attachment.**

Reviewer #2: The authors have addressed all of the issues raised. I am happy to recommend publication.

Reviewer #6: Thank you for addressing my comments

Reviewer #7: This manuscript uses Cyprus contact-tracing data and constructs infection trees for four COVID-19 waves. Then it compares hopcount and root-outdegree summaries with Weibull SI simulations on complete graphs. The dataset is valuable, and the connection to shortest-path tree theory is interesting. However, I think the main claims are stronger than what the inference setting can support. The estimator for α depends on the complete-graph SI assumption, the strong assumption on completed infection trees, and the selection in contact-tracing data. Also, the part on the effective reproduction number depends on a heuristic pruning rule. Therefore, I recommend major revision.

The abstract states that non-Markovianity can be detected only from the topology of infection trees. However, the inference is conditional on Assumption 1 in the Modeling assumptions section, namely Weibull SI on a complete graph K_N. On the other hand, for example, Ogura and Preciado [1] already study non-Markovian transmission and recovery on networks with richer timing structure. Therefore, the authors should rewrite the main claim as a fit-based statement under this latent model, not as a topology-only detection result.

Assumption 2 says that each observed tree of size N represents a complete outbreak in a fully connected community, where all N individuals are eventually infected. This assumption is difficult to match with the manuscript’s own description that the traced clusters are sparse and incomplete. Also, this assumption directly defines the benchmark distribution used in the fitting. The manuscript should justify this assumption from an epidemiological viewpoint or relax it. The authors should also position the model more clearly against earlier tree-shape memory studies such as Plazzotta et al. [2].

The Discussion already notes that missing links near the root and missing links near the leaves can bias the estimated α in opposite directions. Therefore, the sign of the bias is not known a priori from the data. I think a formal sensitivity study with simulated missing-link mechanisms would be important before α>1 is interpreted as evidence for non-Markovian infection times. Incomplete tracing and heterogeneity in real outbreaks can affect the observed transmission-network structure, and therefore also the reconstructed tree summaries [3]. These effects should be quantified by applying the same pipeline to simulated and empirical trees, if possible.

References

[1] M. Ogura and V. M. Preciado. Stability of SIS Spreading Processes in Networks with Non-Markovian Transmission and Recovery. IEEE Transactions on Control of Network Systems, 7(1), 349-359, 2020.

[2] G. Plazzotta, C. Kwan, M. Boyd, and C. Colijn. Effects of Memory on the Shapes of Simple Outbreak Trees. Scientific Reports, 6, 21159, 2016.

[3] K. Sun, W. Wang, L. Gao, and others. Transmission Heterogeneities, Kinetics, and Controllability of SARS-CoV-2. Science, 371(6526), eabe2424, 2021.

Reviewer #8: The authors have made appropriate revisions in response to the reviewers' feedback, enhancing the article.

**********

**Have the authors made all data and (if applicable) computational code underlying the findings in their manuscript fully available?**

The PLOS Data policy requires authors to make all data and code underlying the findings described in their manuscript fully available without restriction, with rare exception (please refer to the Data Availability Statement in the manuscript PDF file). The data and code should be provided as part of the manuscript or its supporting information, or deposited to a public repository. For example, in addition to summary statistics, the data points behind means, medians and variance measures should be available. If there are restrictions on publicly sharing data or code —e.g. participant privacy or use of data from a third party—those must be specified.

Reviewer #2: Yes

Reviewer #6: Yes

Reviewer #7: Yes

Reviewer #8: None

**********

PLOS authors have the option to publish the peer review history of their article (what does this mean?). If published, this will include your full peer review and any attached files.

If you choose “no”, your identity will remain anonymous but your review may still be made public.

**Do you want your identity to be public for this peer review?**  For information about this choice, including consent withdrawal, please see our Privacy Policy.

Reviewer #2: No

Reviewer #6: No

Reviewer #7: No

Reviewer #8: No

[NOTE: If reviewer comments were submitted as an attachment file, they will be attached to this email and accessible via the submission site. Please log into your account, locate the manuscript record, and check for the action link "View Attachments". If this link does not appear, there are no attachment files.]

**Figure resubmission:**

While revising your submission, we strongly recommend that you use PLOS’s NAAS tool (https://ngplosjournals.pagemajik.ai/artanalysis) to test your figure files. NAAS can convert your figure files to the TIFF file type and meet basic requirements (such as print size, resolution), or provide you with a report on issues that do not meet our requirements and that NAAS cannot fix.

After uploading your figures to PLOS’s NAAS tool - https://ngplosjournals.pagemajik.ai/artanalysis, NAAS will process the files provided and display the results in the "Uploaded Files" section of the page as the processing is complete. If the uploaded figures meet our requirements (or NAAS is able to fix the files to meet our requirements), the figure will be marked as "fixed" above. If NAAS is unable to fix the files, a red "failed" label will appear above. When NAAS has confirmed that the figure files meet our requirements, please download the file via the download option, and include these NAAS processed figure files when submitting your revised manuscript.

**Reproducibility:**

To enhance the reproducibility of your results, we recommend that authors of applicable studies deposit laboratory protocols in protocols.io, where a protocol can be assigned its own identifier (DOI) such that it can be cited independently in the future. Additionally, PLOS ONE offers an option to publish peer-reviewed clinical study protocols. Read more information on sharing protocols at https://plos.org/protocols?utm_medium=editorial-email&utm_source=authorletters&utm_campaign=protocols

# Author comment: Author response to Decision Letter 2

**DOI:** 10.1371/journal.pcbi.1014578.r004

30 Jun 2026

Attachment Submitted filename: Response_to_Reviewers_auresp_2.pdf

# Peer review: Decision Letter 2

**DOI:** 10.1371/journal.pcbi.1014578.r005

**Authors:** Roger Kouyos, Xiaomin Wan

14 Jul 2026

Dear Mr D'Alessandro,

We are pleased to inform you that your manuscript 'Non-Markovian dynamics and effective reproduction number in COVID-19: evidence from Cyprus contact tracing data' has been provisionally accepted for publication in PLOS Computational Biology.

Before your manuscript can be formally accepted you will need to complete some formatting changes, which you will receive in a follow up email. A member of our team will be in touch with a set of requests.

Please note that your manuscript will not be scheduled for publication until you have made the required changes, so a swift response is appreciated.

IMPORTANT: The editorial review process is now complete. PLOS will only permit corrections to spelling, formatting or significant scientific errors from this point onwards. Requests for major changes, or any which affect the scientific understanding of your work, will cause delays to the publication date of your manuscript.

Should you, your institution's press office or the journal office choose to press release your paper, you will automatically be opted out of early publication. We ask that you notify us now if you or your institution is planning to press release the article. All press must be co-ordinated with PLOS.

Thank you again for supporting Open Access publishing; we are looking forward to publishing your work in PLOS Computational Biology.

Best regards,

Xiaomin Wan

Academic Editor

PLOS Computational Biology

Roger Kouyos

Section Editor

PLOS Computational Biology

***********************************************************

Reviewer's Responses to Questions

**Comments to the Authors:**

**Please note here if the review is uploaded as an attachment.**

Reviewer #7: I recommend that the paper be published in its present form.

**********

**Have the authors made all data and (if applicable) computational code underlying the findings in their manuscript fully available?**

The PLOS Data policy requires authors to make all data and code underlying the findings described in their manuscript fully available without restriction, with rare exception (please refer to the Data Availability Statement in the manuscript PDF file). The data and code should be provided as part of the manuscript or its supporting information, or deposited to a public repository. For example, in addition to summary statistics, the data points behind means, medians and variance measures should be available. If there are restrictions on publicly sharing data or code —e.g. participant privacy or use of data from a third party—those must be specified.

Reviewer #7: None

**********

PLOS authors have the option to publish the peer review history of their article (what does this mean?). If published, this will include your full peer review and any attached files.

If you choose “no”, your identity will remain anonymous but your review may still be made public.

**Do you want your identity to be public for this peer review?**  For information about this choice, including consent withdrawal, please see our Privacy Policy.

Reviewer #7: No

# Editor report: Acceptance letter

**DOI:** 10.1371/journal.pcbi.1014578.r006

**Authors:** Roger Kouyos, Xiaomin Wan

PCOMPBIOL-D-25-02049R2

Non-Markovian dynamics and effective reproduction number in COVID-19: evidence from Cyprus contact tracing data

Dear Dr D'Alessandro,

I am pleased to inform you that your manuscript has been formally accepted for publication in PLOS Computational Biology. Your manuscript is now with our production department and you will be notified of the publication date in due course.

The corresponding author will soon be receiving a typeset proof for review, to ensure errors have not been introduced during production. Please review the PDF proof of your manuscript carefully, as this is the last chance to correct any errors. Please note that major changes, or those which affect the scientific understanding of the work, will likely cause delays to the publication date of your manuscript.

Soon after your final files are uploaded, unless you have opted out, the early version of your manuscript will be published online. The date of the early version will be your article's publication date. The final article will be published to the same URL, and all versions of the paper will be accessible to readers.

For Research, Software, and Methods articles, you will receive an invoice from PLOS for your publication fee after your manuscript has reached the completed accept phase. If you receive an email requesting payment before acceptance or for any other service, this may be a phishing scheme. Learn how to identify phishing emails and protect your accounts at https://explore.plos.org/phishing.

Thank you again for supporting PLOS Computational Biology and open-access publishing. We are looking forward to publishing your work!

With kind regards,

Janani Seenivasan

PLOS Computational Biology | Carlyle House, Carlyle Road, Cambridge CB4 3DN | United Kingdom ploscompbiol@plos.org | Phone +44 (0) 1223-442824 | ploscompbiol.org | @PLOSCompBiol
